# Bibliometric Analysis of Research on the Comorbidity of Pain and Inflammation

**DOI:** 10.1155/2021/6655211

**Published:** 2021-02-17

**Authors:** Huan-Yu Xiong, Zhi-Jie Zhang, Xue-Qiang Wang

**Affiliations:** ^1^Department of Sport Rehabilitation, Shanghai University of Sport, 399 Changhai RD, Shanghai 200438, China; ^2^Luoyang Orthopedic Hospital of Henan Province, 82 Qimingnan RD, Luoyang 471000, China; ^3^Department of Rehabilitation Medicine, Shanghai Shangti Orthopaedic Hospital, 188 Hengren RD, Shanghai 200438, China

## Abstract

**Objectives:**

To provide a comprehensive review on the global scientific research status of comorbid pain and inflammation from 1981 to 2019 and capture its subsequent development trends. *Data Sources*. The primary database chosen to collect publications on comorbid pain and inflammation research from 1981 to 2019 was the Web of Science (WOS). Core of the search strategy was the key word “pain” and the key word “inflammation” in the medical subject headings' major field. Study Selection. All articles retrieved were included in the bibliometric analysis. *Data Extraction*. We used CiteSpace to analyze publication outputs, subject categories, distribution by country/institution/journal, and other types of information. Then, knowledge base, hot issues, and future development directions were explained. *Data Synthesis*. A total of 2887 papers met the inclusion criteria in our research. Linear regression analysis results showed that the publications of studies of comorbid pain and inflammation significantly increased (*P* < 0.001) and have grown about 192 times in 40 years. The countries with the most outputs were the USA (886 publications), China (375 publications), and England (236 publications). Besides, Harvard University was the most prolific institution with 730 publications and 6646 citations. In accordance with the subject categories of WOS, neurosciences (31.832%), pharmacology/pharmacy (18.427%), and clinical neurology (15.206%) were the main research areas of these 2887 papers.

**Conclusions:**

The current study reveals that research on comorbid pain and inflammation has gradually become more extensive worldwide since 1981, and neuropathic pain was the most popular study type. Most of our research output in this field came from countries in Europe and North America, although some Asian countries showed promising performance.

## 1. Introduction

Pain as defined by the International Association for the Study of Pain is an unpleasant sensory and emotional experience usually related to actual or potential tissue damage [1–3]. Chronic pain is a growing health problem, which limits the quality of life and employment opportunities of those suffering from this disease and may affect 30% of adults worldwide [4, 5].

In one case, the painful sensation still exists even after the direct cause was alleviated but loses the characteristics of threat and defence and turns into a disease itself. The heterogeneity of its origin makes treatment challenging. None of the currently available treatments can provide sufficient relief for patients with chronic pain; at the same time, aging increases the incidence rate of pain, the risk of complications, and some adverse reactions [6]. Today, the increasing interest in pain management and treatment is closely related to the exponential growth of basic and clinical research on the mechanism of pain occurrence, transmission, and termination [7, 8]. The profound and severe influence of pain often leads to indirect costs, loss of productivity, and increased risk of other diseases. The extent and depth of these consequences will expand and develop as pain continues. According to calculations, the total cost of pain is as high as 3% of the gross domestic product of European countries, which is much higher than the cost of cancer or heart disease [9, 10]; therefore, pain is costly for individual patients, their families, and society as a whole. Although the burden is so heavy, the current treatment for chronic pain is far from enough [11, 12].

Inflammation is a mechanism of nonspecific innate immunity, which is the body's response to injury and infection. Inflammation is a dynamic process that involves the complex biological responses of somatosensory, immune cells, molecular mediators, and vascular systems, and inflammatory actions lead to many disorders [13]. Pain is one of the cardinal features of inflammation [14, 15]. The intensity of pain increases as the degree of inflammation increases. Anti-inflammation drugs are used in the treatment of pain [16]. Even though the neurobiological mechanism of the interaction between pain and inflammation is not yet fully understood, some studies indicate that certain substances might diminish proinflammatory process and then relieve pain [17, 18]. Given the close link between inflammation and pain, we analyzed the current status and trends of comorbid pain and inflammation, especially neuroinflammation [19].

Bibliometric research is an emerging field of information science, which can provide readers with quantitative information regarding the research performed, such as the most influential publications, main countries, core journals, and popular subject categories; it also helps find areas that remain to be studied [20–23]. Additionally, visualization through data-mining technology can also dig out and display valuable information intuitively. Over the past four decades, bibliometrics have been widely applied in various fields, including childhood immunization [24], pain [25–27], functional near-infrared spectroscopy [28], lncRNAs [29], and cancer rehabilitation [30], but only a few have reported on the theme of comorbid pain and inflammation.

The present study aimed to provide a comprehensive review on the global scientific research status of comorbid pain and inflammation research from 1981 to 2019 and capture its subsequent development trends to make up for the deficiencies in the quantitative analysis in this field. We used CiteSpace V to conduct a bibliometric analysis in Web of Science (WOS) Core Collection, which is a tool often applied to assess global scientific research trends and visualization of cocitation networks [31]. The analyses performed by this research focused on the analysis of annual publication outputs, distribution by countries/institutions/journals, and the assessment of the productivity of countries and institutions, which will provide readers with new ideas and valuable information to facilitate cooperation.

## 2. Methods

### 2.1. Search Strategy

We searched for research published in the last 40 years (1981 to 2019). Despite the many databases available for worldwide evaluation research, this study chose the WOS database because it contained rich information, such as the distribution of countries, citations, and subject categories, and had been extensively used in the field of bibliometric analyses.

We conducted a broad search using the key terms: Title = (pain^*∗*^ or headache^*∗*^ or migraine^*∗*^ or headache^*∗*^ or “headache^*∗*^” or cephalalgi^*∗*^ or “abdominal ache^*∗*^” or fibromyalg^*∗*^ or “tummy ache^*∗*^” or “stomachache^*∗*^” or “belly ache^*∗*^” or earache^*∗*^ or earache^*∗*^ or toothache^*∗*^ or toothache^*∗*^ or odontalgi^*∗*^ or neuralgi^*∗*^ or cervicodyn^*∗*^ or analg^*∗*^ or nocicept^*∗*^ or hyperalg^*∗*^ or hypoalg^*∗*^ or radiculalg^*∗*^ or colic or arthralg^*∗*^ or causalg^*∗*^ or maldyn^*∗*^ or eudyn^*∗*^ or ophthalmodyn^*∗*^ or cephalalg^*∗*^ or dysmenorr^*∗*^ or sciatic^*∗*^ or otalg^*∗*^ or brachialg^*∗*^) and Title = (inflammatory or inflammation or inflammations). We included papers published in the form of articles, reviews, letters, and editorial materials for further analysis. The search was restricted to articles in the English language, and no filters of species were specified.

### 2.2. Data Extraction

EndNote (EndNote X9, Bld 7072, Thomson Research Soft, Stamford) and Microsoft Office Excel were used to manage the downloaded results and delete duplicate records. Bibliometric indicators, such as publication count, countries, subject categories, and H-index, were extracted from the raw data to perform the quantitative and qualitative analyses of publications. The definition of the H-index is that a researcher has published *h* papers, and each paper has been cited at least *H* citation times. The H-index has a moderately positive correlation with the number of published papers [32]. In addition, in accordance with the Journal Citation Report (2019), the impact factor (IF) represents the impact of journals.

### 2.3. Analytical Tool

CiteSpace V and Microsoft Excel were used to analyze and evaluate the correlation between some variables (e.g., assessing the productivity of countries and institutions, analyzing annual publication outputs, and determining geographic distributions and partnerships). CiteSpace V, which is widely regarded as an excellent scientometric analysis tool, was used to perform statistical analysis [30]. CiteSpace V could synthesize and visualize cocitation network maps and has helped researchers to discover new trends, hidden meanings, and landmark publications, and cluster analysis and citation burst could be conducted based on cocitation maps. The explosion of citations, which indicates that attention to basic work has increased in a certain period of time, is a critical indicator for identifying emerging trends [33, 34]. The present study used linear regression to analyze the trend over the past four decades. Statistical analyses were performed on the retrieved data with SPSS Statistics 25.0 software. Statistical significance was set at *P* < 0.05.

## 3. Results

### 3.1. Analysis of Publication Outputs

**Figure 1:**
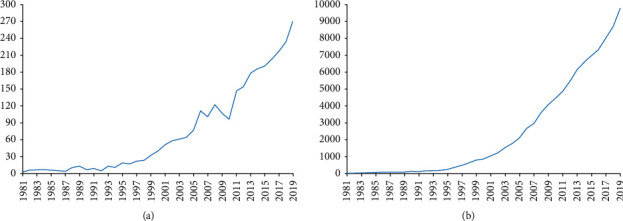
Number of publications and citations. (a) The number of annual publications on pain and inflammation research from 1981 to 2019. (b) The number of annual citations on pain and inflammation research from 1981 to 2019.

**Figure 2:**
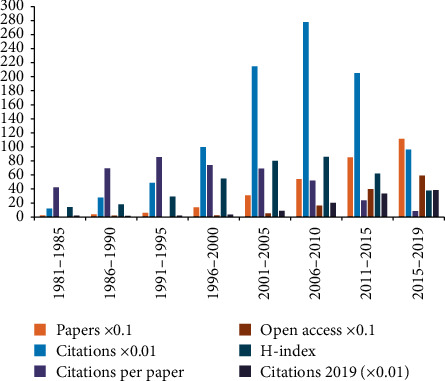
Number of papers, citations, citations per paper, open access paper, H-index, and citations in 2019 for each 5-year time period.

A total of 2,887 papers between 1981 and 2019 met the inclusion criteria. We excluded 1,030 papers (meeting abstracts, book reviews, news items, proceeding papers, notes, and corrections) and 65 non-English papers. Time trend analysis showed that the number of publications rose from 3 in 1981 to 271 in 2019 (Figure 1(a)). The results of linear regression analysis presented the fact that, in the past 40 years, this percentage increased significantly over time (*t* = 14.354, *P* < 0.001). Furthermore, based on the analysis of the line chart, we predicted that the overall growth trend would continue to grow in the future.  Figure 1(b) showed that the 2,887 papers were cited 95,278 times (H-index 131, 33 per year), and the percentage increased over the years (*t* = 12.698, *P* < 0.001). Amongst the eight 5 years (1981–1985, 1986–1990, 1991–1995, 1996–2000, 2001–2005, 2006–2010, 2011–2015, and 2015–2019), 2006–2010 had the most citations (27,820) and the highest H-index value (86), 1991–1995 had the most citations per paper (85.58), and publication outputs (1116) and open access papers (593) in 2015–2019 were the largest (Figure 2). In addition, this result indicated that the intensity of research was constantly growing, and researchers' interest in comorbid pain and inflammation was also developing.

### 3.2. Distribution by Journals

**Figure 3:**
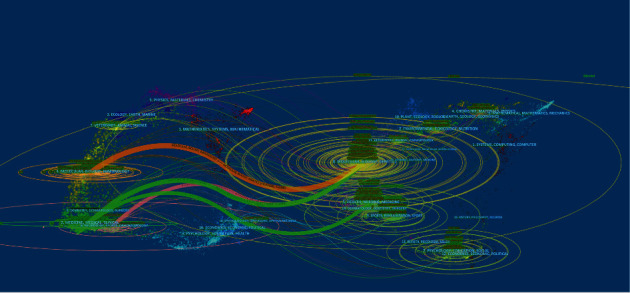
The dual-map overlay of journals related to pain and inflammation research.

The 2,887 references on comorbid pain and inflammation research were distributed amongst 804 scholarly journals. The 20 most influential and active journals accounted for 30.586% of the total publication outputs, which means that they had authority and were recognized as mainstream journals in the field of comorbid pain and inflammation research (Table 1). Pain was the most productive journal (IF 2019, 5.483; 167 articles, 5.785%), followed by Molecular Pain (IF 2019, 2.696; 70 publications, 2.425%) and Journal of Neuroscience (IF 2019, 5.674; 57 publications, 1.974%). The average IF value of the top 20 journals was 5.1656, which implied that these researches were highly reliable. Moreover, in accordance with the journal IF quartile of WOS, 60% of the top 20 journals were Q1 and 35% were Q2.  Figure 3 shows the dual-map overlay map of journals, by which we could know that a large number of the papers were distributed amongst neurology, sports, and ophthalmology journals.

### 3.3. Subject Categories of WOS

**Figure 4:**
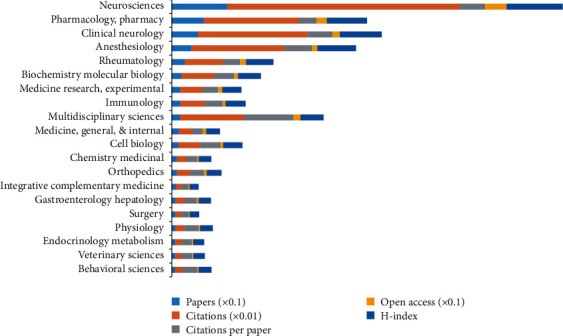
Number of papers, citations, citations per paper, open access papers, and H-index of the top 20 subject categories of WOS.

Subject category could help researchers better understand the focus of the current study [35]. The 2,887 articles were distributed amongst 94 study types.  Figure 4 lists the first 20 subject categories, in which the most popular category was neuroscience with 919 articles (31.832%) and 38,592 citations, followed by pharmacology/pharmacy with 532 articles (18.427%), and clinical neurology with 439 articles (15.206%). Additionally, the results of linear regression analysis indicated that the proportion statistically increased over time (*P* < 0.01) in the top 20 subject categories (*neurosciences, pharmacology/pharmacy, clinical neurology, anesthesiology, rheumatology, biochemistry/molecular biology, medicine research, experimental, immunology, multidisciplinary sciences, medicine general & internal, cell biology, chemistry medicinal, orthopedics, integrative complementary medicine, gastroenterology hepatology, surgery, physiology, endocrinology metabolism, veterinary sciences, and behavioral sciences*).

### 3.4. Types of Pain

**Figure 5:**
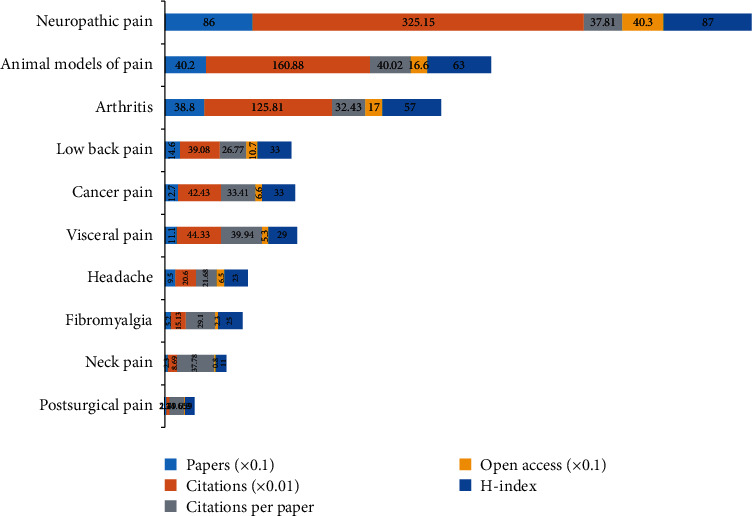
Number of papers, citations, citations per paper, open access papers, and H-index of the top 10 types of pain.

Figure 5 presents the top 10 types of pain by the quantity of publications. Neuropathic pain accounted for the most publication outputs (860, 29.788%) with the most citations (32,515) and the highest H-index value (87), followed by animal models of pain, arthritis, and low back pain. Additionally, the results of the study indicated that the percentage of publications increased significantly over time (*P* < 0.01) in neuropathic pain, animal models of pain, arthritis, low back pain, cancer pain, visceral pain, headache, fibromyalgia, neck pain, and postsurgical pain.

### 3.5. Distribution by Country and Institution

**Figure 6:**
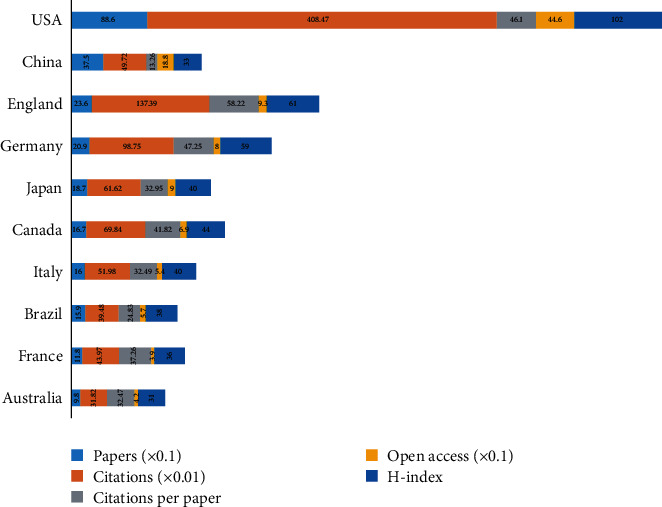
Number of papers, citations, citations per paper, open access papers, and H-index of the top 10 countries.

**Figure 7:**
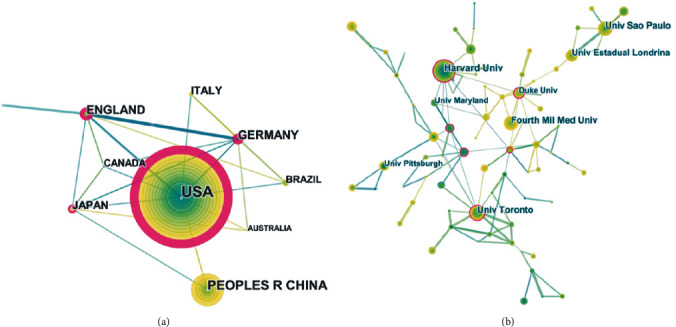
The analysis of countries and institutions. (a) Network map of countries/territories engaged in pain and inflammation research. (b) Network map of institutions engaged in pain and inflammation research.

**Figure 8:**
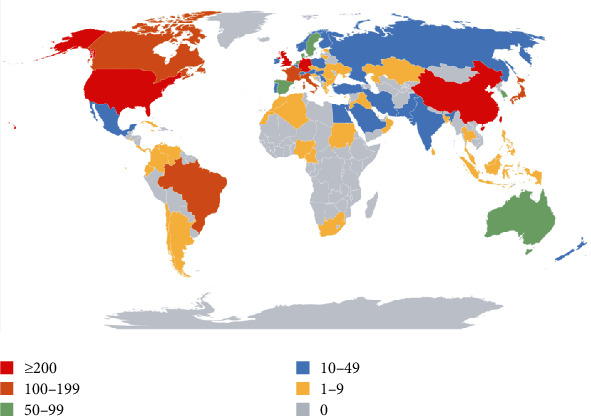
World map of total country output based on pain and inflammation research.

All publications were produced by 87 different countries (Supplementary Table 1).  Figure 6 details the 10 most prolific countries related to comorbid pain and inflammation research, with the top 3 from the USA (886, 30.689%), China (375, 12.989%), and England (236, 8.175%). The countries with the most citations per paper were England (58.22), Germany (47.25), and the USA (46.1). Strikingly, the different countries/regions have strong academic collaborations, which greatly benefits the scientific community [36] (Figure 7(a)).  Figure 8 shows the degree of contribution of countries engaged in the research on a global scale and presents more than 10 publications in 37 out of 87.

A total of 2,582 different institutions contributed to the 2,887 publications from 1981 to 2019 (Supplementary Table 2). The 10 most prolific institutions accounted for 19.079% of the total publication outputs, which indicated that they had authority and achieved considerable research results. Harvard University was the top contributor to comorbid pain and inflammation research with 73 publications and 6,646 total citations, followed by The University of California System (73 publications, 5,249 total citations) and the University of London (72 publications, 6002 total citations).  Figure 7 shows the network visualization map of cooperation between countries and institutions, which implies that the cooperation between institutions was not as obvious as that between countries.

### 3.6. Distribution by Authors

**Figure 9:**
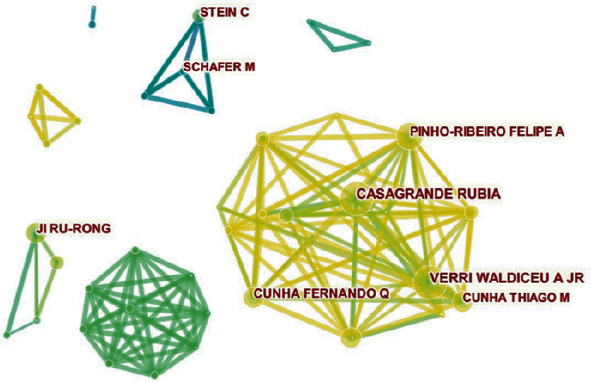
The analysis of authors. Network map of active authors who contributed to pain and inflammation research.

The 2,887 publications on comorbid pain and inflammation research were contributed by 12,402 authors from 1981 to 2019.  Figure 9 outlines the cooperation between authors. Amongst the authors who had the most publications, Verri WA ranked the first with 33 publications, followed by Stein C with 30 publications, Cunha FQ with 25 publications, and Casagrande R with 24 publications.

### 3.7. Analysis of References

**Figure 10:**
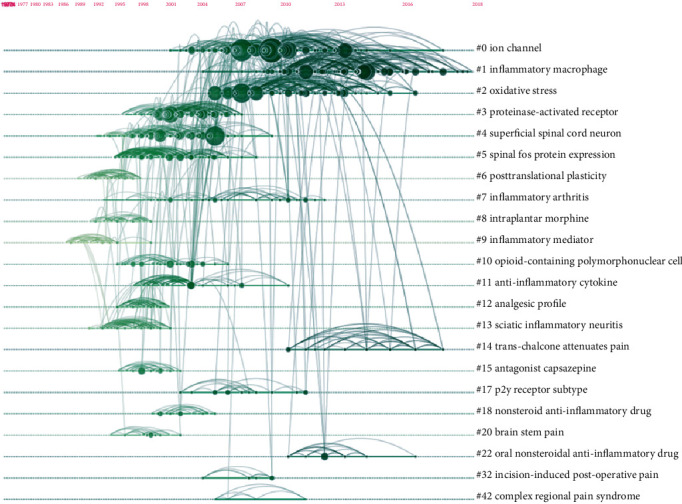
The analysis of references. Cocitation map (timeline view) of references from publications on pain and inflammation research.

The analysis of references was thought to be a critical indicator of bibliometrics research. References with citation bursts constitute a knowledge base that allows researcher to better understand trends in specific research fields [37]. The scientific relevance of publications was described in the cocitation map of references.  Figure 10 shows the top 22 clusters in a timeline view. All clusters were tracked by index terms extracted from the references. The top three clusters were “ion channel” #0, “inflammatory macrophage” #1, and “oxidative stress” #2, respectively.

### 3.8. Analysis of the Most-Cited Articles on Comorbid Pain and Inflammation

The number of citations received for a published paper can be used to prove its influence on the research field.  Table 2 lists the details of the 10 most-cited articles on comorbid pain and inflammation research. The quantity of citations for the 10 most-cited articles ranged from 457 to 1395, which contributed to 7.533% (7,178) of the total quantity of citations. The publication with the most citation frequency (1,395) is “The Formalin Test in Mice-Dissociation between Inflammatory and Noninflammatory Pain” by Hunskaar and Hole [38] published in Pain.

We screened out these 10 references based on their strongest citation bursts, which lasted until 2019 (Table 2), and the citation index would also maintain rapid growth in the next few years. In this way, these articles partly indicated current research hotspots. These results could help researchers go one step further and predict future development direction.

### 3.9. Analysis of Keyword Cooccurrence and Burst Keywords

**Figure 11:**
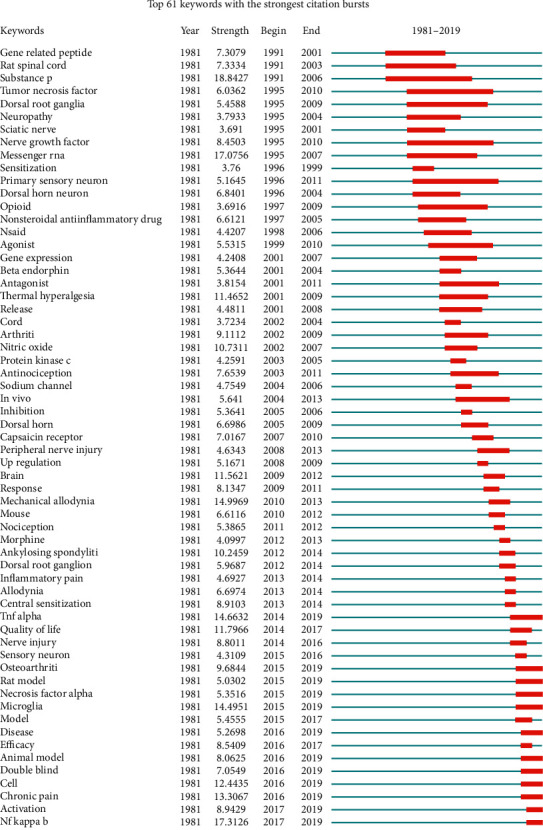
The keywords with the strongest citation bursts of publications on pain and inflammation research.

Keywords with citation bursts can reflect the development of a knowledge field [39]. We used CiteSpace V to generate a network map of keyword cooccurrence.  Figure 11 presents a list of the 61 keywords with the strongest citation bursts since 1981 in this field, in which “gene related peptide”, “rat spinal cord”, “substance p”, and “tumor necrosis factor” were the top 4 keywords. We could predict research frontier through an in-depth understanding of the relationship between keywords.

## 4. Discussion

### 4.1. Global Trends on Comorbid Pain and Inflammation Research

The global trends in publications on comorbid pain and inflammation research presented the notion that statistics continued to grow over time and have grown about 192 times in 40 years. Although the quantity of publications had steadily increased since 1981, percentage of publications between 2004 and 2006 remarkably increased. Amongst the eight 5 years, publication count (1,116) in 2015–2019 was the largest, the number of citations (21,504) in 2001–2005 was the largest, and the H-index value (86) in 2006–2010 was the highest. It is reported that the highest citation counts per year for a paper usually occurs between 3 and 10 years after publication [40]. Therefore, the papers published in recent years collected in this research are expected to receive more researchers' attention in the coming years.

In general, an article with 100 or more citations is often considered a “classic” based on the research field and maybe even a seminal paper [41]; thus, new researchers in a special field could read them before pursuing further research [42]. Therefore, in the current research, 225 papers can be regarded as “classic” papers based on their citation counts, and the citation frequency of the papers ranges from 100 to 1456, which is significantly higher than that of other subjects; of these, 74 papers were more than 200 times. The findings suggest that comorbid pain and inflammation research is a major focus in medicine and health.

As the results indicated, the 2,887 articles were published in 804 scholarly journals, and the 20 most influential journals accounted for 30.586% (883) of the total publication outputs. Pain was the most prolific journal (167 publications, 5.785%), followed by Molecular Pain (70 publications, 2.425%) and Journal of Neuroscience (57 publications; 1.974%). The average IF of most journals is less than six, which indicates that publishing relevant articles in high IF journals is challenging. However, in accordance with the journal IF quartile of WOS, 60% of the top 20 journals were Q1 and 35% were Q2. For beginners in the comorbid pain and inflammation field, the choice of reading papers in these journals allows them to quickly understand the basic principles and track trends.

Most of our research output on comorbid pain and inflammation came from countries in Europe and North America. In accordance with the publication outputs, the USA is at the forefront of studies with 886 publications, followed by China (375), England (236), and Germany (209). These four countries also had the highest frequency of cooperation. Additionally, the geographic visualization analysis of this study distinctly implied the uneven regional development in this field, and developed countries are leading the global comorbid pain and inflammation trend. Similar phenomena were obvious in other fields, such as gastrointestinal medicine [43], asthma [44], and coronary heart disease [45]. On the other hand, we can see some papers from Indonesia in Asia, Sudan in Africa, and Cuba in South America, indicating that comorbid pain and inflammation is widespread on a global scale, and proving once again that this comorbidity has been worth studying. It means that results from all these countries and institutions are shared openly so that more people can benefit from their research advances.

As shown in Figure 7, countries and institutions have massive cooperation and partnership. In total, 2,582 different institutions contributed to the 2,887 publications from 1981 to 2019. The top 10 institutions accounted for 19.079% of total publication outputs, which indicated that they had achieved considerable research results. However, in comparison with the cooperation between countries, the cooperation between these institutions was not obvious.

### 4.2. Research Focus in Comorbid Pain and Inflammation Research

In accordance with the analysis of subject categories of WOS, the most popular research fields were neuroscience (919 publications), pharmacology, pharmacy (532 publications), clinical neurology (439 publications), and anesthesiology (328 publications), thus reflecting a high research interest in this subject in the 21st century. The diagnosis and treatment of comorbid pain and inflammation have developed rapidly in the last few decades. Due to the neural basis of the comorbid pain and inflammation mechanism, it is not surprising that the comorbid pain and inflammation research literature is distributed worldwide in neuroscience in the life sciences and neurology in clinical medicine.

Amongst the top 10 types of pain, the most popular study type was neuropathic pain with the most publications (860), citations (32,515), and the highest H-index value (87). According to the keywords with the strongest citation bursts, our results found that the top 61 keywords since 1981 were as follows: “gene related peptide”, “rat spinal cord”, “substance p”, and “tumor necrosis factor.” Large clusters in keyword cooccurrence networks imply substantial research hotspots [46]. In the past 40 years, as the research covering comorbid pain and inflammation was gradually converted from phenomenon to mechanism, we were optimistic that more effective treatments will be developed in the predictable future.

### 4.3. Study Strengths and Limitations

Identifying classic citations can promote the understanding of the academic development of a specific subject and also help determine emerging themes and future development directions. The current study is the first bibliometric analysis to evaluate the trend of comorbid pain and inflammation research from Science Citation Index Expanded (SCI-Expanded) of WOS over the past four decades. Moreover, the search was not limited to a certain academic journal to obtain rich data. As the results indicated, a total of 2,887 papers were distributed amongst 804 different scholarly journals. In addition, the bibliometric analysis of the present research covered annual publication outputs, most-cited reference, and distribution by countries/institutions/journals and included keyword analysis, popular subject categories, and the productivity of countries and institutions.

Finally, some limitations must be considered. Firstly, we confined the data sources to SCI-Expanded of WOS, and all other electronic databases, such as PubMed and Google Scholar, were excluded. Therefore, some papers collection from WOS database may be delayed, resulting in some bias of citations and H-index in research. Secondly, inherent biases in citation analysis may be present. The citation rate varies by major and depends on the size of the research field. More popular scientific fields, such as diabetes and Alzheimer's disease, often have more classic references. Additionally, authors tend to cite articles in their own language, whilst English articles are more likely to be cited as a whole. Thirdly, bibliometric approach cannot effectively take into account the validity or the scientific rigor of publications. A highly cited publication may not necessarily be of high scientific quality. Despite these limitations, we still believe that the findings provide an effective representative of the research output on comorbid pain and inflammation at a global level.

## 5. Conclusions

This study shows that the research covering comorbid pain and inflammation has gradually become more extensive worldwide between 1981 and 2019 and demonstrates that this research field is well developed with broad prospects. The amounts of publications increased from 3 in 1981 to 271 in 2019, which indicates that the content of this research is constantly being enriched. The most frequent study category was neuroscience. The USA, China, and England constitute the core research forces. Strikingly, countries/regions have extensive collaborations and jointly contributed to research development. In conclusion, this study offers a historical perspective on comorbid pain and inflammation research, which could help us realize the main research countries and institutions, core journals, overall development trend, hotspots, and research frontiers.

## Figures and Tables

**Table 1:** The top 20 journals of origin of papers in the pain and inflammation research.

Journals	Papers	Citations WoS	Citations per paper	Open access	WoS categories	If 2019	Quartile	H-index
Pain	167	11268	67.47	38	Anesthesiology; clinical neurology; Neurosciences	5.483	Q1	58
Molecular Pain	70	1652	23.6	70	Neurosciences	2.696	Q3	25
Journal of Neuroscience	57	4536	79.58	52	Anesthesiology; clinical neurology	5.674	Q1	36
European Journal of Pharmacology	56	2327	41.55	3	Pharmacology pharmacy	3.263	Q2	28
PLoS One	52	1030	19.81	52	Neurosciences; Pharmacology, pharmacy	2.74	Q1	20
European Journal of Pain	47	951	20.23	11	Anesthesiology; clinical neurology; neurosciences	3.492	Q2	17
Neuroscience Letters	44	1940	44.09	3	Neuroscience letters	2.274	Q3	19
Neuroscience	42	2080	49.52	10	Neurosciences	3.056	Q2	24
Journal of Pharmacology								
and Experimental Therapeutics	41	2962	72.24	14	Neurosciences	3.561	Q1	28
British Journal of Pharmacology	38	1942	51.11	36	Pharmacology, pharmacy	7.73	Q1	26
Annals of the Rheumatic Diseases	35	1665	47.57	17	Rheumatology	16.102	Q1	23
Neuropharmacology	33	1174	35.58	8	Neurosciences; pharmacology, pharmacy	4.431	Q2; Q1	23
Brain Research	30	970	32.33	4	Neurosciences	2.733	Q2	16
Journal of Pain	28	994	35.5	10	Clinical neurology; neurosciences	4.621	Q1	19
Anesthesia and Analgesia	26	511	19.65	2	Anesthesiology	4.305	Q2	12
Scientific reports	26	313	12.04	26	Multidisciplinary sciences	3.998	Q1	11
Headache	24	352	14.67	0	Clinical neurology	4.041	Q2	9
Anesthesiology	23	730	31.74	11	Anesthesiology	7.067	Q1	16
Proceedings of the National Academy of Sciences of the USA	23	4837	210.3	23	Multidisciplinary sciences	9.412	Q1	21
Brain, Behavior, and Immunity	21	641	30.52	4	Immunology; neurosciences; psychiatry	6.633	Q1	14

**Table 2:** The top 10 papers with the most citation frequency in the pain and inflammation research.

Title	First author	Journal	Impact factor (IF 2019)	Year	Citations WoS	WoS categories	Category ranking
The Formalin Test in Mice - Dissociation between Inflammatory and Noninflammatory Pain	Hunskaar, S	Pain	5.483	1987	1395	Anesthesiology; clinical neurology; neurosciences	3/31; 21/199; 31/267
Pharmacological and Biochemical Demonstration of the Role of Cyclooxygenase-2 in Inflammation and Pain	Seibert, K	Proceedings of the National Academy of Sciences of the U. S	9.412	1994	1262	Multidisciplinary sciences	7/69
Interleukin-1 beta-Mediated Induction of Cox-2 in the CNS Contributes to Inflammatory Pain Hypersensitivity	Samad, TA	Nature	42.779	2001	928	Multidisciplinary sciences	1/69
Altered Pain Perception and Inflammatory Response in Mice Lacking Prostacyclin Receptor	Murata, T	Nature	42.779	1997	588	Multidisciplinary sciences	1/69
Disruption of the P2X (7) Purinoceptor Gene Abolishes Chronic Inflammatory and Neuropathic Pain	Chessell, IP	Pain	5.483	2005	533	Anesthesiology; clinical neurology; neurosciences	3/31; 21/199; 31/267
Recent Findings on How Proinflammatory Cytokines Cause Pain: Peripheral Mechanisms in Inflammatory and Neuropathic Hyperalgesia	Sommer, C	Neuroscience Letters	2.274	2004	526	Neurosciences	195/267
Bradykinin and Inflammatory Pain	Dray, A	Trends in Neurosciences	12.891	1993	523	Neurosciences	8/267
Immune and Inflammatory Mechanisms in Neuropathic Pain	Moalem, G	Brain Research Reviews	0	2006	489	Neurosciences	29/252
4-Hydroxynonenal, an Endogenous Aldehyde, Causes Pain and Neurogenic Inflammation through Activation of the Irritant Receptor TRPA1	Trevisani, M	Proceedings of the National Academy of Sciences of the U. S	9.412	2007	477	Multidisciplinary sciences	7/69
Immune Activation: The Role of Proinflammatory Cytokines in Inflammation, Illness Responses, and Pathological Pain States	Watkins, LR	Pain	5.483	1995	457	Anesthesiology; clinical neurology; neurosciences	3/31; 21/199; 31/267

## Data Availability

All the datasets generated for this study are included in the article and/or the supplementary files.

## Supplementary Materials

Supplementary Materials Supplementary Figure 1: overview of the paper selection process.Supplementary Figure 2: the number of papers, citations, citations per paper, open access papers, and H-index of the top 10 institutions. Supplementary Table 1: raw data on countries/territories involved in pain and inflammation publications. Supplementary Table 2: raw data on institutions involved in pain and inflammation publications.
