# Supplementary material for: Bibliometric Analysis of Research on the Comorbidity of Pain and Inflammation
Source: Pain Res Manag. 2021 Feb 17;2021:6655211. doi: 10.1155/2021/6655211 (PMC7904349; doi:10.1155/2021/6655211)
Supplement: Supplementary Materials — Supplementary Figure 1: overview of the paper selection process.Supplementary Figure 2: the number of papers, citations, citations per paper, open access papers, and H-index of the top 10 institutions. Supplementary Table 1: raw data on countries/territories involved in pain and inflammation publications. Supplementary Table 2: raw data on institutions involved in pain and inflammation publications. [file 6655211.f1.zip › 6655211.f1/Supplementary Table 1.docx]

**Supplementary Table 1. Raw data on countries/territories involved in pain and inflammation.**

| **Countries/Territories** | **Records** | **% of 2887** |
| --- | --- | --- |
| USA | 886 | 30.689 |
| PEOPLES R CHINA | 375 | 12.989 |
| ENGLAND | 236 | 8.175 |
| GERMANY | 209 | 7.239 |
| JAPAN | 187 | 6.477 |
| CANADA | 167 | 5.785 |
| ITALY | 160 | 5.542 |
| BRAZIL | 159 | 5.507 |
| FRANCE | 118 | 4.087 |
| AUSTRALIA | 98 | 3.395 |
| SOUTH KOREA | 93 | 3.221 |
| NETHERLANDS | 90 | 3.117 |
| SWEDEN | 65 | 2.251 |
| SPAIN | 60 | 2.078 |
| TAIWAN | 59 | 2.044 |
| DENMARK | 58 | 2.009 |
| SWITZERLAND | 46 | 1.593 |
| TURKEY | 41 | 1.42 |
| NORWAY | 39 | 1.351 |
| INDIA | 36 | 1.247 |
| SCOTLAND | 36 | 1.247 |
| AUSTRIA | 34 | 1.178 |
| IRAN | 32 | 1.108 |
| HUNGARY | 29 | 1.005 |
| MEXICO | 29 | 1.005 |
| FINLAND | 28 | 0.97 |
| BELGIUM | 26 | 0.901 |
| POLAND | 20 | 0.693 |
| SAUDI ARABIA | 18 | 0.623 |
| NEW ZEALAND | 17 | 0.589 |
| PORTUGAL | 17 | 0.589 |
| EGYPT | 16 | 0.554 |
| ISRAEL | 16 | 0.554 |
| SERBIA | 16 | 0.554 |
| RUSSIA | 14 | 0.485 |
| IRELAND | 11 | 0.381 |
| SINGAPORE | 11 | 0.381 |
| PAKISTAN | 10 | 0.346 |
| ARGENTINA | 7 | 0.242 |
| LEBANON | 6 | 0.208 |
| UKRAINE | 6 | 0.208 |
| CHILE | 5 | 0.173 |
| CROATIA | 5 | 0.173 |
| CZECH REPUBLIC | 5 | 0.173 |
| GREECE | 5 | 0.173 |
| MALAYSIA | 5 | 0.173 |
| NIGERIA | 5 | 0.173 |
| QATAR | 5 | 0.173 |
| SOUTH AFRICA | 5 | 0.173 |
| CAMEROON | 4 | 0.139 |
| LUXEMBOURG | 4 | 0.139 |
| MOROCCO | 4 | 0.139 |
| ROMANIA | 4 | 0.139 |
| THAILAND | 4 | 0.139 |
| U ARAB EMIRATES | 4 | 0.139 |
| WALES | 4 | 0.139 |
| BANGLADESH | 3 | 0.104 |
| ESTONIA | 3 | 0.104 |
| FED REP GER | 3 | 0.104 |
| INDONESIA | 3 | 0.104 |
| KUWAIT | 3 | 0.104 |
| CYPRUS | 2 | 0.069 |
| JORDAN | 2 | 0.069 |
| KAZAKHSTAN | 2 | 0.069 |
| NORTH IRELAND | 2 | 0.069 |
| OMAN | 2 | 0.069 |
| PHILIPPINES | 2 | 0.069 |
| SLOVENIA | 2 | 0.069 |
| VENEZUELA | 2 | 0.069 |
| ALGERIA | 1 | 0.035 |
| BULGARIA | 1 | 0.035 |
| COLOMBIA | 1 | 0.035 |
| COSTA RICA | 1 | 0.035 |
| CUBA | 1 | 0.035 |
| ECUADOR | 1 | 0.035 |
| EL SALVADOR | 1 | 0.035 |
| HONG KONG | 1 | 0.035 |
| IRAQ | 1 | 0.035 |
| JAMAICA | 1 | 0.035 |
| LITHUANIA | 1 | 0.035 |
| MACEDONIA | 1 | 0.035 |
| PANAMA | 1 | 0.035 |
| SLOVAKIA | 1 | 0.035 |
| SRI LANKA | 1 | 0.035 |
| ST KITTS NEVI | 1 | 0.035 |
| SUDAN | 1 | 0.035 |
| SWAZILAND | 1 | 0.035 |
