# Supplementary material for: Bibliometric Analysis of Research on the Comorbidity of Pain and Inflammation
Source: Pain Res Manag. 2021 Feb 17;2021:6655211. doi: 10.1155/2021/6655211 (PMC7904349; doi:10.1155/2021/6655211)
Supplement: Supplementary Materials — Supplementary Figure 1: overview of the paper selection process.Supplementary Figure 2: the number of papers, citations, citations per paper, open access papers, and H-index of the top 10 institutions. Supplementary Table 1: raw data on countries/territories involved in pain and inflammation publications. Supplementary Table 2: raw data on institutions involved in pain and inflammation publications. [file 6655211.f1.zip › 6655211.f1/Supplementary Table 2.docx]

**Supplementary Table 2. Raw data on institutions involved in pain and inflammation publications.**

| **Institutions** | **records** | **% of 2887** |
| --- | --- | --- |
| HARVARD UNIVERSITY | 73 | 2.528 |
| UNIVERSITY OF CALIFORNIA SYSTEM | 73 | 2.528 |
| UNIVERSITY OF LONDON | 72 | 2.493 |
| INSTITUT NATIONAL DE LA SANTE ET DE LA RECHERCHE MEDICALE INSERM | 61 | 2.112 |
| UNIVERSITY OF TEXAS SYSTEM | 55 | 1.904 |
| CENTRE NATIONAL DE LA RECHERCHE SCIENTIFIQUE CNRS | 47 | 1.627 |
| UNIVERSIDADE DE SAO PAULO | 45 | 1.558 |
| FREE UNIVERSITY OF BERLIN | 43 | 1.489 |
| CHARITE MEDICAL UNIVERSITY OF BERLIN | 41 | 1.42 |
| HUMBOLDT UNIVERSITY OF BERLIN | 41 | 1.42 |
| AIR FORCE MILITARY MEDICAL UNIVERSITY | 39 | 1.35 |
| PENNSYLVANIA COMMONWEALTH SYSTEM OF HIGHER EDUCATION PCSHE | 38 | 1.316 |
| UNIVERSITY COLLEGE LONDON | 38 | 1.316 |
| NATIONAL INSTITUTES OF HEALTH NIH USA | 37 | 1.281 |
| UNIVERSITY OF TORONTO | 37 | 1.281 |
| JOHNS HOPKINS UNIVERSITY | 34 | 1.177 |
| MCGILL UNIVERSITY | 33 | 1.143 |
| UNIVERSITY OF COPENHAGEN | 33 | 1.143 |
| UNIVERSITY SYSTEM OF MARYLAND | 33 | 1.143 |
| LEIDEN UNIVERSITY | 32 | 1.108 |
| UNIVERSIDADE ESTADUAL DE LONDRINA | 32 | 1.108 |
| UNIVERSITY OF MARYLAND BALTIMORE | 32 | 1.108 |
| BRIGHAM WOMEN S HOSPITAL | 30 | 1.039 |
| KINGS COLLEGE LONDON | 30 | 1.039 |
| KAROLINSKA INSTITUTET | 29 | 1.004 |
| UNIVERSITY OF CALIFORNIA SAN FRANCISCO | 29 | 1.004 |
| UNIVERSITY OF PITTSBURGH | 29 | 1.004 |
| RUPRECHT KARLS UNIVERSITY HEIDELBERG | 28 | 0.97 |
| UNIVERSITY OF ARIZONA | 28 | 0.97 |
| CHARITE BEN FRANKLIN CAMPUS | 27 | 0.935 |
| GLAXOSMITHKLINE | 27 | 0.935 |
| UNIVERSITY OF CALGARY | 27 | 0.935 |
| UNIVERSITY OF PENNSYLVANIA | 27 | 0.935 |
| DUKE UNIVERSITY | 26 | 0.9 |
| PEKING UNIVERSITY | 26 | 0.9 |
| PFIZER | 26 | 0.9 |
| UNIVERSIDADE FEDERAL DE SANTA CATARINA UFSC | 26 | 0.9 |
| UNIVERSITY OF ERLANGEN NUREMBERG | 26 | 0.9 |
| ASSISTANCE PUBLIQUE HOPITAUX PARIS APHP | 25 | 0.866 |
| STANFORD UNIVERSITY | 25 | 0.866 |
| CNRS NATIONAL INSTITUTE FOR BIOLOGY INSB | 24 | 0.831 |
| UNIVERSIDADE FEDERAL DE MINAS GERAIS | 24 | 0.831 |
| IMPERIAL COLLEGE LONDON | 23 | 0.796 |
| SEOUL NATIONAL UNIVERSITY SNU | 23 | 0.796 |
| UNIVERSITY OF MILAN | 22 | 0.762 |
| CHIBA UNIVERSITY | 21 | 0.727 |
| INDIANA UNIVERSITY SYSTEM | 21 | 0.727 |
| MASSACHUSETTS GENERAL HOSPITAL | 21 | 0.727 |
| NOVARTIS | 21 | 0.727 |
| UNIVERSITY OF MINNESOTA SYSTEM | 21 | 0.727 |
| UTRECHT UNIVERSITY | 21 | 0.727 |
| MAASTRICHT UNIVERSITY | 20 | 0.693 |
| SAPIENZA UNIVERSITY ROME | 20 | 0.693 |
| UNIVERSITY OF MINNESOTA TWIN CITIES | 20 | 0.693 |
| FUDAN UNIVERSITY | 19 | 0.658 |
| GOETHE UNIVERSITY FRANKFURT | 19 | 0.658 |
| UNIVERSITY OF CALIFORNIA SAN DIEGO | 19 | 0.658 |
| UNIVERSITY OF SYDNEY | 19 | 0.658 |
| MONASH UNIVERSITY | 18 | 0.623 |
| UNIVERSITY OF AMSTERDAM | 18 | 0.623 |
| UNIVERSITY OF NOTTINGHAM | 18 | 0.623 |
| UNIVERSITY OF OSLO | 18 | 0.623 |
| UNIVERSITY OF WURZBURG | 18 | 0.623 |
| WASHINGTON UNIVERSITY WUSTL | 18 | 0.623 |
| ABBOTT LABORATORIES | 17 | 0.589 |
| CHINA MEDICAL UNIVERSITY TAIWAN | 17 | 0.589 |
| CHINESE ACADEMY OF SCIENCES | 17 | 0.589 |
| ERASMUS UNIVERSITY ROTTERDAM | 17 | 0.589 |
| KYUNG HEE UNIVERSITY | 17 | 0.589 |
| UNIVERSITE DE PARIS | 17 | 0.589 |
| UNIVERSITY OF EDINBURGH | 17 | 0.589 |
| UNIVERSITY OF IOWA | 17 | 0.589 |
| UNIVERSITY OF ZURICH | 17 | 0.589 |
| VIRGINIA COMMONWEALTH UNIVERSITY | 17 | 0.589 |
| COLUMBIA UNIVERSITY | 16 | 0.554 |
| MAX PLANCK SOCIETY | 16 | 0.554 |
| NANTONG UNIVERSITY | 16 | 0.554 |
| UNIVERSITY OF HELSINKI | 16 | 0.554 |
| UNIVERSITY OF MELBOURNE | 16 | 0.554 |
| UNIVERSITY OF OXFORD | 16 | 0.554 |
| UNIVERSITY OF QUEENSLAND | 16 | 0.554 |
| UTMD ANDERSON CANCER CENTER | 16 | 0.554 |
| ZHEJIANG CHINESE MEDICAL UNIVERSITY | 16 | 0.554 |
| AARHUS UNIVERSITY | 15 | 0.519 |
| ACADEMIC MEDICAL CENTER AMSTERDAM | 15 | 0.519 |
| CONSIGLIO NAZIONALE DELLE RICERCHE CNR | 15 | 0.519 |
| ERASMUS MC | 15 | 0.519 |
| GOETHE UNIVERSITY FRANKFURT HOSPITAL | 15 | 0.519 |
| HARVARD MEDICAL SCHOOL | 15 | 0.519 |
| HOPITAL UNIVERSITAIRE COCHIN APHP | 15 | 0.519 |
| MERCK COMPANY | 15 | 0.519 |
| UNIVERSITY OF PARIS DESCARTES | 15 | 0.519 |
| UNIVERSITY OF WASHINGTON | 15 | 0.519 |
| UNIVERSITY OF WASHINGTON SEATTLE | 15 | 0.519 |
| INDIANA UNIVERSITY BLOOMINGTON | 14 | 0.485 |
| KOREA UNIVERSITY | 14 | 0.485 |
| LANZHOU UNIVERSITY | 14 | 0.485 |
| SHANGHAI JIAO TONG UNIVERSITY | 14 | 0.485 |
| SORBONNE UNIVERSITE | 14 | 0.485 |
| UNIVERSITE DE TOULOUSE | 14 | 0.485 |
| UNIVERSITY OF ALBERTA | 14 | 0.485 |
| UNIVERSITY OF BELGRADE | 14 | 0.485 |
| UNIVERSITY OF COLORADO SYSTEM | 14 | 0.485 |
| UNIVERSITY OF PARIS DIDEROT | 14 | 0.485 |
| UTRECHT UNIVERSITY MEDICAL CENTER | 14 | 0.485 |
| XUZHOU MEDICAL COLLEGE | 14 | 0.485 |
| ASTRAZENECA | 13 | 0.45 |
| BETH ISRAEL DEACONESS MEDICAL CENTER | 13 | 0.45 |
| CAPITAL MEDICAL UNIVERSITY | 13 | 0.45 |
| CINVESTAV CENTRO DE INVESTIGACION Y DE ESTUDIOS AVANZADOS DEL INSTITUTO POLITECNICO NACIONAL | 13 | 0.45 |
| DALHOUSIE UNIVERSITY | 13 | 0.45 |
| KAROLINSKA UNIVERSITY HOSPITAL | 13 | 0.45 |
| OHIO STATE UNIVERSITY | 13 | 0.45 |
| UNIVERSITE TOULOUSE III PAUL SABATIER | 13 | 0.45 |
| UNIVERSITY OF MICHIGAN | 13 | 0.45 |
| UNIVERSITY OF MICHIGAN SYSTEM | 13 | 0.45 |
| UNIVERSITY OF MUNICH | 13 | 0.45 |
| UNIVERSITY OF NORTH CAROLINA | 13 | 0.45 |
| VRIJE UNIVERSITEIT AMSTERDAM | 13 | 0.45 |
| AALBORG UNIVERSITY | 12 | 0.416 |
| CENTRAL SOUTH UNIVERSITY | 12 | 0.416 |
| CHANG GUNG MEMORIAL HOSPITAL | 12 | 0.416 |
| HUAZHONG UNIVERSITY OF SCIENCE TECHNOLOGY | 12 | 0.416 |
| HUNGARIAN ACADEMY OF SCIENCES | 12 | 0.416 |
| MEDICAL UNIVERSITY OF VIENNA | 12 | 0.416 |
| RIGSHOSPITALET | 12 | 0.416 |
| SHAHID BEHESHTI UNIVERSITY MEDICAL SCIENCES | 12 | 0.416 |
| STATE UNIVERSITY SYSTEM OF FLORIDA | 12 | 0.416 |
| UNIVERSITY HEALTH NETWORK TORONTO | 12 | 0.416 |
| UNIVERSITY OF BONN | 12 | 0.416 |
| UNIVERSITY OF BRISTOL | 12 | 0.416 |
| UNIVERSITY OF FLORENCE | 12 | 0.416 |
| UNIVERSITY OF GLASGOW | 12 | 0.416 |
| ABBOTT VASCULAR | 11 | 0.381 |
| CONSEJO SUPERIOR DE INVESTIGACIONES CIENTIFICAS CSIC | 11 | 0.381 |
| KYUNGPOOK NATIONAL UNIVERSITY | 11 | 0.381 |
| LUND UNIVERSITY | 11 | 0.381 |
| NANJING MEDICAL UNIVERSITY | 11 | 0.381 |
| NEW YORK UNIVERSITY | 11 | 0.381 |
| NIH NATIONAL INSTITUTE ON DRUG ABUSE NIDA | 11 | 0.381 |
| NIHON UNIVERSITY | 11 | 0.381 |
| OREGON HEALTH SCIENCE UNIVERSITY | 11 | 0.381 |
| UNIVERSIDADE FEDERAL DE SANTA MARIA UFSM | 11 | 0.381 |
| UNIVERSITY OF BERGEN | 11 | 0.381 |
| UNIVERSITY OF BERN | 11 | 0.381 |
| UNIVERSITY OF COLORADO BOULDER | 11 | 0.381 |
| UNIVERSITY OF FLORIDA | 11 | 0.381 |
| UNIVERSITY OF KENTUCKY | 11 | 0.381 |
| UNIVERSITY SYSTEM OF GEORGIA | 11 | 0.381 |
| WAKAYAMA MEDICAL UNIVERSITY | 11 | 0.381 |
| BOSTON CHILDREN S HOSPITAL | 10 | 0.346 |
| JUSTUS LIEBIG UNIVERSITY GIESSEN | 10 | 0.346 |
| KOLLING INSTITUTE OF MEDICAL RESEARCH | 10 | 0.346 |
| LAVAL UNIVERSITY | 10 | 0.346 |
| ROYAL NORTH SHORE HOSPITAL | 10 | 0.346 |
| RUSSIAN ACADEMY OF SCIENCES | 10 | 0.346 |
| SEMMELWEIS UNIVERSITY | 10 | 0.346 |
| STATE UNIVERSITY OF NEW YORK SUNY SYSTEM | 10 | 0.346 |
| SUN YAT SEN UNIVERSITY | 10 | 0.346 |
| UNIVERSITA DELLA CAMPANIA VANVITELLI | 10 | 0.346 |
| UNIVERSITY OF ALABAMA BIRMINGHAM | 10 | 0.346 |
| UNIVERSITY OF ALABAMA SYSTEM | 10 | 0.346 |
| UNIVERSITY OF CINCINNATI | 10 | 0.346 |
| UNIVERSITY OF LEEDS | 10 | 0.346 |
| UNIVERSITY OF MONTREAL | 10 | 0.346 |
| UNIVERSITY OF NORTH CAROLINA CHAPEL HILL | 10 | 0.346 |
| UPPSALA UNIVERSITY | 10 | 0.346 |
| VA PALO ALTO HEALTH CARE SYSTEM | 10 | 0.346 |
| ATRIUM MEDICAL CENTER | 9 | 0.312 |
| AUTONOMOUS UNIVERSITY OF BARCELONA | 9 | 0.312 |
| CATHOLIC UNIVERSITY OF KOREA | 9 | 0.312 |
| EMORY UNIVERSITY | 9 | 0.312 |
| ETH ZURICH | 9 | 0.312 |
| JOHANNES GUTENBERG UNIVERSITY OF MAINZ | 9 | 0.312 |
| KYOTO UNIVERSITY | 9 | 0.312 |
| MAYO CLINIC | 9 | 0.312 |
| OSAKA UNIVERSITY | 9 | 0.312 |
| SHANDONG UNIVERSITY | 9 | 0.312 |
| SICHUAN UNIVERSITY | 9 | 0.312 |
| SUZHOU UNIVERSITY | 9 | 0.312 |
| UNIVERSIDADE DO PORTO | 9 | 0.312 |
| UNIVERSIDADE ESTADUAL DE CAMPINAS | 9 | 0.312 |
| UNIVERSITE DE MONTPELLIER | 9 | 0.312 |
| UNIVERSITE DE STRASBOURG | 9 | 0.312 |
| UNIVERSITES DE STRASBOURG ETABLISSEMENTS ASSOCIES | 9 | 0.312 |
| UNIVERSITY OF CALIFORNIA DAVIS | 9 | 0.312 |
| UNIVERSITY OF CAMBRIDGE | 9 | 0.312 |
| UNIVERSITY OF MESSINA | 9 | 0.312 |
| UNIVERSITY OF NEW SOUTH WALES SYDNEY | 9 | 0.312 |
| UNIVERSITY OF TEXAS MEDICAL BRANCH GALVESTON | 9 | 0.312 |
| US DEPARTMENT OF VETERAN AFFAIRS | 9 | 0.312 |
| WAKE FOREST UNIVERSITY | 9 | 0.312 |
| BAYLOR COLLEGE OF MEDICINE | 8 | 0.277 |
| CABRINI HEALTH | 8 | 0.277 |
| CEDARS SINAI MEDICAL CENTER | 8 | 0.277 |
| CHANG GUNG UNIVERSITY | 8 | 0.277 |
| CHINA MEDICAL UNIVERSITY HOSPITAL TAIWAN | 8 | 0.277 |
| CHONNAM NATIONAL UNIVERSITY | 8 | 0.277 |
| CHU CLERMONT FERRAND | 8 | 0.277 |
| DIAKONHJEMMET HOSPITAL | 8 | 0.277 |
| FRIEDRICH SCHILLER UNIVERSITY OF JENA | 8 | 0.277 |
| HEBREW UNIVERSITY OF JERUSALEM | 8 | 0.277 |
| ICAHN SCHOOL OF MEDICINE AT MOUNT SINAI | 8 | 0.277 |
| INSTITUTO POLITECNICO NACIONAL MEXICO | 8 | 0.277 |
| KITASATO UNIVERSITY | 8 | 0.277 |
| NANJING UNIVERSITY | 8 | 0.277 |
| NATIONAL UNIVERSITY OF SINGAPORE | 8 | 0.277 |
| NORTHWELL HEALTH | 8 | 0.277 |
| PAVLOV INSTITUTE OF PHYSIOLOGY RUSSIAN ACADEMY OF SCIENCES | 8 | 0.277 |
| POLISH ACADEMY OF SCIENCES | 8 | 0.277 |
| QUEENS UNIVERSITY CANADA | 8 | 0.277 |
| SCRIPPS RESEARCH INSTITUTE | 8 | 0.277 |
| SHANGHAI UNIVERSITY OF TRADITIONAL CHINESE MEDICINE | 8 | 0.277 |
| ST PETERSBURG SCIENTIFIC CENTRE OF THE RUSSIAN ACADEMY OF SCIENCES | 8 | 0.277 |
| TUFTS UNIVERSITY | 8 | 0.277 |
| UNIVERSIDADE FEDERAL DO CEARA | 8 | 0.277 |
| UNIVERSITY HOSPITAL OF BERN | 8 | 0.277 |
| UNIVERSITY OF CALIFORNIA IRVINE | 8 | 0.277 |
| UNIVERSITY OF CALIFORNIA LOS ANGELES | 8 | 0.277 |
| UNIVERSITY OF FERRARA | 8 | 0.277 |
| UNIVERSITY OF ILLINOIS SYSTEM | 8 | 0.277 |
| UNIVERSITY OF MANCHESTER | 8 | 0.277 |
| UNIVERSITY OF PECS | 8 | 0.277 |
| UNIVERSITY OF TEXAS ARLINGTON | 8 | 0.277 |
| UNIVERSITY OF TEXAS HEALTH SAN ANTONIO | 8 | 0.277 |
| UNIVERSITY OF TEXAS HEALTH SCIENCE CENTER HOUSTON | 8 | 0.277 |
| UNIVERSITY OF TOKYO | 8 | 0.277 |
| UNIVERSITY OF UTAH | 8 | 0.277 |
| UTAH SYSTEM OF HIGHER EDUCATION | 8 | 0.277 |
| VETERANS HEALTH ADMINISTRATION VHA | 8 | 0.277 |
| AMGEN | 7 | 0.242 |
| CORNELL UNIVERSITY | 7 | 0.242 |
| DOKUZ EYLUL UNIVERSITY | 7 | 0.242 |
| EBERHARD KARLS UNIVERSITY OF TUBINGEN | 7 | 0.242 |
| FUJIAN MEDICAL UNIVERSITY | 7 | 0.242 |
| FUNDACAO OSWALDO CRUZ | 7 | 0.242 |
| G D ANNUNZIO UNIVERSITY OF CHIETI PESCARA | 7 | 0.242 |
| GHENT UNIVERSITY | 7 | 0.242 |
| HELSINKI UNIVERSITY CENTRAL HOSPITAL | 7 | 0.242 |
| HOPITAL UNIVERSITAIRE PITIE SALPETRIERE APHP | 7 | 0.242 |
| INDIANA UNIVERSITY PURDUE UNIVERSITY INDIANAPOLIS | 7 | 0.242 |
| INSTITUTO BUTANTAN | 7 | 0.242 |
| JILIN UNIVERSITY | 7 | 0.242 |
| KING SAUD UNIVERSITY | 7 | 0.242 |
| KOREA INSTITUTE OF SCIENCE TECHNOLOGY KIST | 7 | 0.242 |
| NATIONAL CHUNG HSING UNIVERSITY | 7 | 0.242 |
| NATIONAL YANG MING UNIVERSITY | 7 | 0.242 |
| NORTHWESTERN UNIVERSITY | 7 | 0.242 |
| PLA SECOND MILITARY MEDICAL UNIVERSITY | 7 | 0.242 |
| QUEEN MARY UNIVERSITY LONDON | 7 | 0.242 |
| SHANDONG FIRST MEDICAL UNIVERSITY SHANDONG ACADEMY OF MEDICAL SCIENCES | 7 | 0.242 |
| SHANXI MEDICAL UNIVERSITY | 7 | 0.242 |
| TAIPEI VETERANS GENERAL HOSPITAL | 7 | 0.242 |
| TOHOKU UNIVERSITY | 7 | 0.242 |
| ULUDAG UNIVERSITY | 7 | 0.242 |
| UNIVERSIDAD NACIONAL AUTONOMA DE MEXICO | 7 | 0.242 |
| UNIVERSIDADE FEDERAL DO PARANA | 7 | 0.242 |
| UNIVERSITE CLERMONT AUVERGNE ASSOCIES | 7 | 0.242 |
| UNIVERSITE PARIS SACLAY | 7 | 0.242 |
| UNIVERSITY OF BARCELONA | 7 | 0.242 |
| UNIVERSITY OF CATANIA | 7 | 0.242 |
| UNIVERSITY OF CHICAGO | 7 | 0.242 |
| UNIVERSITY OF GOTHENBURG | 7 | 0.242 |
| UNIVERSITY OF GRANADA | 7 | 0.242 |
| UNIVERSITY OF ILLINOIS CHICAGO | 7 | 0.242 |
| UNIVERSITY OF ILLINOIS CHICAGO HOSPITAL | 7 | 0.242 |
| UNIVERSITY OF MUNSTER | 7 | 0.242 |
| UNIVERSITY OF NAPLES FEDERICO II | 7 | 0.242 |
| UNIVERSITY OF NEWCASTLE | 7 | 0.242 |
| UNIVERSITY OF ROCHESTER | 7 | 0.242 |
| UNIVERSITY OF SOUTHERN DENMARK | 7 | 0.242 |
| UNIVERSITY OF WISCONSIN SYSTEM | 7 | 0.242 |
| WEST VIRGINIA UNIVERSITY | 7 | 0.242 |
| YALE UNIVERSITY | 7 | 0.242 |
| ZHEJIANG UNIVERSITY | 7 | 0.242 |
| AIX MARSEILLE UNIVERSITE | 6 | 0.208 |
| BOEHRINGER INGELHEIM | 6 | 0.208 |
| BUDDHIST TZU CHI GENERAL HOSPITAL | 6 | 0.208 |
| CASE WESTERN RESERVE UNIVERSITY | 6 | 0.208 |
| CHINA MEDICAL UNIVERSITY | 6 | 0.208 |
| CHU DE TOULOUSE | 6 | 0.208 |
| CHUNG SHAN MEDICAL UNIVERSITY | 6 | 0.208 |
| DAVID GEFFEN SCHOOL OF MEDICINE AT UCLA | 6 | 0.208 |
| GHENT UNIVERSITY HOSPITAL | 6 | 0.208 |
| HEINRICH HEINE UNIVERSITY DUSSELDORF | 6 | 0.208 |
| HELMHOLTZ ASSOCIATION | 6 | 0.208 |
| HYOGO COLLEGE OF MEDICINE | 6 | 0.208 |
| JEFFERSON UNIVERSITY | 6 | 0.208 |
| KEELE UNIVERSITY | 6 | 0.208 |
| MASHHAD UNIVERSITY MEDICAL SCIENCE | 6 | 0.208 |
| MEDICAL UNIVERSITY OF SOUTH CAROLINA | 6 | 0.208 |
| NATIONAL CHENG KUNG UNIVERSITY | 6 | 0.208 |
| NIH NATIONAL INSTITUTE OF DENTAL CRANIOFACIAL RESEARCH NIDCR | 6 | 0.208 |
| NIH NATIONAL INSTITUTE ON AGING NIA | 6 | 0.208 |
| NOTTINGHAM CITY HOSPITAL | 6 | 0.208 |
| NOTTINGHAM UNIVERSITY HOSPITAL NHS TRUST | 6 | 0.208 |
| QINGDAO UNIVERSITY | 6 | 0.208 |
| RHEUMAZENTRUM RUHRGEBIET | 6 | 0.208 |
| RUHR UNIVERSITY BOCHUM | 6 | 0.208 |
| SAINT LOUIS UNIVERSITY | 6 | 0.208 |
| TEHRAN UNIVERSITY OF MEDICAL SCIENCES | 6 | 0.208 |
| TEMPLE UNIVERSITY | 6 | 0.208 |
| TOKYO MEDICAL DENTAL UNIVERSITY TMDU | 6 | 0.208 |
| TULANE UNIVERSITY | 6 | 0.208 |
| UNIVERSIDAD MIGUEL HERNANDEZ DE ELCHE | 6 | 0.208 |
| UNIVERSIDADE FEDERAL DO RIO DE JANEIRO | 6 | 0.208 |
| UNIVERSITA DI MODENA E REGGIO EMILIA | 6 | 0.208 |
| UNIVERSITE CLERMONT AUVERGNE UCA | 6 | 0.208 |
| UNIVERSITE DE BORDEAUX | 6 | 0.208 |
| UNIVERSITY OF BOLOGNA | 6 | 0.208 |
| UNIVERSITY OF CONNECTICUT | 6 | 0.208 |
| UNIVERSITY OF DEBRECEN | 6 | 0.208 |
| UNIVERSITY OF FUKUI | 6 | 0.208 |
| UNIVERSITY OF GUELPH | 6 | 0.208 |
| UNIVERSITY OF KANSAS | 6 | 0.208 |
| UNIVERSITY OF KANSAS MEDICAL CENTER | 6 | 0.208 |
| UNIVERSITY OF MISSOURI SYSTEM | 6 | 0.208 |
| UNIVERSITY OF PADUA | 6 | 0.208 |
| WESTERN UNIVERSITY UNIVERSITY OF WESTERN ONTARIO | 6 | 0.208 |
| A A BOGOMOLETZ INSTITUTE OF PHYSIOLOGY | 5 | 0.173 |
| AIN SHAMS UNIVERSITY | 5 | 0.173 |
| CHAPEL ALLERTON HOSPITAL | 5 | 0.173 |
| CHINESE ACADEMY OF MEDICAL SCIENCES PEKING UNION MEDICAL COLLEGE | 5 | 0.173 |
| CHONGQING MEDICAL UNIVERSITY | 5 | 0.173 |
| CHUNG SHAN MEDICAL UNIVERSITY HOSPITAL | 5 | 0.173 |
| CIBER CENTRO DE INVESTIGACION BIOMEDICA EN RED | 5 | 0.173 |
| CLEVELAND CLINIC FOUNDATION | 5 | 0.173 |
| CONSEJO NACIONAL DE INVESTIGACIONES CIENTIFICAS Y TECNICAS CONICET | 5 | 0.173 |
| DREXEL UNIVERSITY | 5 | 0.173 |
| ELI LILLY | 5 | 0.173 |
| FEINBERG SCHOOL OF MEDICINE | 5 | 0.173 |
| GUY S ST THOMAS NHS FOUNDATION TRUST | 5 | 0.173 |
| HALLYM UNIVERSITY | 5 | 0.173 |
| HOKKAIDO UNIVERSITY | 5 | 0.173 |
| HOPITAL UNIVERSITAIRE AMBROISE PARE APHP | 5 | 0.173 |
| HOPITAL UNIVERSITAIRE HENRI MONDOR APHP | 5 | 0.173 |
| INSTITUTO NACIONAL DE PSIQUIATRIA RAMON DE LA FUENTE MUNIZ | 5 | 0.173 |
| IRCCS NEUROMED | 5 | 0.173 |
| KAGOSHIMA UNIVERSITY | 5 | 0.173 |
| KANSAI MEDICAL UNIVERSITY | 5 | 0.173 |
| KOREA INSTITUTE OF ORIENTAL MEDICINE KIOM | 5 | 0.173 |
| KU LEUVEN | 5 | 0.173 |
| KYOTO PREFECTURAL UNIVERSITY OF MEDICINE | 5 | 0.173 |
| LE RESEAU INTERNATIONAL DES INSTITUTS PASTEUR RIIP | 5 | 0.173 |
| LOUISIANA STATE UNIVERSITY SYSTEM | 5 | 0.173 |
| MACKAY MEMORIAL HOSPITAL | 5 | 0.173 |
| MAX DELBRUCK CENTER FOR MOLECULAR MEDICINE | 5 | 0.173 |
| MEDICAL COLLEGE OF WISCONSIN | 5 | 0.173 |
| MEDICAL UNIVERSITY LODZ | 5 | 0.173 |
| NAGOYA CITY UNIVERSITY | 5 | 0.173 |
| NAGOYA UNIVERSITY | 5 | 0.173 |
| NANJING UNIVERSITY OF CHINESE MEDICINE | 5 | 0.173 |
| NATIONAL INSTITUTES OF NATURAL SCIENCES NINS JAPAN | 5 | 0.173 |
| NATIONAL TAIWAN UNIVERSITY | 5 | 0.173 |
| NATIONAL UNIVERSITY OF CORDOBA | 5 | 0.173 |
| NEWCASTLE UNIVERSITY UK | 5 | 0.173 |
| NIH NATIONAL INSTITUTE OF NURSING RESEARCH NINR | 5 | 0.173 |
| NIH NATIONAL INSTITUTE ON ALCOHOL ABUSE ALCOHOLISM NIAAA | 5 | 0.173 |
| NORWEGIAN UNIVERSITY OF SCIENCE TECHNOLOGY NTNU | 5 | 0.173 |
| PUSAN NATIONAL UNIVERSITY | 5 | 0.173 |
| ROYAL MELBOURNE HOSPITAL | 5 | 0.173 |
| SACKLER FACULTY OF MEDICINE | 5 | 0.173 |
| SINAI HEALTH SYSTEM TORONTO | 5 | 0.173 |
| ST GEORGES UNIVERSITY LONDON | 5 | 0.173 |
| TAIPEI MEDICAL UNIVERSITY | 5 | 0.173 |
| TARBIAT MODARES UNIVERSITY | 5 | 0.173 |
| TEL AVIV UNIVERSITY | 5 | 0.173 |
| UNIVERSIDADE ESTADUAL PAULISTA | 5 | 0.173 |
| UNIVERSITE CATHOLIQUE LOUVAIN | 5 | 0.173 |
| UNIVERSITY OF BATH | 5 | 0.173 |
| UNIVERSITY OF DUISBURG ESSEN | 5 | 0.173 |
| UNIVERSITY OF GEORGIA | 5 | 0.173 |
| UNIVERSITY OF HONG KONG | 5 | 0.173 |
| UNIVERSITY OF PARMA | 5 | 0.173 |
| UNIVERSITY OF ROSTOCK | 5 | 0.173 |
| UNIVERSITY OF SHERBROOKE | 5 | 0.173 |
| UNIVERSITY OF SIENA | 5 | 0.173 |
| UNIVERSITY OF TEXAS SOUTHWESTERN MEDICAL CENTER DALLAS | 5 | 0.173 |
| UNIVERSITY OF ULSAN | 5 | 0.173 |
| UNIVERSITY OF WISCONSIN MADISON | 5 | 0.173 |
| XI AN JIAOTONG UNIVERSITY | 5 | 0.173 |
| ACADEMY OF MILITARY MEDICAL SCIENCES CHINA | 4 | 0.139 |
| ALEXANDRIA UNIVERSITY | 4 | 0.139 |
| AMERICAN UNIVERSITY OF BEIRUT | 4 | 0.139 |
| ARATANA THERAPEUT INC | 4 | 0.139 |
| AUTONOMOUS UNIVERSITY OF MADRID | 4 | 0.139 |
| BEIJING NORMAL UNIVERSITY | 4 | 0.139 |
| BEIJING UNIVERSITY OF CHINESE MEDICINE | 4 | 0.139 |
| CANADIAN MEM CHIROPRACT COLL | 4 | 0.139 |
| CARDIFF UNIVERSITY | 4 | 0.139 |
| CATHOLIC UNIVERSITY OF THE SACRED HEART | 4 | 0.139 |
| CHAIM SHEBA MEDICAL CENTER | 4 | 0.139 |
| CHINA JAPAN FRIENDSHIP HOSPITAL | 4 | 0.139 |
| CHINA PHARMACEUTICAL UNIVERSITY | 4 | 0.139 |
| CHINESE PEOPLE S LIBERATION ARMY GENERAL HOSPITAL | 4 | 0.139 |
| CHINESE UNIVERSITY OF HONG KONG | 4 | 0.139 |
| CHU BESANCON | 4 | 0.139 |
| CHU DE NIMES | 4 | 0.139 |
| CHU LYON | 4 | 0.139 |
| CHUNGNAM NATIONAL UNIVERSITY | 4 | 0.139 |
| CITY UNIVERSITY OF NEW YORK CUNY SYSTEM | 4 | 0.139 |
| DARTMOUTH COLLEGE | 4 | 0.139 |
| EBERHARD KARLS UNIVERSITY HOSPITAL | 4 | 0.139 |
| ESTEVE | 4 | 0.139 |
| FLINDERS UNIVERSITY SOUTH AUSTRALIA | 4 | 0.139 |
| FLOREY INSTITUTE OF NEUROSCIENCE MENTAL HEALTH | 4 | 0.139 |
| FRAUNHOFER GESELLSCHAFT | 4 | 0.139 |
| GACHON UNIVERSITY | 4 | 0.139 |
| GEORGETOWN UNIVERSITY | 4 | 0.139 |
| HAUKELAND UNIVERSITY HOSPITAL | 4 | 0.139 |
| HIROSHIMA UNIVERSITY | 4 | 0.139 |
| HOPITAL UNIVERSITAIRE BICETRE APHP | 4 | 0.139 |
| HOPITAL UNIVERSITAIRE SAINT ANTOINE APHP | 4 | 0.139 |
| HOSHI UNIVERSITY | 4 | 0.139 |
| HOSP LILLEBAELT | 4 | 0.139 |
| HOSP SPECIAL SURG | 4 | 0.139 |
| HOSPITAL FOR SICK CHILDREN SICKKIDS | 4 | 0.139 |
| HOSPITAL GARCIA DE ORTA | 4 | 0.139 |
| HOSPITAL UNIVERSITARI VALL D HEBRON | 4 | 0.139 |
| HOWARD HUGHES MEDICAL INSTITUTE | 4 | 0.139 |
| HYOGO UNIV HLTH SCI | 4 | 0.139 |
| INRAE | 4 | 0.139 |
| INSTITUT PASTEUR PARIS | 4 | 0.139 |
| IRCCS SANTA LUCIA | 4 | 0.139 |
| ISTA PHARMACEUT INC | 4 | 0.139 |
| ISTITUTO ITALIANO DI TECNOLOGIA IIT | 4 | 0.139 |
| JADAVPUR UNIVERSITY | 4 | 0.139 |
| JAPAN SCIENCE TECHNOLOGY AGENCY JST | 4 | 0.139 |
| JOHNS HOPKINS MEDICINE | 4 | 0.139 |
| KOBE UNIVERSITY | 4 | 0.139 |
| KYUNG HEE UNIVERSITY HOSPITAL | 4 | 0.139 |
| KYUSHU UNIVERSITY | 4 | 0.139 |
| LUDWIG BOLTZMANN INSTITUTE | 4 | 0.139 |
| MEDICAL UNIVERSITY OF INNSBRUCK | 4 | 0.139 |
| MINNEAPOLIS VA HEALTH CARE SYSTEM | 4 | 0.139 |
| NATIONAL CHENG KUNG UNIVERSITY HOSPITAL | 4 | 0.139 |
| NATIONAL DEFENSE MEDICAL CENTER | 4 | 0.139 |
| NATIONAL RESEARCH CENTRE NRC | 4 | 0.139 |
| NATIONAL UNIVERSITY OF IRELAND NUI GALWAY | 4 | 0.139 |
| NIIGATA UNIVERSITY | 4 | 0.139 |
| PLA | 4 | 0.139 |
| RESEARCH TRIANGLE INSTITUTE | 4 | 0.139 |
| RUTGERS STATE UNIVERSITY MEDICAL CENTER | 4 | 0.139 |
| RUTGERS STATE UNIVERSITY NEW BRUNSWICK | 4 | 0.139 |
| SAHLGRENSKA UNIVERSITY HOSPITAL | 4 | 0.139 |
| SHANGHAI INSTITUTES FOR BIOLOGICAL SCIENCES CAS | 4 | 0.139 |
| SHOWA UNIVERSITY | 4 | 0.139 |
| SKANE UNIVERSITY HOSPITAL | 4 | 0.139 |
| SOUTHEAST UNIVERSITY CHINA | 4 | 0.139 |
| ST GEORGE HOSPITAL | 4 | 0.139 |
| STATE UNIVERSITY OF NEW YORK SUNY STONY BROOK | 4 | 0.139 |
| STAVANGER UNIVERSITY HOSPITAL | 4 | 0.139 |
| TAMPERE UNIVERSITY | 4 | 0.139 |
| TEIKYO UNIVERSITY | 4 | 0.139 |
| TELEMARK HOSPITAL TRUST | 4 | 0.139 |
| TEXAS A M UNIVERSITY SYSTEM | 4 | 0.139 |
| TEXAS TECH UNIVERSITY | 4 | 0.139 |
| TEXAS TECH UNIVERSITY HEALTH SCIENCE CENTER | 4 | 0.139 |
| TEXAS TECH UNIVERSITY SYSTEM | 4 | 0.139 |
| TZU CHI UNIVERSITY | 4 | 0.139 |
| UIT THE ARCTIC UNIVERSITY OF TROMSO | 4 | 0.139 |
| UNIV DSCHANG | 4 | 0.139 |
| UNIVERSIDAD DE CHILE | 4 | 0.139 |
| UNIVERSIDADE FEDERAL DE ALFENAS | 4 | 0.139 |
| UNIVERSIDADE FEDERAL DE SAO PAULO UNIFESP | 4 | 0.139 |
| UNIVERSIDADE FEDERAL DE SERGIPE | 4 | 0.139 |
| UNIVERSITE DE RENNES 1 | 4 | 0.139 |
| UNIVERSITY COLLEGE LONDON HOSPITALS NHS FOUNDATION TRUST | 4 | 0.139 |
| UNIVERSITY OF ADELAIDE | 4 | 0.139 |
| UNIVERSITY OF BRESCIA | 4 | 0.139 |
| UNIVERSITY OF BRITISH COLUMBIA | 4 | 0.139 |
| UNIVERSITY OF CAGLIARI | 4 | 0.139 |
| UNIVERSITY OF CHINESE ACADEMY OF SCIENCES CAS | 4 | 0.139 |
| UNIVERSITY OF GRONINGEN | 4 | 0.139 |
| UNIVERSITY OF INNSBRUCK | 4 | 0.139 |
| UNIVERSITY OF LEIPZIG | 4 | 0.139 |
| UNIVERSITY OF LIEGE | 4 | 0.139 |
| UNIVERSITY OF LUXEMBOURG | 4 | 0.139 |
| UNIVERSITY OF MIAMI | 4 | 0.139 |
| UNIVERSITY OF MILANO BICOCCA | 4 | 0.139 |
| UNIVERSITY OF MISSOURI KANSAS CITY | 4 | 0.139 |
| UNIVERSITY OF NORTH CAROLINA SCHOOL OF MEDICINE | 4 | 0.139 |
| UNIVERSITY OF PESHAWAR | 4 | 0.139 |
| UNIVERSITY OF REGENSBURG | 4 | 0.139 |
| UNIVERSITY OF SCIENCE TECHNOLOGY OF CHINA | 4 | 0.139 |
| UNIVERSITY OF SHEFFIELD | 4 | 0.139 |
| UNIVERSITY OF SOUTH AUSTRALIA | 4 | 0.139 |
| UNIVERSITY OF SOUTHAMPTON | 4 | 0.139 |
| UNIVERSITY OF SOUTHERN CALIFORNIA | 4 | 0.139 |
| UNIVERSITY OF TEXAS DALLAS | 4 | 0.139 |
| UNIVERSITY OF TOYAMA | 4 | 0.139 |
| UNIVERSITY OF TURIN | 4 | 0.139 |
| UNIVERSITY OF VIRGINIA | 4 | 0.139 |
| UNIVERSITY OF ZAGREB | 4 | 0.139 |
| VA BOSTON HEALTHCARE SYSTEM | 4 | 0.139 |
| VANDERBILT UNIVERSITY | 4 | 0.139 |
| VITA SALUTE SAN RAFFAELE UNIVERSITY | 4 | 0.139 |
| VRIJE UNIVERSITEIT BRUSSEL | 4 | 0.139 |
| VU UNIVERSITY MEDICAL CENTER | 4 | 0.139 |
| WAKE FOREST BAPTIST MEDICAL CENTER | 4 | 0.139 |
| WAYNE STATE UNIVERSITY | 4 | 0.139 |
| WITTEN HERDECKE UNIVERSITY | 4 | 0.139 |
| ZAGAZIG UNIVERSITY | 4 | 0.139 |
| ABBVIE | 3 | 0.104 |
| ABDUL WALI KHAN UNIVERSITY | 3 | 0.104 |
| ALBANY MEDICAL COLLEGE | 3 | 0.104 |
| ALCON | 3 | 0.104 |
| ANHUI MEDICAL UNIVERSITY | 3 | 0.104 |
| ARKANSAS CHILDREN S HOSPITAL | 3 | 0.104 |
| ASAN MEDICAL CENTER | 3 | 0.104 |
| ASSAM UNIVERSITY | 3 | 0.104 |
| ASSISTANCE PUBLIQUE HOPITAUX DE MARSEILLE | 3 | 0.104 |
| AUGUSTA UNIVERSITY | 3 | 0.104 |
| BBSRC ROSLIN INSTITUTE | 3 | 0.104 |
| BOSTON UNIVERSITY | 3 | 0.104 |
| CATALAN HEALTH INSTITUTE | 3 | 0.104 |
| CHENGDU UNIVERSITY OF TRADITIONAL CHINESE MEDICINE | 3 | 0.104 |
| CHILDRENS HOSPITAL OF PHILADELPHIA | 3 | 0.104 |
| CHINA ACADEMY OF CHINESE MEDICAL SCIENCES | 3 | 0.104 |
| CHU RENNES | 3 | 0.104 |
| CIBERSAM | 3 | 0.104 |
| CNRS INSTITUTE OF CHEMISTRY INC | 3 | 0.104 |
| COLORADO STATE UNIVERSITY | 3 | 0.104 |
| CSIC UMH INSTITUTO DE NEUROCIENCIAS DE ALICANTE IN | 3 | 0.104 |
| DEPT ANESTHESIOL | 3 | 0.104 |
| DOMPE | 3 | 0.104 |
| DOW CHEMICAL COMPANY | 3 | 0.104 |
| DUQUESNE UNIVERSITY | 3 | 0.104 |
| E DA HOSPITAL | 3 | 0.104 |
| EGE UNIVERSITY | 3 | 0.104 |
| EPHE | 3 | 0.104 |
| EUROPEAN MOLECULAR BIOLOGY LABORATORY EMBL | 3 | 0.104 |
| EWHA WOMANS UNIVERSITY | 3 | 0.104 |
| FIRST PEOPLES HOSP YANCHENG | 3 | 0.104 |
| FONDAZIONE IRCCS ISTITUTO NAZIONALE TUMORI MILAN | 3 | 0.104 |
| FU JEN CATHOLIC UNIVERSITY | 3 | 0.104 |
| GANNAN MEDICAL UNIVERSITY | 3 | 0.104 |
| GEORGIA STATE UNIVERSITY | 3 | 0.104 |
| GERIATRIC RESEARCH EDUCATION CLINICAL CENTER | 3 | 0.104 |
| GLASGOW CALEDONIAN UNIVERSITY | 3 | 0.104 |
| GRUNENTHAL GROUP | 3 | 0.104 |
| GUANGXI MEDICAL UNIVERSITY | 3 | 0.104 |
| GUANGZHOU MEDICAL UNIVERSITY | 3 | 0.104 |
| GULHANE MILITARY MEDICAL ACADEMY | 3 | 0.104 |
| HACETTEPE UNIVERSITY | 3 | 0.104 |
| HAMAD MEDICAL CORPORATION | 3 | 0.104 |
| HEBEI MEDICAL UNIVERSITY | 3 | 0.104 |
| HOPITAL UNIVERSITAIRE LARIBOISIERE FERNAND WIDAL APHP | 3 | 0.104 |
| HOSPITAL DEL MAR | 3 | 0.104 |
| HOSPITAL FOR JOINT DISEASE NYULMC | 3 | 0.104 |
| HOSPITAL UNIVERSITARIO LA PAZ | 3 | 0.104 |
| HUNAN INSTITUTE OF SCIENCE TECHNOLOGY | 3 | 0.104 |
| HUNGKUANG UNIVERSITY | 3 | 0.104 |
| I SHOU UNIVERSITY | 3 | 0.104 |
| ICAGEN INC | 3 | 0.104 |
| INSTITUT HOSPITAL DEL MAR D INVESTIGACIONS MEDIQUES IMIM | 3 | 0.104 |
| INSTITUTE OF BASIC MEDICAL SCIENCES CAMS | 3 | 0.104 |
| INSTITUTE OF PSYCHOLOGY CAS | 3 | 0.104 |
| IRCCS POLICLINICO GEMELLI | 3 | 0.104 |
| ISTITUTO DI BIOLOGIA CELLULARE E NEUROBIOLOGIA IBCN CNR | 3 | 0.104 |
| JIKEI UNIVERSITY | 3 | 0.104 |
| KANSAI UNIV HLTH SCI | 3 | 0.104 |
| KAOHSIUNG MEDICAL UNIVERSITY | 3 | 0.104 |
| KING ABDULAZIZ UNIVERSITY | 3 | 0.104 |
| KONKUK UNIVERSITY | 3 | 0.104 |
| KUMAMOTO UNIVERSITY | 3 | 0.104 |
| LINKOPING UNIVERSITY | 3 | 0.104 |
| LOUISIANA STATE UNIVERSITY HEALTH SCIENCES CENTER NEW ORLEANS | 3 | 0.104 |
| LOYOLA UNIVERSITY CHICAGO | 3 | 0.104 |
| MACKAY MEDICAL COLLEGE | 3 | 0.104 |
| MAGNA GRAECIA UNIVERSITY OF CATANZARO | 3 | 0.104 |
| MANHATTAN EYE EAR THROAT HOSPITAL | 3 | 0.104 |
| MCMASTER UNIVERSITY | 3 | 0.104 |
| MEDICAL UNIVERSITY OF WARSAW | 3 | 0.104 |
| MEDIMMUNE | 3 | 0.104 |
| MENZIES INSTITUTE FOR MEDICAL RESEARCH | 3 | 0.104 |
| METROHEALTH SYSTEM | 3 | 0.104 |
| MICHIGAN STATE UNIVERSITY | 3 | 0.104 |
| MIE UNIVERSITY | 3 | 0.104 |
| MOHAMMED VI POLYTECHNIC UNIVERSITY | 3 | 0.104 |
| MURDOCH UNIVERSITY | 3 | 0.104 |
| NATIONAL HOSPITAL NORWAY | 3 | 0.104 |
| NATIONAL INSTITUTE FOR BIOLOGICAL STANDARDS CONTROL | 3 | 0.104 |
| NATIONAL INSTITUTE FOR PHYSIOLOGICAL SCIENCES NIPS | 3 | 0.104 |
| NATIONAL TAIWAN UNIVERSITY HOSPITAL | 3 | 0.104 |
| NATL INST NUTR SEAFOOD RES NIFES | 3 | 0.104 |
| NEUROSEARCH AS | 3 | 0.104 |
| NIH NATIONAL CANCER INSTITUTE NCI | 3 | 0.104 |
| NIH NATIONAL INSTITUTE OF MENTAL HEALTH NIMH | 3 | 0.104 |
| NINGBO UNIVERSITY | 3 | 0.104 |
| NORTH SHORE UNIVERSITY HOSPITAL | 3 | 0.104 |
| NYU LANGONE MEDICAL CENTER | 3 | 0.104 |
| ODENSE UNIVERSITY HOSPITAL | 3 | 0.104 |
| OKAYAMA UNIVERSITY | 3 | 0.104 |
| ONDOKUZ MAYIS UNIVERSITY | 3 | 0.104 |
| OSTFOLD HOSP TRUST | 3 | 0.104 |
| OSTFOLD UNIVERSITY COLLEGE | 3 | 0.104 |
| PANJAB UNIVERSITY | 3 | 0.104 |
| PENNSYLVANIA STATE UNIVERSITY | 3 | 0.104 |
| POCHON CHA UNIVERSITY | 3 | 0.104 |
| QUAID I AZAM UNIVERSITY | 3 | 0.104 |
| QUEEN ELIZABETH HOSP | 3 | 0.104 |
| QUEENSLAND UNIVERSITY OF TECHNOLOGY QUT | 3 | 0.104 |
| RIGSHOSP GLOSTRUP | 3 | 0.104 |
| ROCHE HOLDING | 3 | 0.104 |
| ROYAL MARSDEN NHS FOUNDATION TRUST | 3 | 0.104 |
| SABZEVAR UNIV MED SCI | 3 | 0.104 |
| SAINT MICHAELS HOSPITAL TORONTO | 3 | 0.104 |
| SAMOT | 3 | 0.104 |
| SANOFI AVENTIS | 3 | 0.104 |
| SARHAD UNIV SCI INFORMAT TECHNOL | 3 | 0.104 |
| SEATTLE CHILDREN S HOSPITAL | 3 | 0.104 |
| SEOUL ST MARY S HOSPITAL | 3 | 0.104 |
| STATE UNIVERSITY OF NEW YORK SUNY BUFFALO | 3 | 0.104 |
| SZEGED UNIVERSITY | 3 | 0.104 |
| TEXAS A M UNIVERSITY COLLEGE STATION | 3 | 0.104 |
| TEXAS WOMANS UNIVERSITY | 3 | 0.104 |
| TOKYO METROPOLITAN INSTITUTE OF MEDICAL SCIENCE | 3 | 0.104 |
| TORONTO GENERAL HOSPITAL | 3 | 0.104 |
| TUFTS MEDICAL CENTER | 3 | 0.104 |
| UCL MEDICAL SCHOOL | 3 | 0.104 |
| UNIFORMED SERVICES UNIVERSITY OF THE HEALTH SCIENCES USA | 3 | 0.104 |
| UNITED STATES DEPARTMENT OF DEFENSE | 3 | 0.104 |
| UNIVERSIDAD AUTONOMA METROPOLITANA MEXICO | 3 | 0.104 |
| UNIVERSIDAD DE CADIZ | 3 | 0.104 |
| UNIVERSIDAD DE VALLADOLID | 3 | 0.104 |
| UNIVERSIDAD REY JUAN CARLOS | 3 | 0.104 |
| UNIVERSIDADE FEDERAL DA PARAIBA | 3 | 0.104 |
| UNIVERSIDADE FEDERAL DE GOIAS | 3 | 0.104 |
| UNIVERSIDADE FEDERAL DE SAO CARLOS | 3 | 0.104 |
| UNIVERSITE DE FRANCHE COMTE | 3 | 0.104 |
| UNIVERSITY COLLEGE CORK | 3 | 0.104 |
| UNIVERSITY COLLEGE DUBLIN | 3 | 0.104 |
| UNIVERSITY HOSPITAL OF GIESSEN MARBURG | 3 | 0.104 |
| UNIVERSITY OF ABERDEEN | 3 | 0.104 |
| UNIVERSITY OF ARIZONA HEALTH SCIENCES | 3 | 0.104 |
| UNIVERSITY OF ARKANSAS MEDICAL SCIENCES | 3 | 0.104 |
| UNIVERSITY OF ARKANSAS SYSTEM | 3 | 0.104 |
| UNIVERSITY OF AUCKLAND | 3 | 0.104 |
| UNIVERSITY OF BIRMINGHAM | 3 | 0.104 |
| UNIVERSITY OF BUENOS AIRES | 3 | 0.104 |
| UNIVERSITY OF EAST ANGLIA | 3 | 0.104 |
| UNIVERSITY OF LIVERPOOL | 3 | 0.104 |
| UNIVERSITY OF MASSACHUSETTS SYSTEM | 3 | 0.104 |
| UNIVERSITY OF OKLAHOMA HEALTH SCIENCES CENTER | 3 | 0.104 |
| UNIVERSITY OF OKLAHOMA SYSTEM | 3 | 0.104 |
| UNIVERSITY OF OTAGO | 3 | 0.104 |
| UNIVERSITY OF OTTAWA | 3 | 0.104 |
| UNIVERSITY OF OULU | 3 | 0.104 |
| UNIVERSITY OF PAVIA | 3 | 0.104 |
| UNIVERSITY OF PERUGIA | 3 | 0.104 |
| UNIVERSITY OF PRETORIA | 3 | 0.104 |
| UNIVERSITY OF SOUTH CAROLINA | 3 | 0.104 |
| UNIVERSITY OF SOUTH CAROLINA COLUMBIA | 3 | 0.104 |
| UNIVERSITY OF SOUTH CAROLINA SYSTEM | 3 | 0.104 |
| UNIVERSITY OF TASMANIA | 3 | 0.104 |
| UNIVERSITY OF TURKU | 3 | 0.104 |
| UNIVERSITY OF VALENCIA | 3 | 0.104 |
| UNIVERSITY OF VERONA | 3 | 0.104 |
| UNIVERSITY OF VIENNA | 3 | 0.104 |
| UNIVERSITY OF WARWICK | 3 | 0.104 |
| UNIVERSITY OF YORK UK | 3 | 0.104 |
| VA CONNECTICUT HEALTHCARE SYSTEM | 3 | 0.104 |
| VA SAN DIEGO HEALTHCARE SYSTEM | 3 | 0.104 |
| VALL D HEBRON INSTITUT DE RECERCA VHIR | 3 | 0.104 |
| WASHINGTON STATE UNIVERSITY | 3 | 0.104 |
| WENZHOU MEDICAL UNIVERSITY | 3 | 0.104 |
| WUHAN UNIVERSITY | 3 | 0.104 |
| YESHIVA UNIVERSITY | 3 | 0.104 |
| ZHENGZHOU UNIVERSITY | 3 | 0.104 |
| ADIS INTERNATIONAL | 2 | 0.069 |
| ADOLOR CORP | 2 | 0.069 |
| AICHI MEDICAL UNIVERSITY | 2 | 0.069 |
| ALBERTA CHILDRENS HOSPITAL | 2 | 0.069 |
| ALBERTA VET LABS | 2 | 0.069 |
| ALL INDIA INSTITUTE OF MEDICAL SCIENCES AIIMS NEW DELHI | 2 | 0.069 |
| AMER UNIV MIDDLE EAST | 2 | 0.069 |
| ANHUI UNIVERSITY OF CHINESE MEDICINE | 2 | 0.069 |
| ARISTOTLE UNIVERSITY OF THESSALONIKI | 2 | 0.069 |
| ARMY MEDICAL UNIVERSITY | 2 | 0.069 |
| ASIA UNIVERSITY TAIWAN | 2 | 0.069 |
| ASTON UNIVERSITY | 2 | 0.069 |
| ATLANTA VA HEALTH CARE SYSTEM | 2 | 0.069 |
| ATLANTA VA MEDICAL CENTER | 2 | 0.069 |
| AVIGEN INC | 2 | 0.069 |
| AZIENDA OSPEDALIERA SANT ANDREA | 2 | 0.069 |
| BAHAT HEALTH GROUP | 2 | 0.069 |
| BANGABANDHU SHEIKH MUJIB MED UNIV | 2 | 0.069 |
| BASKENT UNIVERSITY | 2 | 0.069 |
| BEIJING INST BRAIN DISORDERS | 2 | 0.069 |
| BENEMERITA UNIVERSIDAD AUTONOMA DE PUEBLA | 2 | 0.069 |
| BISPEBJERG FREDERIKSBERG HOSP | 2 | 0.069 |
| BROWN UNIVERSITY | 2 | 0.069 |
| CAIRO UNIVERSITY | 2 | 0.069 |
| CANGZHOU CENT HOSP | 2 | 0.069 |
| CAROLINAS MEDICAL CENTER | 2 | 0.069 |
| CATHOLIC KWANDONG UNIVERSITY | 2 | 0.069 |
| CEA | 2 | 0.069 |
| CHANGCHUN INSTITUTE OF APPLIED CHEMISTRY CAS | 2 | 0.069 |
| CHARITE MITTE CAMPUS | 2 | 0.069 |
| CHARLES UNIVERSITY PRAGUE | 2 | 0.069 |
| CHI MEI HOSPITAL | 2 | 0.069 |
| CHIBA CANCER CENTER | 2 | 0.069 |
| CHIESI PHARMACEUTICALS INC | 2 | 0.069 |
| CHILDREN S HOSPITAL COLORADO | 2 | 0.069 |
| CHINESE PEOPLES LIBERAT ARMY | 2 | 0.069 |
| CHU BREST | 2 | 0.069 |
| CHU DE MONTPELLIER | 2 | 0.069 |
| CHU DE NANTES | 2 | 0.069 |
| CHU STRASBOURG | 2 | 0.069 |
| CHU TOURS | 2 | 0.069 |
| CHUNG ANG UNIVERSITY | 2 | 0.069 |
| CINCINNATI CHILDREN S HOSPITAL MEDICAL CENTER | 2 | 0.069 |
| CLINIQUES UNIVERSITAIRES SAINT LUC | 2 | 0.069 |
| CMCC | 2 | 0.069 |
| COCHRANE COLLABORAT SECRETARIAT | 2 | 0.069 |
| COMMONWEALTH SCIENTIFIC INDUSTRIAL RESEARCH ORGANISATION CSIRO | 2 | 0.069 |
| COMPLEXO HOSPITALARIO UNIVERSITARIO DE SANTIAGO DE COMPOSTELA | 2 | 0.069 |
| COMPLUTENSE UNIVERSITY OF MADRID | 2 | 0.069 |
| COUNCIL OF SCIENTIFIC INDUSTRIAL RESEARCH CSIR INDIA | 2 | 0.069 |
| CSIC INSTITUTO DE PARASITOLOGIA Y BIOMEDICINA LOPEZ NEYRA IPBLN | 2 | 0.069 |
| CSIC UVA INSTITUTO DE BIOLOGIA Y GENETICA MOLECULAR IBGM | 2 | 0.069 |
| CTR CRANIOFACIAL RES DIAG | 2 | 0.069 |
| CTR HEMOSTASEOL TRANSFUS MED | 2 | 0.069 |
| DAIICHI COLL PHARMACEUT SCI | 2 | 0.069 |
| DEPT RADIOL | 2 | 0.069 |
| DEPT RHEUMATOL | 2 | 0.069 |
| DEUTSCHES RHEUMA FORSCHUNGSZENTRUM DRFZ | 2 | 0.069 |
| DEWEVER ZIEKENHUIS | 2 | 0.069 |
| DONGGUK UNIVERSITY | 2 | 0.069 |
| DR BC ROY COLL PHARM AHS | 2 | 0.069 |
| DURHAM VA MEDICAL CENTER | 2 | 0.069 |
| EASTSIDE WESTSIDE RES CTR | 2 | 0.069 |
| EPITECH GRP SRL | 2 | 0.069 |
| ESKISEHIR OSMANGAZI UNIVERSITY | 2 | 0.069 |
| FLANDERS INSTITUTE FOR BIOTECHNOLOGY | 2 | 0.069 |
| FREDERIKSBERG UNIV HOSP | 2 | 0.069 |
| GAZI UNIVERSITY | 2 | 0.069 |
| GEN HOSP SHEN YANG MIL REG | 2 | 0.069 |
| GEORGE INSTITUTE FOR GLOBAL HEALTH | 2 | 0.069 |
| GEORGE MASON UNIVERSITY | 2 | 0.069 |
| GREAT ORMOND STREET HOSPITAL FOR CHILDREN NHS FOUNDATION TRUST | 2 | 0.069 |
| GRIFFITH UNIVERSITY | 2 | 0.069 |
| GUANGZHOU UNIVERSITY OF CHINESE MEDICINE | 2 | 0.069 |
| GUNMA UNIVERSITY | 2 | 0.069 |
| GYEONGSANG NATIONAL UNIVERSITY | 2 | 0.069 |
| HANNOVER MEDICAL SCHOOL | 2 | 0.069 |
| HARBIN MEDICAL UNIVERSITY | 2 | 0.069 |
| HENRY FORD HOSPITAL | 2 | 0.069 |
| HERLEV GENTOFTE HOSP | 2 | 0.069 |
| HONG KONG UNIVERSITY OF SCIENCE TECHNOLOGY | 2 | 0.069 |
| HOPITAL UNIVERSITAIRE HOTEL DIEU APHP | 2 | 0.069 |
| HOSP CHINESE TRADIT MED LESHAN | 2 | 0.069 |
| HOSPITAL GENERAL DE MEXICO | 2 | 0.069 |
| HUNTER MEDICAL RESEARCH INSTITUTE | 2 | 0.069 |
| ICAR INDIAN VETERINARY RESEARCH INSTITUTE | 2 | 0.069 |
| IMAM ABDULRAHMAN BIN FAISAL UNIVERSITY | 2 | 0.069 |
| INDIAN COUNCIL OF AGRICULTURAL RESEARCH ICAR | 2 | 0.069 |
| INNLANDET HOSPITAL TRUST | 2 | 0.069 |
| INNOVAT STAT RES SRL | 2 | 0.069 |
| INSTITUT NATIONAL POLYTECHNIQUE DE TOULOUSE | 2 | 0.069 |
| INSTITUTE FOR WORK HEALTH | 2 | 0.069 |
| INSTITUTE OF BIOORGANIC CHEMISTRY OF THE RUSSIAN ACADEMY OF SCIENCES | 2 | 0.069 |
| INSTITUTE OF CANCER RESEARCH UK | 2 | 0.069 |
| INSTITUTE OF POST GRADUATE MEDICAL EDUCATION RESEARCH IPGMER | 2 | 0.069 |
| INSTITUTO MEXICANO DEL SEGURO SOCIAL | 2 | 0.069 |
| INSTITUTO NACIONAL DE CIENCIAS MEDICAS Y NUTRICION SALVADOR ZUBIRAN MEXICO | 2 | 0.069 |
| INTEGRATED TISSUE DYNAM LLC | 2 | 0.069 |
| INVALID FDN | 2 | 0.069 |
| IRCCS BONINO PULEJO | 2 | 0.069 |
| IRCCS CA GRANDA OSPEDALE MAGGIORE POLICLINICO | 2 | 0.069 |
| IRCCS FONDAZIONE SAN MATTEO | 2 | 0.069 |
| IRCCS ISTITUTO CLINICO HUMANITAS | 2 | 0.069 |
| IRCCS ISTITUTO NEUROLOGICO BESTA | 2 | 0.069 |
| IRCCS OSPEDALE SAN RAFFAELE | 2 | 0.069 |
| ISF COLLEGE OF PHARMACY | 2 | 0.069 |
| ISTANBUL HAYDARPASA NUMUNE TRAINING RESEARCH HOSPITAL | 2 | 0.069 |
| ISTANBUL HAYDARPASA SULTAN ABDULHAMID TRAINING RESEARCH HOSPITAL | 2 | 0.069 |
| ISTANBUL UNIVERSITY | 2 | 0.069 |
| JAMES J PETERS VA MEDICAL CENTER | 2 | 0.069 |
| JEONBUK NATIONAL UNIVERSITY | 2 | 0.069 |
| JIANGNAN UNIVERSITY | 2 | 0.069 |
| JIANGSU UNIVERSITY | 2 | 0.069 |
| JINZHOU MEDICAL UNIVERSITY | 2 | 0.069 |
| JOHNSON JOHNSON | 2 | 0.069 |
| JOHNSON JOHNSON USA | 2 | 0.069 |
| JORDAN UNIVERSITY OF SCIENCE TECHNOLOGY | 2 | 0.069 |
| JUNTENDO UNIVERSITY | 2 | 0.069 |
| KAHRAMANMARAS SUTCU IMAM UNIVERSITY | 2 | 0.069 |
| KANSAS STATE UNIVERSITY | 2 | 0.069 |
| KARADENIZ TEKNIK UNIVERSITY | 2 | 0.069 |
| KAZAN FEDERAL UNIVERSITY | 2 | 0.069 |
| KEIO UNIVERSITY | 2 | 0.069 |
| KERCKHOFF CLINIC | 2 | 0.069 |
| KINDAI UNIVERSITY KINKI UNIVERSITY | 2 | 0.069 |
| KING CHRISTIAN 10TH HOSP RHEUMAT DIS | 2 | 0.069 |
| KING S COLLEGE HOSPITAL | 2 | 0.069 |
| KING S COLLEGE HOSPITAL NHS FOUNDATION TRUST | 2 | 0.069 |
| KOREA UNIVERSITY MEDICAL CENTER KUMC | 2 | 0.069 |
| KREMBIL RESEARCH INSTITUTE | 2 | 0.069 |
| KRISTIANSTAD UNIVERSITY | 2 | 0.069 |
| KUMAMOTO HEALTH SCIENCE UNIVERSITY | 2 | 0.069 |
| KUNMING INSTITUTE OF ZOOLOGY | 2 | 0.069 |
| KUNMING MEDICAL UNIVERSITY | 2 | 0.069 |
| LA MADDALENA CANC CTR | 2 | 0.069 |
| LA PAIN CLIN | 2 | 0.069 |
| LEEDS BIOMEDICAL RESEARCH CENTRE | 2 | 0.069 |
| LI KA SHING KNOWLEDGE INSTITUTE | 2 | 0.069 |
| MAHIDOL UNIVERSITY | 2 | 0.069 |
| MANIPAL ACADEMY OF HIGHER EDUCATION MAHE | 2 | 0.069 |
| MARCHE POLYTECHNIC UNIVERSITY | 2 | 0.069 |
| MARTIN LUTHER UNIVERSITY HALLE WITTENBERG | 2 | 0.069 |
| MASSACHUSETTS EYE EAR INFIRMARY | 2 | 0.069 |
| MATSUMOTO DENTAL UNIVERSITY | 2 | 0.069 |
| MERSIN UNIVERSITY | 2 | 0.069 |
| MIL MED ACAD | 2 | 0.069 |
| MINIST EDUC | 2 | 0.069 |
| MINISTRY OF EDUCATION CHINA | 2 | 0.069 |
| MONASH INST PHARMACEUT SCI | 2 | 0.069 |
| MORE ROMSDAL HOSP TRUST | 2 | 0.069 |
| NANCHANG UNIVERSITY | 2 | 0.069 |
| NANYANG TECHNOLOGICAL UNIVERSITY | 2 | 0.069 |
| NANYANG TECHNOLOGICAL UNIVERSITY NATIONAL INSTITUTE OF EDUCATION NIE SINGAPORE | 2 | 0.069 |
| NATIONAL ACADEMY OF SCIENCES UKRAINE | 2 | 0.069 |
| NATIONAL AGEING RESEARCH INSTITUTE | 2 | 0.069 |
| NATIONAL CENTRAL UNIVERSITY | 2 | 0.069 |
| NATIONAL KAPODISTRIAN UNIVERSITY OF ATHENS | 2 | 0.069 |
| NATIONAL SUN YAT SEN UNIVERSITY | 2 | 0.069 |
| NATL ENGN CTR BIOCHIP SHANGHAI | 2 | 0.069 |
| NATL HLTH FAMILY PLANNING COMMISS | 2 | 0.069 |
| NATL INST OCCUPAT HLTH | 2 | 0.069 |
| NEW ENGLAND CTR HEADACHE | 2 | 0.069 |
| NIH NATIONAL INSTITUTE OF ARTHRITIS MUSCULOSKELETAL SKIN DISEASES NIAMS | 2 | 0.069 |
| NIH NATIONAL INSTITUTE OF BIOMEDICAL IMAGING BIOENGINEERING NIBIB | 2 | 0.069 |
| NIPPON MEDICAL SCHOOL | 2 | 0.069 |
| NIPPON SHINYAKU CO LTD | 2 | 0.069 |
| NORD INST CHIROPRACT CLIN BIOMECH | 2 | 0.069 |
| NORFOLK NORWICH UNIVERSITY HOSPITAL | 2 | 0.069 |
| NORFOLK NORWICH UNIVERSITY HOSPITALS NHS FOUNDATION TRUST | 2 | 0.069 |
| NORTH CAROLINA STATE UNIVERSITY | 2 | 0.069 |
| NORWEGIAN COMP CTR | 2 | 0.069 |
| OKAZAKI INSTITUTE FOR INTEGRATIVE BIOSCIENCE OIIB | 2 | 0.069 |
| OKLAHOMA MEDICAL RESEARCH FOUNDATION | 2 | 0.069 |
| OPHTHALMIC CONSULTANTS OF BOSTON | 2 | 0.069 |
| OREBRO UNIVERSITY | 2 | 0.069 |
| ORGANIX INC | 2 | 0.069 |
| OSAKA MEDICAL COLLEGE | 2 | 0.069 |
| OTTAWA HOSPITAL RESEARCH INSTITUTE | 2 | 0.069 |
| PARKER RES INST | 2 | 0.069 |
| PEKING UNION MEDICAL COLLEGE HOSPITAL | 2 | 0.069 |
| PENN STATE HEALTH | 2 | 0.069 |
| PHILIPPS UNIVERSITY MARBURG | 2 | 0.069 |
| PONTIFICIA UNIVERSIDADE CATOLICA DO RIO GRANDE DO SUL | 2 | 0.069 |
| POZNAN UNIVERSITY OF MEDICAL SCIENCES | 2 | 0.069 |
| PRIMARY CHILDRENS MED CTR | 2 | 0.069 |
| PURDUE PHARMA DISCOVERY RES | 2 | 0.069 |
| RAMBAM HEALTH CARE CAMPUS | 2 | 0.069 |
| REGENERON | 2 | 0.069 |
| ROCKEFELLER UNIVERSITY | 2 | 0.069 |
| ROYAL ADELAIDE HOSPITAL | 2 | 0.069 |
| ROYAL MELBOURNE INSTITUTE OF TECHNOLOGY RMIT | 2 | 0.069 |
| RURAL DEVELOPMENT ADMINISTRATION RDA | 2 | 0.069 |
| RWTH AACHEN UNIVERSITY | 2 | 0.069 |
| SANDOZ | 2 | 0.069 |
| SAPPORO MEDICAL UNIVERSITY | 2 | 0.069 |
| SCOTTISH AGRICULTURAL COLLEGE | 2 | 0.069 |
| SECHENOV FIRST MOSCOW STATE MEDICAL UNIVERSITY | 2 | 0.069 |
| SEE CLEARLY VIS GRP | 2 | 0.069 |
| SEMNAN UNIVERSITY OF MEDICAL SCIENCES | 2 | 0.069 |
| SHAHREKORD UNIVERSITY MEDICAL SCIENCES | 2 | 0.069 |
| SHANDONG UNIVERSITY OF TRADITIONAL CHINESE MEDICINE | 2 | 0.069 |
| SHANGHAI RES INST ACUPUNCTURE MERIDIAN | 2 | 0.069 |
| SHANGHAI UNIVERSITY | 2 | 0.069 |
| SHEFFIELD HALLAM UNIVERSITY | 2 | 0.069 |
| SHINSHU UNIVERSITY | 2 | 0.069 |
| SINGAPORE GENERAL HOSPITAL | 2 | 0.069 |
| SOUTH CENTRAL UNIVERSITY FOR NATIONALITIES | 2 | 0.069 |
| SOUTHERN MEDICAL UNIVERSITY CHINA | 2 | 0.069 |
| SOUTHWEST MEDICAL UNIVERSITY | 2 | 0.069 |
| SPINE CTR SOUTHERN DENMARK | 2 | 0.069 |
| ST VINCENT S HOSPITAL MELBOURNE | 2 | 0.069 |
| STARSHIP CHILDREN S HOSPITAL | 2 | 0.069 |
| SUNGKYUNKWAN UNIVERSITY SKKU | 2 | 0.069 |
| SWEDISH MEDICAL CENTER | 2 | 0.069 |
| TABRIZ UNIVERSITY OF MEDICAL SCIENCE | 2 | 0.069 |
| TAICHUNG VETERANS GENERAL HOSPITAL | 2 | 0.069 |
| TAISHO PHARMACEUT CO LTD | 2 | 0.069 |
| TAMPERE UNIVERSITY HOSPITAL | 2 | 0.069 |
| TECHNION ISRAEL INSTITUTE OF TECHNOLOGY | 2 | 0.069 |
| TEOFILO HERNANDO INST DRUG DISCOVERY | 2 | 0.069 |
| TEXAN EYE CARE | 2 | 0.069 |
| TEXAS A M HEALTH SCIENCE CENTER | 2 | 0.069 |
| THADWEIK ACAD MED | 2 | 0.069 |
| TOHO UNIVERSITY | 2 | 0.069 |
| TOHOKU MEDICAL PHARMACEUTICAL UNIVERSITY | 2 | 0.069 |
| TOKAI UNIVERSITY | 2 | 0.069 |
| TOKYO METROPOLITAN INSTITUTE FOR NEUROSCIENCE | 2 | 0.069 |
| TOKYO UNIVERSITY OF AGRICULTURE | 2 | 0.069 |
| TOKYO WOMEN S MEDICAL UNIVERSITY | 2 | 0.069 |
| TONGJI UNIVERSITY | 2 | 0.069 |
| TORANOMON HOSPITAL | 2 | 0.069 |
| TRI SERVICE GENERAL HOSPITAL | 2 | 0.069 |
| TRINITY COLLEGE DUBLIN | 2 | 0.069 |
| UMEA UNIVERSITY | 2 | 0.069 |
| UNILEVER | 2 | 0.069 |
| UNITED ARAB EMIRATES UNIVERSITY | 2 | 0.069 |
| UNITED STATES ARMY | 2 | 0.069 |
| UNIV HOSP COMPLEX GRANADA | 2 | 0.069 |
| UNIV HOSP CTR | 2 | 0.069 |
| UNIV HOSP KERRY | 2 | 0.069 |
| UNIVERSIDAD AUTONOMA DE TAMAULIPAS | 2 | 0.069 |
| UNIVERSIDAD AUTONOMA DEL ESTADO DE MORELOS | 2 | 0.069 |
| UNIVERSIDAD DE ALCALA | 2 | 0.069 |
| UNIVERSIDAD DE FRANCA | 2 | 0.069 |
| UNIVERSIDAD DE MALAGA | 2 | 0.069 |
| UNIVERSIDADE CIDADE DE SAO PAULO | 2 | 0.069 |
| UNIVERSIDADE DE LISBOA | 2 | 0.069 |
| UNIVERSIDADE DO SUL DE SANTA CATARINA | 2 | 0.069 |
| UNIVERSIDADE DO VALE DO ITAJAI | 2 | 0.069 |
| UNIVERSIDADE FEDERAL DA BAHIA | 2 | 0.069 |
| UNIVERSIDADE FEDERAL DO PIAUI | 2 | 0.069 |
| UNIVERSIDADE FEDERAL DO RIO GRANDE DO SUL | 2 | 0.069 |
| UNIVERSIDADE NOVE DE JULHO | 2 | 0.069 |
| UNIVERSIDADE TECNOLOGICA FEDERAL DO PARANA | 2 | 0.069 |
| UNIVERSITA DI MODENA E REGGIO EMILIA HOSPITAL | 2 | 0.069 |
| UNIVERSITE CONFEDERALE LEONARD DE VINCI | 2 | 0.069 |
| UNIVERSITE COTE D AZUR | 2 | 0.069 |
| UNIVERSITE DE BRETAGNE OCCIDENTALE | 2 | 0.069 |
| UNIVERSITE DE NANTES | 2 | 0.069 |
| UNIVERSITE DE VERSAILLES SAINT QUENTIN EN YVELINES | 2 | 0.069 |
| UNIVERSITE FEDERALE TOULOUSE MIDI PYRENEES COMUE | 2 | 0.069 |
| UNIVERSITE LIBRE DE BRUXELLES | 2 | 0.069 |
| UNIVERSITE PARIS EST CRETEIL VAL DE MARNE UPEC | 2 | 0.069 |
| UNIVERSITY HOSPITAL LEUVEN | 2 | 0.069 |
| UNIVERSITY OF BASEL | 2 | 0.069 |
| UNIVERSITY OF COLOGNE | 2 | 0.069 |
| UNIVERSITY OF COLORADO ANSCHUTZ MEDICAL CAMPUS | 2 | 0.069 |
| UNIVERSITY OF GENEVA | 2 | 0.069 |
| UNIVERSITY OF GENOA | 2 | 0.069 |
| UNIVERSITY OF GOTTINGEN | 2 | 0.069 |
| UNIVERSITY OF HAMBURG | 2 | 0.069 |
| UNIVERSITY OF HULL | 2 | 0.069 |
| UNIVERSITY OF LAUSANNE | 2 | 0.069 |
| UNIVERSITY OF LONDON SCHOOL OF PHARMACY | 2 | 0.069 |
| UNIVERSITY OF LOUISVILLE | 2 | 0.069 |
| UNIVERSITY OF MAINE SYSTEM | 2 | 0.069 |
| UNIVERSITY OF MARYLAND COLLEGE PARK | 2 | 0.069 |
| UNIVERSITY OF MASSACHUSETTS WORCESTER | 2 | 0.069 |
| UNIVERSITY OF NEW MEXICO | 2 | 0.069 |
| UNIVERSITY OF OCCUPATIONAL ENVIRONMENTAL HEALTH JAPAN | 2 | 0.069 |
| UNIVERSITY OF OKLAHOMA OKLAHOMA CITY | 2 | 0.069 |
| UNIVERSITY OF OVIEDO | 2 | 0.069 |
| UNIVERSITY OF PALERMO | 2 | 0.069 |
| UNIVERSITY OF SALERNO | 2 | 0.069 |
| UNIVERSITY OF SANTIAGO DE COMPOSTELA | 2 | 0.069 |
| UNIVERSITY OF SASKATCHEWAN | 2 | 0.069 |
| UNIVERSITY OF SPLIT | 2 | 0.069 |
| UNIVERSITY OF TARTU | 2 | 0.069 |
| UNIVERSITY OF TEHRAN | 2 | 0.069 |
| UNIVERSITY OF VERMONT | 2 | 0.069 |
| UNIVERSITY OF WESTERN AUSTRALIA | 2 | 0.069 |
| UNIVERSITY OF WISCONSIN MILWAUKEE | 2 | 0.069 |
| UNIVERSITY TORONTO MISSISSAUGA | 2 | 0.069 |
| URMIA UNIVERSITY | 2 | 0.069 |
| UROSPHERE | 2 | 0.069 |
| US FOOD DRUG ADMINISTRATION FDA | 2 | 0.069 |
| VA ANN ARBOR HEALTHCARE SYSTEM | 2 | 0.069 |
| VA GREATER LOS ANGELES HEALTHCARE SYSTEM | 2 | 0.069 |
| VENEZUELAN INSTITUTE SCIENCE RESEARCH | 2 | 0.069 |
| WALTER REED NATIONAL MILITARY MEDICAL CENTER | 2 | 0.069 |
| WALTON CENTRE | 2 | 0.069 |
| WEIFANG PEOPLES HOSP | 2 | 0.069 |
| WGKK HANUSCH HOSPITAL | 2 | 0.069 |
| WOMEN INFANTS HOSPITAL RHODE ISLAND | 2 | 0.069 |
| WONKWANG UNIVERSITY | 2 | 0.069 |
| XENON PHARMACEUT | 2 | 0.069 |
| XI AN UNIVERSITY OF ARCHITECTURE TECHNOLOGY | 2 | 0.069 |
| YONSEI UNIVERSITY | 2 | 0.069 |
| YUZUNCU YIL UNIVERSITY | 2 | 0.069 |
| 153TH HOSP PLA | 1 | 0.035 |
| 154TH CENT HOSP PLA | 1 | 0.035 |
| 17TH SHAHRIVAR HOSP | 1 | 0.035 |
| 232 COLL NURSING | 1 | 0.035 |
| 309 HOSP PLA | 1 | 0.035 |
| 94750 ARMY HOSP | 1 | 0.035 |
| 969TH HOSP PEOPLES LIBERAT ARMY CHINA | 1 | 0.035 |
| A DUPONT HOSP CHILDREN | 1 | 0.035 |
| A HEALTHTRACKRX CO | 1 | 0.035 |
| A MANZONI HOSP ASST LECCO | 1 | 0.035 |
| AALBORG HOSP | 1 | 0.035 |
| ABANT IZZET BAYSAL UNIVERSITY | 1 | 0.035 |
| ABBOTT | 1 | 0.035 |
| ABBOTT BIORES CTR | 1 | 0.035 |
| ABGENIX INC | 1 | 0.035 |
| ABO AKADEMI UNIVERSITY | 1 | 0.035 |
| ACADEMIA SINICA TAIWAN | 1 | 0.035 |
| ACADIA PHARMACEUT INC | 1 | 0.035 |
| ADAM GRUCA CLIN HOSP | 1 | 0.035 |
| ADMESCOPE LTD | 1 | 0.035 |
| ADV VIS CARE | 1 | 0.035 |
| ADYNXX INC | 1 | 0.035 |
| AECS MAARUTI COLL DENT SCI RES CTR | 1 | 0.035 |
| AGRICULTURE AGRI FOOD CANADA | 1 | 0.035 |
| AHEPA UNIVERSITY HOSPITAL | 1 | 0.035 |
| AI VIRTANEN INST | 1 | 0.035 |
| AICHI GAKUIN UNIVERSITY | 1 | 0.035 |
| AIR FORCE GENERAL HOSPITAL PLA | 1 | 0.035 |
| AIR LIQUIDE | 1 | 0.035 |
| AIREDALE NHS FDN TRUST | 1 | 0.035 |
| AJINOMOTO CO INC | 1 | 0.035 |
| AKITA UNIVERSITY | 1 | 0.035 |
| AKRON CHILDREN S HOSPITAL | 1 | 0.035 |
| AL AZHAR UNIVERSITY | 1 | 0.035 |
| AL RAFIDAIN UNIV COLL | 1 | 0.035 |
| ALAN EDWARDS CTR RES PAIN | 1 | 0.035 |
| ALBERT EINSTEIN COLLEGE OF MEDICINE | 1 | 0.035 |
| ALBERT LUDWINGS UNIV FREIBURG | 1 | 0.035 |
| ALBERTA AGR FORESTRY | 1 | 0.035 |
| ALBERTA AGR RURAL DEV | 1 | 0.035 |
| ALBERTA HEALTH SERVICES AHS | 1 | 0.035 |
| ALBERTA HERITAGE FDN MED RES | 1 | 0.035 |
| ALBERTA HORSE IND ASSOC | 1 | 0.035 |
| ALEXANDRA HOSPITAL | 1 | 0.035 |
| ALEXANDRE BONJEAN MED SURG CTR | 1 | 0.035 |
| ALEXIANER KRANKENHAUS | 1 | 0.035 |
| ALFRIED KRUPP HOSPITAL | 1 | 0.035 |
| ALL INDIA SHRI SHIVAJI MEM SOC COLL PHARM | 1 | 0.035 |
| ALLERGAN | 1 | 0.035 |
| ALLIED HLTH CARE CTR RHEUMATOL REHABIL GRONINGE | 1 | 0.035 |
| ALTA RIC SVILUPPO BIOTECNOL SRLU | 1 | 0.035 |
| ALTRIA CLIENT SERV INC | 1 | 0.035 |
| AMBIOTIS | 1 | 0.035 |
| AMER ACAD ORTHOPAED SURG | 1 | 0.035 |
| AMER BRITISH COWDRY MED CTR | 1 | 0.035 |
| AMER COLL RHEUMATOL | 1 | 0.035 |
| AMICI ITALIA ONLUS | 1 | 0.035 |
| AMSTERDAM UMC | 1 | 0.035 |
| ANAGIN INC | 1 | 0.035 |
| ANESTHESIOL | 1 | 0.035 |
| ANFOMED GMBH | 1 | 0.035 |
| ANGES MG INC | 1 | 0.035 |
| ANIM SURG CLIN SEATTLE | 1 | 0.035 |
| ANKARA ATATURK TRAINING RESEARCH HOSPITAL | 1 | 0.035 |
| ANKARA NUMUNE TRAINING RESEARCH HOSPITAL | 1 | 0.035 |
| ANPHAR ROLLAND LABS | 1 | 0.035 |
| ANZAC RESEARCH INSTITUTE | 1 | 0.035 |
| AOU POLICLINICO S ORSOLA MALPIGHI | 1 | 0.035 |
| APPALACHIAN REG ORTHOPAED SPORTS MED | 1 | 0.035 |
| ARCISPED S MARIA NUOVA | 1 | 0.035 |
| ARCISPEDALE SANT ANNA | 1 | 0.035 |
| AREEO | 1 | 0.035 |
| ARISTOTLE UNIV THESSALONIKI | 1 | 0.035 |
| ARIZONA STATE UNIVERSITY | 1 | 0.035 |
| ARMED POLICE TIANJIN CORPS HOSP | 1 | 0.035 |
| ARRAY BIOPHARMA | 1 | 0.035 |
| ARTHRIT RES UK CTR ADOLESCENT RHEUMATOL | 1 | 0.035 |
| ASAHI KASEI PHARMA CO LTD | 1 | 0.035 |
| ASAHI KASEI PHARMA CORP | 1 | 0.035 |
| ASIAN INST GASTROENTEROL | 1 | 0.035 |
| ASKAZENICA R D MONKREAL | 1 | 0.035 |
| ASKLEPIOS KLINIKUM | 1 | 0.035 |
| ASL CN2 | 1 | 0.035 |
| ASSIUT UNIVERSITY | 1 | 0.035 |
| ASTRAEA THERAPEUT LLC | 1 | 0.035 |
| ASUBIO PHARMA CO | 1 | 0.035 |
| ATATURK UNIVERSITY | 1 | 0.035 |
| ATHENS MEDICAL SCHOOL | 1 | 0.035 |
| ATLANTIC DIAGNOST LABS | 1 | 0.035 |
| ATLANTIC HEALTH SYSTEM | 1 | 0.035 |
| ATLANTIS EYE CARE | 1 | 0.035 |
| AUBURN UNIVERSITY | 1 | 0.035 |
| AUBURN UNIVERSITY SYSTEM | 1 | 0.035 |
| AUCKLAND UNIVERSITY OF TECHNOLOGY | 1 | 0.035 |
| AUGUSTA BIOMED RES CORP | 1 | 0.035 |
| AUSL REGGIO EMILIA | 1 | 0.035 |
| AUSTRALIAN NATIONAL UNIVERSITY | 1 | 0.035 |
| AUSTRALIAN YOUNG UROL RESEARCHERS ORG YURO | 1 | 0.035 |
| AUSTRIAN ACADEMY OF SCIENCES | 1 | 0.035 |
| AVIGEN | 1 | 0.035 |
| AZ GROENINGE HOSP | 1 | 0.035 |
| AZIENDA OSPED OSPED CIRCOLO | 1 | 0.035 |
| AZIENDA OSPED RILIEVO NAZL ALTA SPECIALIZZAZ GA | 1 | 0.035 |
| AZIENDA OSPEDALIERA DI COSENZA | 1 | 0.035 |
| AZIENDA OSPEDALIERA SAN CAMILLO FORLANINI | 1 | 0.035 |
| AZIENDA OSPEDALIERA UNIVERSITARIA INTEGRATA VERONA | 1 | 0.035 |
| AZIENDA SANIT LOCALE BIELLA | 1 | 0.035 |
| BAITURSYNOV KOSTANAY STATE UNIVERSITY | 1 | 0.035 |
| BANASTHALI VIDYAPITH | 1 | 0.035 |
| BAODING 2 CENT HOSP | 1 | 0.035 |
| BAPTIST MEM GOLDEN TRIANGLE HOSP | 1 | 0.035 |
| BARCELONA INSTITUTE OF SCIENCE TECHNOLOGY | 1 | 0.035 |
| BARNSLEY HOSP NHS FDN TRUST | 1 | 0.035 |
| BARTS HEALTH NHS TRUST | 1 | 0.035 |
| BAUSCH LOMB | 1 | 0.035 |
| BAUSCH LOMB INC | 1 | 0.035 |
| BAXTER INTERNATIONAL INC | 1 | 0.035 |
| BAY PINES VA HEALTHCARE SYST | 1 | 0.035 |
| BAYER AG | 1 | 0.035 |
| BAYER HEALTHCARE PHARMACEUTICALS | 1 | 0.035 |
| BAYSTATE MEDICAL CENTER | 1 | 0.035 |
| BBSRC BABRAHAM INSTITUTE | 1 | 0.035 |
| BC ROY COLL PHARM ALLIED HLTH SCI | 1 | 0.035 |
| BCN PEPTIDES | 1 | 0.035 |
| BEATSON INSTITUTE | 1 | 0.035 |
| BEAUMONT HEALTH | 1 | 0.035 |
| BEIJING SHUILI HOSP | 1 | 0.035 |
| BEIJING UNIV TECHNOL | 1 | 0.035 |
| BEIJING UNIVERSITY OF CHEMICAL TECHNOLOGY | 1 | 0.035 |
| BEIRUT ARAB UNIVERSITY | 1 | 0.035 |
| BELFAST CITY HOSPITAL | 1 | 0.035 |
| BELFORT MONTBELIARD HOSP | 1 | 0.035 |
| BENI SUEF UNIVERSITY | 1 | 0.035 |
| BEST DENT SCI COLL HOSP | 1 | 0.035 |
| BHARATI VIDYAPEETH DEEMED UNIVERSITY | 1 | 0.035 |
| BINZHOU MEDICAL UNIVERSITY | 1 | 0.035 |
| BIOFORE INC | 1 | 0.035 |
| BIOIBERICA | 1 | 0.035 |
| BIONORICA SE | 1 | 0.035 |
| BIOSTAT CONSULTANTS INC | 1 | 0.035 |
| BIOSTATEM | 1 | 0.035 |
| BIOTEKNA BIOMED TECHNOL | 1 | 0.035 |
| BIOXELL | 1 | 0.035 |
| BIRMINGHAM VET ADM HOSP | 1 | 0.035 |
| BIRUNI UNIVERSITY | 1 | 0.035 |
| BIZEN KASEI CHEM CO LTD | 1 | 0.035 |
| BLUESTONE CTR CLIN RES | 1 | 0.035 |
| BNAI ZION MEDICAL CENTER | 1 | 0.035 |
| BOLDER BIOPATH INC | 1 | 0.035 |
| BORDEAUX UNIV | 1 | 0.035 |
| BOSE INSTITUTE | 1 | 0.035 |
| BOSTON COLLEGE | 1 | 0.035 |
| BOW VALLEY RES | 1 | 0.035 |
| BOWDOIN COLLEGE | 1 | 0.035 |
| BRAINS ON LINE BV | 1 | 0.035 |
| BRANDEIS UNIVERSITY | 1 | 0.035 |
| BRAZILIAN AGR RES AGCY EMBRAPA | 1 | 0.035 |
| BRIGHTON AND SUSSEX UNIVERSITY HOSPITALS NHS TRUST | 1 | 0.035 |
| BRISTOL ROYAL INFIRMARY | 1 | 0.035 |
| BROCK NIAGARA CTR HLTH WELL BEING | 1 | 0.035 |
| BROCK UNIVERSITY | 1 | 0.035 |
| BRUNEL UNIVERSITY | 1 | 0.035 |
| BUCAK MH ORDU UNIV EGITIM ARASTIRMA HASTANESI | 1 | 0.035 |
| BUDAI IRGALMASRENDI KORHAZ | 1 | 0.035 |
| BUDDHIST TZU CHI MED FDN | 1 | 0.035 |
| BULGARIAN ACADEMY OF SCIENCES | 1 | 0.035 |
| BURST | 1 | 0.035 |
| BURTON HOSP NHS FDN TRUST | 1 | 0.035 |
| CA FONCELLOS HOSP | 1 | 0.035 |
| CALIF EYE INST | 1 | 0.035 |
| CALIFORNIA STATE UNIVERSITY SYSTEM | 1 | 0.035 |
| CAMBRIDGE UNIV NHS FDN TRUST | 1 | 0.035 |
| CANTERBURY INNOVAT CTR | 1 | 0.035 |
| CANTONAL HOSP BADEN | 1 | 0.035 |
| CAPITAL INSTITUTE OF PEDIATRICS CIP | 1 | 0.035 |
| CATALAN INSTITUTE OF NANOSCIENCE NANOTECHNOLOGY ICN2 | 1 | 0.035 |
| CATARACT GLAUCOMA CTR | 1 | 0.035 |
| CATHAY GENERAL HOSPITAL | 1 | 0.035 |
| CATHOLIC UNIVERSITY OF DAEGU | 1 | 0.035 |
| CEINGE BIOTECNOLOGIE AVANZATE | 1 | 0.035 |
| CELIAC DIS CTR | 1 | 0.035 |
| CENT HOSP HLTH GRP | 1 | 0.035 |
| CENT HOSP KRISTIANSTAD | 1 | 0.035 |
| CENT MED MALOU | 1 | 0.035 |
| CENTRAL FINLAND CENTRAL HOSPITAL | 1 | 0.035 |
| CENTRAL INSTITUTE OF MENTAL HEALTH | 1 | 0.035 |
| CENTRAL UNIVERSITY OF RAJASTHAN CURAJ | 1 | 0.035 |
| CENTRE HOSPITALIER SAINTE ANNE | 1 | 0.035 |
| CENTRE HOSPITALIER UNIVERSITAIRE D ANGERS | 1 | 0.035 |
| CENTRE HOSPITALIER UNIVERSITAIRE VAUDOIS CHUV | 1 | 0.035 |
| CENTRE LEON BERARD | 1 | 0.035 |
| CENTRO HOSPITALAR DE LISBOA OCIDENTAL EPE | 1 | 0.035 |
| CEREP | 1 | 0.035 |
| CHANG BING SHOW CHWAN MEMORIAL HOSPITAL | 1 | 0.035 |
| CHANG GUNG UNIV SCI TECHNOL | 1 | 0.035 |
| CHANG GUNG UNIVERSITY OF SCIENCE TECHNOLOGY | 1 | 0.035 |
| CHANG JUNG CHRISTIAN UNIVERSITY | 1 | 0.035 |
| CHANGHUA CHRISTIAN HOSPITAL | 1 | 0.035 |
| CHARLIE NORWOOD VA MED CTR | 1 | 0.035 |
| CHENG HSIN GENERAL HOSPITAL | 1 | 0.035 |
| CHENGDU LILAI BIOTECHNOL CO LTD | 1 | 0.035 |
| CHENGDU SPORT UNIVERSITY | 1 | 0.035 |
| CHIA NAN UNIVERSITY OF PHARMACY SCIENCE | 1 | 0.035 |
| CHIA YI CHRISTIAN HOSPITAL | 1 | 0.035 |
| CHIBA ROSAI HOSP | 1 | 0.035 |
| CHIBANISHI GEN HOSP | 1 | 0.035 |
| CHICAGO CORNEA CONSULTANTS | 1 | 0.035 |
| CHICAGO MEDICAL SCHOOL | 1 | 0.035 |
| CHILDREN S HOSPITAL LOS ANGELES | 1 | 0.035 |
| CHILDREN S MERCY HOSPITAL | 1 | 0.035 |
| CHILDRENS HOSP | 1 | 0.035 |
| CHILDRENS HOSP SREBRNJAK | 1 | 0.035 |
| CHILDRENS HOSP TIANJIN | 1 | 0.035 |
| CHILDRENS HOSP UPMC | 1 | 0.035 |
| CHILDRENS NUTR RES CTR | 1 | 0.035 |
| CHINA THREE GORGES UNIVERSITY | 1 | 0.035 |
| CHINESE ACADEMY OF AGRICULTURAL SCIENCES | 1 | 0.035 |
| CHINESE PEOPLE LIBERAT ARMY GEN HOSP | 1 | 0.035 |
| CHINOOK CONTRACT RES | 1 | 0.035 |
| CHONNAM NATIONAL UNIVERSITY HOSPITAL | 1 | 0.035 |
| CHRISTCHURCH HOSPITAL NEW ZEALAND | 1 | 0.035 |
| CHU LIMOGES | 1 | 0.035 |
| CHU NICE | 1 | 0.035 |
| CHU PURPAN | 1 | 0.035 |
| CHUNG ANG UNIVERSITY HOSPITAL | 1 | 0.035 |
| CHUNG HO MEMORIAL HOSPITAL | 1 | 0.035 |
| CHUNGBUK NATIONAL UNIVERSITY | 1 | 0.035 |
| CHUNGKANG COLL CULTURAL IND | 1 | 0.035 |
| CHUNGNAM NATIONAL UNIVERSITY HOSPITAL | 1 | 0.035 |
| CIBERBBN | 1 | 0.035 |
| CIBERES | 1 | 0.035 |
| CINCINNATI EYE INST | 1 | 0.035 |
| CIUDAD UNIV | 1 | 0.035 |
| CLEARSIDE BIOMED | 1 | 0.035 |
| CLERMONT UNIV | 1 | 0.035 |
| CLEVELAND CHIROPRACT COLL | 1 | 0.035 |
| CLEVELAND EYE CLIN | 1 | 0.035 |
| CLIENIA SCHLOSSLI AG | 1 | 0.035 |
| CLIN ARTHRIT RHEUMATISM | 1 | 0.035 |
| CLIN BARMELWEID | 1 | 0.035 |
| CLIN CTR VOJVODINA | 1 | 0.035 |
| CLIN DATA SERV INC | 1 | 0.035 |
| CLIN HORSES KIRCHHEIM | 1 | 0.035 |
| CLIN HOSP CTR DR DRAGISA MISOVIC DEDINJE | 1 | 0.035 |
| CLIN ST ANNA | 1 | 0.035 |
| CLINDATA SERV INC | 1 | 0.035 |
| CLINICAL CENTRE OF SERBIA | 1 | 0.035 |
| CNSBIO LTD PTY LTD | 1 | 0.035 |
| COCHRANE CENTRE | 1 | 0.035 |
| COGITARS GMBH IN LIQ | 1 | 0.035 |
| COGITARS GMBH LIQ | 1 | 0.035 |
| COLL NURSING | 1 | 0.035 |
| COLL PHARM | 1 | 0.035 |
| COLORADO NEUROL INST | 1 | 0.035 |
| COMPREHENS EYE CARE LTD | 1 | 0.035 |
| COMPREHENS PAIN REHABIL | 1 | 0.035 |
| COMSATS UNIVERSITY ISLAMABAD CUI | 1 | 0.035 |
| CONCORD REPATRIATION GENERAL HOSPITAL | 1 | 0.035 |
| CONEM SCI SECRETARY | 1 | 0.035 |
| CONNER GLAUCOMA CTR | 1 | 0.035 |
| CONSIGLIO PER LA RICERCA IN AGRICOLTURA E L ANALISI DELL ECONOMIA AGRARIA CREA | 1 | 0.035 |
| COPENHAGEN CTR ARTHRIT RES COPECARE | 1 | 0.035 |
| COPENHAGEN UNIV HOSP BISPEBJERG FREDERIKSBERG | 1 | 0.035 |
| COPERNICUS HOSP GDANSK | 1 | 0.035 |
| COPPERLINE SOLUT LLC | 1 | 0.035 |
| CORNEA CONSULTANTS | 1 | 0.035 |
| CORPORAL MICHAEL J CRESCENZ VA MED CTR | 1 | 0.035 |
| COUNCIL NUTR ENVIRONM MED CONEM | 1 | 0.035 |
| COVANCE | 1 | 0.035 |
| CRAIGAVON AREA HOSP | 1 | 0.035 |
| CRANBERRY TOWNSHIP EYE CARE SPECIALISTS | 1 | 0.035 |
| CREIGHTON UNIVERSITY | 1 | 0.035 |
| CRIB | 1 | 0.035 |
| CROSSHOUSE HOSP | 1 | 0.035 |
| CROYDON UNIVERSITY HOSPITAL | 1 | 0.035 |
| CSIC CENTRO DE BIOLOGIA MOLECULAR SEVERO OCHOA CBM | 1 | 0.035 |
| CSIC INSTITUTO CAJAL IC | 1 | 0.035 |
| CSIC INSTITUTO DE QUIMICA AVANZADA DE CATALUNA IQAC | 1 | 0.035 |
| CSIR INDIAN INSTITUTE OF CHEMICAL BIOLOGY IICB | 1 | 0.035 |
| CSIR INDIAN INSTITUTE OF CHEMICAL TECHNOLOGY IICT | 1 | 0.035 |
| CTR DIAGNOST IMAGING | 1 | 0.035 |
| CTR DRUG RES DEV | 1 | 0.035 |
| CTR EDUC RES AGEING | 1 | 0.035 |
| CTR EVALUAT MED | 1 | 0.035 |
| CTR EXCELLENCE EYE CARE | 1 | 0.035 |
| CTR INOVACAO ENSAIOS PRECLIN CIENP | 1 | 0.035 |
| CTR INVEST MEMBRANE EXCITABIL DIS | 1 | 0.035 |
| CTR LIFE SCI | 1 | 0.035 |
| CTR OFTALM METROPOLITANO | 1 | 0.035 |
| CTR RECH DEV PIERRE FABRE | 1 | 0.035 |
| CTR RECH PIERRE FABRE | 1 | 0.035 |
| CTR REPROD KRIZEVCI | 1 | 0.035 |
| CTR RHEUMAT DIS | 1 | 0.035 |
| CTR RHEUMATOL SPINE DIS | 1 | 0.035 |
| CTR SCI INVECCHIAMENTO MED TRASLAZ CESI MET | 1 | 0.035 |
| CTR UNIV NEWTON PAIVA | 1 | 0.035 |
| CTR VET MED | 1 | 0.035 |
| CUHK SHENZHEN RES INST | 1 | 0.035 |
| CUKUROVA UNIVERSITY | 1 | 0.035 |
| CURTIN UNIVERSITY | 1 | 0.035 |
| CYCLOLAB LTD | 1 | 0.035 |
| CYPRUS LEAGUE RHEUMATISM | 1 | 0.035 |
| CYTOKINE PHARMASCI INC | 1 | 0.035 |
| CYTOS BIOTECHNOL AG | 1 | 0.035 |
| CYTOTHERAPEUT INC | 1 | 0.035 |
| CZECH ACADEMY OF SCIENCES | 1 | 0.035 |
| D WESTERN THERAPEUT INST | 1 | 0.035 |
| DAEGU GYEONGBUK MED INNOVAT FDN | 1 | 0.035 |
| DAEJEON UNIVERSITY | 1 | 0.035 |
| DAIICHI SANKYO COMPANY LIMITED | 1 | 0.035 |
| DAINIPPON SUMITOMO PHARMACEUTICAL COMPANY | 1 | 0.035 |
| DANA FARBER CANCER INSTITUTE | 1 | 0.035 |
| DANDERYDS HOSPITAL | 1 | 0.035 |
| DANISH HOSP RHEUMAT DIS | 1 | 0.035 |
| DATA INC | 1 | 0.035 |
| DE MONTFORT UNIVERSITY | 1 | 0.035 |
| DEF VET CTR INTEGRAT PAIN MANAGEMENT | 1 | 0.035 |
| DEFENCE INSTITUTE OF PHYSIOLOGY ALLIED SCIENCES DIPAS | 1 | 0.035 |
| DEFENCE RESEARCH DEVELOPMENT ORGANISATION DRDO | 1 | 0.035 |
| DELFT UNIVERSITY OF TECHNOLOGY | 1 | 0.035 |
| DEPARTMENT OF SCIENCE TECHNOLOGY INDIA | 1 | 0.035 |
| DEPT ANESTHESIA CRIT CARE PAIN MED | 1 | 0.035 |
| DEPT ANESTHESIOL PAIN MED | 1 | 0.035 |
| DEPT CLIN EXPT MED | 1 | 0.035 |
| DEPT COMMUNITY HLTH SCI | 1 | 0.035 |
| DEPT HLTH SCI V LE EUROPA | 1 | 0.035 |
| DEPT HUMAN MORPHOL | 1 | 0.035 |
| DEPT MICROBIOL IMMUNOL | 1 | 0.035 |
| DEPT NEUROL | 1 | 0.035 |
| DEPT NEUROSURG | 1 | 0.035 |
| DEPT PEDIAT | 1 | 0.035 |
| DEPT PHARM PHARMACEUT TECHNOL PARASITOL | 1 | 0.035 |
| DEPT PSYCHIAT | 1 | 0.035 |
| DEPT RHEUMATOL REHABIL | 1 | 0.035 |
| DEPT VET MICROBIOL | 1 | 0.035 |
| DESCARTES UNIV | 1 | 0.035 |
| DESERT MED ADV | 1 | 0.035 |
| DHAKA INT UNIV | 1 | 0.035 |
| DIABET EYE MED CLIN | 1 | 0.035 |
| DIAGNOST IMAGING CTR | 1 | 0.035 |
| DIAMOND HEADACHE CLIN LTD | 1 | 0.035 |
| DICLE UNIVERSITY | 1 | 0.035 |
| DISCOVER VIS CTR | 1 | 0.035 |
| DIST HOSP | 1 | 0.035 |
| DIV EXPT NEURORADIOL | 1 | 0.035 |
| DIV GEN INTERNAL MED | 1 | 0.035 |
| DIV MED PSYCHOL | 1 | 0.035 |
| DIVERDRUGS | 1 | 0.035 |
| DIVERDRUGS SL | 1 | 0.035 |
| DOGWOOD PHARMACEUT | 1 | 0.035 |
| DOUGLAS INST MENTAL HLTH | 1 | 0.035 |
| DOUGLAS MENTAL HLTH UNIV INST | 1 | 0.035 |
| DOWNE HOSP | 1 | 0.035 |
| DR ENRIQUE GARCES HOSP | 1 | 0.035 |
| DR JORG SCHNITKER GMBH | 1 | 0.035 |
| DR PINCHAS BORENSTEIN TALPIOT MED LEADERSHIP PROG | 1 | 0.035 |
| DR SIRAJUL ISLAM MED COLL | 1 | 0.035 |
| DRUG CONSULTING NETWORK | 1 | 0.035 |
| DRUG DISCOVERY NETWORK ZURICH | 1 | 0.035 |
| DSM NV | 1 | 0.035 |
| DSO NATL LABS | 1 | 0.035 |
| DUDLEY GRP HOSP NHS FDN TRUST | 1 | 0.035 |
| DUKE NUS MED SCH | 1 | 0.035 |
| DUZCE UNIVERSITY | 1 | 0.035 |
| EAST CAROLINA UNIVERSITY | 1 | 0.035 |
| EAST CHINA NORMAL UNIVERSITY | 1 | 0.035 |
| EASTERN OKLAHOMA ORTHOPED CTR | 1 | 0.035 |
| EBRI | 1 | 0.035 |
| EC JRC ISPRA SITE | 1 | 0.035 |
| ECOLE NATIONALE DE LA STATISTIQUE ET DE L ANALYSE DE L INFORMATION ENSAI | 1 | 0.035 |
| ECOLE POLYTECHNIQUE FEDERALE DE LAUSANNE | 1 | 0.035 |
| ECOLE SUPERIEURE DE PHYSIQUE ET DE CHIMIE INDUSTRIELLES DE LA VILLE DE PARIS ESPCI | 1 | 0.035 |
| EDWARD VIA VIRGINIA COLL OSTEOPATH MED | 1 | 0.035 |
| EGAS MONIZ HOSPITAL | 1 | 0.035 |
| EICOSIS LLC | 1 | 0.035 |
| EISAI CO LTD | 1 | 0.035 |
| EMERGENCY CARE UNIT | 1 | 0.035 |
| EMGO INST HLTH CARE RES EMGO | 1 | 0.035 |
| EN CHU KONG HOSP | 1 | 0.035 |
| ENVISIA THERAPEUT | 1 | 0.035 |
| ENVIVO PHARMACEUT | 1 | 0.035 |
| EQUUS RES | 1 | 0.035 |
| ERNEST GALLO CLINIC RESEARCH CENTER | 1 | 0.035 |
| ESTONIAN UNIVERSITY OF LIFE SCIENCES | 1 | 0.035 |
| ETAT ETHOL APPL | 1 | 0.035 |
| EULAR SOCIAL LEAGUES PATIENTS REPRESENTAT | 1 | 0.035 |
| EULJI UNIVERSITY | 1 | 0.035 |
| EURO AMER NUTRACEUT INC | 1 | 0.035 |
| EUROFINS OPTIMED | 1 | 0.035 |
| EURON EUROPEAN GRAD SCH NEUROSCI | 1 | 0.035 |
| EUROPEAN COMMISSION JOINT RESEARCH CENTRE | 1 | 0.035 |
| EVIDENCE AID | 1 | 0.035 |
| EYE ASSOCIATES VINELAND | 1 | 0.035 |
| EYE CARE SPECIALISTS | 1 | 0.035 |
| EYE CTR RACINE KENOSHA | 1 | 0.035 |
| EYESIGHT ASSOCIATES | 1 | 0.035 |
| EZHOU WOMEN CHILDREN HLTH HOSP | 1 | 0.035 |
| FAC ARTS SCI | 1 | 0.035 |
| FAC AVANTIS | 1 | 0.035 |
| FAC ENFERMAGEM NOVA ESPERANCA FACENE | 1 | 0.035 |
| FAC MED | 1 | 0.035 |
| FAC MED PORTO | 1 | 0.035 |
| FAC MED RIBEIRAO PRETO | 1 | 0.035 |
| FACULDADE SAO LEOPOLDO MANDIC | 1 | 0.035 |
| FAIRFAX HOSP CHILDREN | 1 | 0.035 |
| FAKEEH COLL MED SCI | 1 | 0.035 |
| FAMENITY CO LTD | 1 | 0.035 |
| FAMILY HLTH INT | 1 | 0.035 |
| FAR EASTERN MEMORIAL HOSPITAL | 1 | 0.035 |
| FDN ALCORCON | 1 | 0.035 |
| FDN DR PEDRO M CATOGGIO PROGRESO REUMATOL | 1 | 0.035 |
| FDN RITA LEVI MONTALCINI | 1 | 0.035 |
| FEPAR | 1 | 0.035 |
| FICHTE ENDL ELMER EYECARE | 1 | 0.035 |
| FIFTH HOSP HARBIN | 1 | 0.035 |
| FIFTH HOSP PLA | 1 | 0.035 |
| FIFTH MEDICAL CENTER OF CHINESE PLA GENERAL HOSPITAL | 1 | 0.035 |
| FIFTH PEOPLES HOSP QINGDAO | 1 | 0.035 |
| FIMABIS | 1 | 0.035 |
| FIRST HOSP JIAXING | 1 | 0.035 |
| FIRST HOSP SHIJIAZHUANG | 1 | 0.035 |
| FIRST PEOPLES HOSP CHANGZHOU | 1 | 0.035 |
| FIRST PEOPLES HOSP HUAIAN | 1 | 0.035 |
| FLAGSHIP PIONEERING | 1 | 0.035 |
| FLINDERS MEDICAL CENTRE | 1 | 0.035 |
| FLORIDA ORTHOPAEDIC INSTITUTE | 1 | 0.035 |
| FLORIDA STATE UNIVERSITY | 1 | 0.035 |
| FMUP | 1 | 0.035 |
| FONDAZIONE CENCI BOLOGNETTI | 1 | 0.035 |
| FOOYIN UNIVERSITY | 1 | 0.035 |
| FRED HUTCHINSON CANCER CENTER | 1 | 0.035 |
| FRENCH SOC RHEUMATOL | 1 | 0.035 |
| FRIEDRICH MIESCHER INSTITUTE FOR BIOMEDICAL RESEARCH | 1 | 0.035 |
| FT WORTH TEXAN EYE | 1 | 0.035 |
| FUJIAN ACAD TRADIT CHINESE MED | 1 | 0.035 |
| FUJIAN NORMAL UNIVERSITY | 1 | 0.035 |
| FUJINO ORTHOPAED CLIN | 1 | 0.035 |
| FUJIREBIO INC | 1 | 0.035 |
| FUJISAWA PHARMACEUTICAL CO LTD | 1 | 0.035 |
| FUKUOKA CLIN | 1 | 0.035 |
| FUKUSHIMA MEDICAL UNIVERSITY | 1 | 0.035 |
| FUNDACAO EZEQUIEL DIAS | 1 | 0.035 |
| FUNDACAO EZEQUIEL DIAS FIRED | 1 | 0.035 |
| FUNDACIO SANT JOAN DE DEU | 1 | 0.035 |
| FUTURE UNIVERSITY FUE | 1 | 0.035 |
| G HATZIKOSTA GEN HOSP | 1 | 0.035 |
| GABRIELE ANNUNZIO UNIV FDN | 1 | 0.035 |
| GADJAH MADA UNIVERSITY | 1 | 0.035 |
| GAETANO RUMMO HOSP | 1 | 0.035 |
| GALDERMA R D SNC | 1 | 0.035 |
| GANSU HLTH VOCAT COLL | 1 | 0.035 |
| GANSU PROV HOSP | 1 | 0.035 |
| GANZHOU PEOPLES HOSP | 1 | 0.035 |
| GARTNAVEL ROYAL HOSPITAL | 1 | 0.035 |
| GAZIANTEP UNIVERSITY | 1 | 0.035 |
| GAZILER PHYSICAL THERAPY RESEARCH HOSPITAL | 1 | 0.035 |
| GDANSK UNIVERSITY OF PHYSICAL EDUCATION SPORT | 1 | 0.035 |
| GEDEON RICHTER CHEMISTRY WORKS | 1 | 0.035 |
| GEISINGER COMMONWEALTH SCH MED | 1 | 0.035 |
| GEISINGER COMMONWEALTH SCHOOL OF MEDICINE | 1 | 0.035 |
| GEN HOSP 58 | 1 | 0.035 |
| GEN HOSP MEXICO | 1 | 0.035 |
| GEN HOSP PEOPLES LIBERAT ARMY | 1 | 0.035 |
| GENENCELL CO LTD | 1 | 0.035 |
| GENENTECH | 1 | 0.035 |
| GENERAL UNIVERSITY HOSPITAL OF ALICANTE | 1 | 0.035 |
| GENTOFTE HOSPITAL | 1 | 0.035 |
| GEORGE EBY RES | 1 | 0.035 |
| GEORGE FAY YEE CTR HEALTHCARE INNOVAT | 1 | 0.035 |
| GEORGE WASHINGTON UNIVERSITY | 1 | 0.035 |
| GEORGIA INSTITUTE OF TECHNOLOGY | 1 | 0.035 |
| GERMAN CANCER RESEARCH CENTER DKFZ | 1 | 0.035 |
| GIFU UNIVERSITY | 1 | 0.035 |
| GILL CTR BIOMOL SCI | 1 | 0.035 |
| GIRONA UNIVERSITY HOSPITAL DR JOSEP TRUETA | 1 | 0.035 |
| GLOBALACORN LTD | 1 | 0.035 |
| GORYEB CHILDRENS HOSP ATLANTIC HLTH | 1 | 0.035 |
| GRAD SCH MED | 1 | 0.035 |
| GRADUATE UNIVERSITY FOR ADVANCED STUDIES JAPAN | 1 | 0.035 |
| GREEN LIFE CTR RHEUMAT CARE RES | 1 | 0.035 |
| GREENWICH HOSP | 1 | 0.035 |
| GRIGORE T POPA UNIVERSITY OF MEDICINE PHARMACY | 1 | 0.035 |
| GROENE HART ZIEKENHUIS | 1 | 0.035 |
| GUANGDONG MEDICAL UNIVERSITY | 1 | 0.035 |
| GUANGXI ZHUANG AUTONOMOUS REG MATERNAL CHILD HL | 1 | 0.035 |
| GUANGZHOU MIL COMMAND CHINESE PLA | 1 | 0.035 |
| GUIZHOU MEDICAL UNIVERSITY | 1 | 0.035 |
| GUIZHOU PROV PEOPLES HOSP | 1 | 0.035 |
| GULHANE TRAINING RESEARCH HOSPITAL | 1 | 0.035 |
| GV SONNY MONTGOMERY VET ADM MED CTR | 1 | 0.035 |
| H SAN RAFFAELE RESNATI | 1 | 0.035 |
| HAEMEK MED CTR | 1 | 0.035 |
| HAMAD GENERAL HOSPITAL | 1 | 0.035 |
| HAMAD HOSP | 1 | 0.035 |
| HAMILTON GEN HOSP | 1 | 0.035 |
| HANGZHOU MEDICAL COLLEGE | 1 | 0.035 |
| HANYANG UNIVERSITY | 1 | 0.035 |
| HARBIN RED CROSS CENT HOSP | 1 | 0.035 |
| HARTFORD HOSPITAL | 1 | 0.035 |
| HARVARD CHILDRENS HOSP | 1 | 0.035 |
| HARVARD MED SCH | 1 | 0.035 |
| HARVARD SCHOOL OF DENTAL MEDICINE | 1 | 0.035 |
| HARVARD T H CHAN SCHOOL OF PUBLIC HEALTH | 1 | 0.035 |
| HASSELT UNIVERSITY | 1 | 0.035 |
| HAWAII PHYS THERAPY INC | 1 | 0.035 |
| HAYWOOD HOSP | 1 | 0.035 |
| HCPA | 1 | 0.035 |
| HEADACHE CTR RIO | 1 | 0.035 |
| HEINRICH HEINE UNIVERSITY DUSSELDORF HOSPITAL | 1 | 0.035 |
| HELIOS HOSP GRP | 1 | 0.035 |
| HELIOS KLINIKEN | 1 | 0.035 |
| HELSINN SA | 1 | 0.035 |
| HENAN UNIVERSITY | 1 | 0.035 |
| HENAN UNIVERSITY OF SCIENCE TECHNOLOGY | 1 | 0.035 |
| HERIOT WATT UNIVERSITY | 1 | 0.035 |
| HGU GRAGORIO MARANON HOSP | 1 | 0.035 |
| HIETZING HOSPITAL | 1 | 0.035 |
| HIGH INST RES EDUC TRANSFUS MED | 1 | 0.035 |
| HIROSAKI UNIVERSITY | 1 | 0.035 |
| HJORRING HOSP | 1 | 0.035 |
| HLTH SCI CTR | 1 | 0.035 |
| HOFSTRA UNIVERSITY | 1 | 0.035 |
| HONG KONG BAPTIST UNIVERSITY | 1 | 0.035 |
| HONG KONG POLYTECHNIC UNIVERSITY | 1 | 0.035 |
| HOPITAL UNIVERSITAIRE BICHAT CLAUDE BERNARD APHP | 1 | 0.035 |
| HOPITAL UNIVERSITAIRE BRETONNEAU APHP | 1 | 0.035 |
| HOPITAL UNIVERSITAIRE NECKER ENFANTS MALADES APHP | 1 | 0.035 |
| HOPITAL UNIVERSITAIRE ROBERT DEBRE APHP | 1 | 0.035 |
| HOPITAL UNIVERSITAIRE ROTHSCHILD APHP | 1 | 0.035 |
| HOPITAL UNIVERSITAIRE SAINT LOUIS APHP | 1 | 0.035 |
| HOPITAL UNIVERSITAIRE TENON APHP | 1 | 0.035 |
| HORSE IND ASSOC ALBERTA | 1 | 0.035 |
| HOSP ARNSBERG | 1 | 0.035 |
| HOSP CLIN PORTO ALEGRE | 1 | 0.035 |
| HOSP ESPECIALIDADES CTR MED LA RAZA | 1 | 0.035 |
| HOSP FDN CLIN MED SUR | 1 | 0.035 |
| HOSP FED SERVIDORES ESTADO | 1 | 0.035 |
| HOSP GEN MEXICO EDUARDO LICEAGA | 1 | 0.035 |
| HOSP HUNAN PROV TUMOR HOSP | 1 | 0.035 |
| HOSP RAJA PERMAISURI BAINUN | 1 | 0.035 |
| HOSP SAN JAIME | 1 | 0.035 |
| HOSP SANTA RITA CASSIA | 1 | 0.035 |
| HOSP SAO RAFAEL | 1 | 0.035 |
| HOSP SIRIO LIBANES | 1 | 0.035 |
| HOSP STOMATOL | 1 | 0.035 |
| HOSP UNIV ARNAU DE VILANOVA LLEIDA | 1 | 0.035 |
| HOSP UNIV LA MORALEJA | 1 | 0.035 |
| HOSP UNIV QUIRON | 1 | 0.035 |
| HOSPITAL CARLOS HAYA | 1 | 0.035 |
| HOSPITAL CLINICO SAN CARLOS | 1 | 0.035 |
| HOSPITAL DE LA PRINCESA | 1 | 0.035 |
| HOSPITAL GERMANS TRIAS I PUJOL | 1 | 0.035 |
| HOSPITAL ITALIANO DE BUENOS AIRES | 1 | 0.035 |
| HOSPITAL PUERTA DE HIERRO MAJADAHONDA | 1 | 0.035 |
| HOSPITAL SANTA MARIA | 1 | 0.035 |
| HOSPITAL SIRIO LIBANES | 1 | 0.035 |
| HOSPITAL UNIVERSITARIO CRUCES | 1 | 0.035 |
| HOSPITAL UNIVERSITARIO PUERTA DEL MAR | 1 | 0.035 |
| HOSPITAL UNIVERSITARIO RAMON Y CAJAL | 1 | 0.035 |
| HOUSTON EYE ASSOCIATES | 1 | 0.035 |
| HOWARD UNIVERSITY | 1 | 0.035 |
| HUANGPU DIST HOSP INTEGRATED TRADIT CHINESE WES | 1 | 0.035 |
| HUANGPU HOSP INTEGRATED TRADIT CHINESE WESTERN | 1 | 0.035 |
| HUAZHONG AGRICULTURAL UNIVERSITY | 1 | 0.035 |
| HUBEI MINZU UNIVERSITY | 1 | 0.035 |
| HUBEI UNIVERSITY OF MEDICINE | 1 | 0.035 |
| HULL EYE CTR | 1 | 0.035 |
| HUMANITAS CLIN RES CTR | 1 | 0.035 |
| HUNAN NORMAL UNIVERSITY | 1 | 0.035 |
| HUNAN PROV TUMOR HOSP | 1 | 0.035 |
| HUNAN UNIVERSITY OF CHINESE MEDICINE | 1 | 0.035 |
| HUNTER PAIN CLIN | 1 | 0.035 |
| HUNTINGTON MEDICAL RESEARCH INSTITUTES | 1 | 0.035 |
| I3 DATA SERV | 1 | 0.035 |
| IAMEL | 1 | 0.035 |
| IMMANUEL HOSP | 1 | 0.035 |
| IMPACT BIOTECH | 1 | 0.035 |
| INCOZEN THERAPEUT LTD | 1 | 0.035 |
| INCYTE | 1 | 0.035 |
| INDENA | 1 | 0.035 |
| INDIRA GANDHI NATIONAL TRIBAL UNIVERSITY | 1 | 0.035 |
| INER | 1 | 0.035 |
| INFINITUS CHINA CO LTD | 1 | 0.035 |
| INFLAMEX | 1 | 0.035 |
| INHA UNIVERSITY | 1 | 0.035 |
| INJE UNIVERSITY | 1 | 0.035 |
| INLAND NORWAY UNIVERSITY OF APPLIED SCIENCES | 1 | 0.035 |
| INNER MONGOLIA MEDICAL UNIVERSITY | 1 | 0.035 |
| INNOVET ITALIA SRL | 1 | 0.035 |
| INOVA FAIRFAX HOSPITAL | 1 | 0.035 |
| INOVA HEALTH SYSTEM | 1 | 0.035 |
| INST APPL STAT LTD | 1 | 0.035 |
| INST BRAIN SCI | 1 | 0.035 |
| INST CLIN RES | 1 | 0.035 |
| INST EXPT KLIN PHARMAKOL TOXIKOL | 1 | 0.035 |
| INST FINLAY | 1 | 0.035 |
| INST GENOM FONCT | 1 | 0.035 |
| INST GREEN BIO SCI TECHNOL | 1 | 0.035 |
| INST INVEST BIOMED ST PAU | 1 | 0.035 |
| INST INVEST SANITARIA LA PRINCESA | 1 | 0.035 |
| INST MED SCI | 1 | 0.035 |
| INST NACL ENFERMEDADES RESP | 1 | 0.035 |
| INST NACL ENFERMEDADES RESP ISMAEL COSIO VILLEGAS | 1 | 0.035 |
| INST NACL PSIQUIATRIA RAMON DE LA FUENTE MUNIZ | 1 | 0.035 |
| INST NEUROL | 1 | 0.035 |
| INST PARNASSE DEUX ALICE | 1 | 0.035 |
| INST PHARM TECHNOL | 1 | 0.035 |
| INST PHYSIOL BALNEOL | 1 | 0.035 |
| INST UNIV FERNANDEZ VEGA | 1 | 0.035 |
| INST VENEZOLANO INVEST CIENT | 1 | 0.035 |
| INSTITUT D INVESTIGACIO BIOMEDICA DE BELLVITGE IDIBELL | 1 | 0.035 |
| INSTITUT DE RECERCA BIOMEDICA IRB LLEIDA | 1 | 0.035 |
| INSTITUT PAOLI CALMETTE IPC | 1 | 0.035 |
| INSTITUTE OF BIOLOGY EXPERIMENTAL MEDICINE | 1 | 0.035 |
| INSTITUTE OF GENERAL PATHOLOGY PATHOPHYSIOLOGY | 1 | 0.035 |
| INSTITUTE OF GENETICS ANIMAL BREEDING POLISH ACADEMY OF SCIENCES | 1 | 0.035 |
| INSTITUTE OF MOLECULAR BIOTECHNOLOGY IMBA | 1 | 0.035 |
| INSTITUTE OF ORGANIC CHEMISTRY AND BIOCHEMISTRY OF THE CZECH ACADEMY OF SCIENCES | 1 | 0.035 |
| INSTITUTE OF ZOOLOGY CAS | 1 | 0.035 |
| INSTITUTO DE ONCOLOGIA DE ASTURIAS | 1 | 0.035 |
| INSTITUTO NACIONAL DE SALUD PUBLICA | 1 | 0.035 |
| INT CLIN RES INST | 1 | 0.035 |
| INT CTR MOL PHYSIOL | 1 | 0.035 |
| INTERNATIONAL CENTRE FOR DIARRHOEAL DISEASE RESEARCH | 1 | 0.035 |
| INTERNATIONAL MEDICAL UNIVERSITY MALAYSIA | 1 | 0.035 |
| INTERNATIONAL SCHOOL FOR ADVANCED STUDIES | 1 | 0.035 |
| INTERTEK PHARMACEUT SERV | 1 | 0.035 |
| INTERVET INT BV | 1 | 0.035 |
| IOWA STATE UNIVERSITY | 1 | 0.035 |
| IPSEN | 1 | 0.035 |
| IRAN UNIVERSITY OF MEDICAL SCIENCES | 1 | 0.035 |
| IRANIAN SOCIAL SECUR ORG | 1 | 0.035 |
| IRCCS ARCISPEDALE S MARIA NUOVA | 1 | 0.035 |
| IRCCS BAMBINO GESU | 1 | 0.035 |
| IRCCS BURLO GAROFOLO | 1 | 0.035 |
| IRCCS FONDAZIONE SALVATORE MAUGERI | 1 | 0.035 |
| IRCCS ISTITUTO ORTOPEDICO GALEAZZI | 1 | 0.035 |
| IRCCS ISTITUTO ORTOPEDICO RIZZOLI | 1 | 0.035 |
| IRCCS MELDOLA IRST | 1 | 0.035 |
| IRCCS OASI MARIA SS | 1 | 0.035 |
| IRTA | 1 | 0.035 |
| ISAL FDN | 1 | 0.035 |
| ISDIN | 1 | 0.035 |
| ISIS PHARMACEUTICALS INC | 1 | 0.035 |
| IST FARMACEUT CANDIOLI | 1 | 0.035 |
| IST ORTOPED G PINI | 1 | 0.035 |
| IST SANTA MARGHERITA | 1 | 0.035 |
| ISTANBUL MEDIPOL UNIVERSITY | 1 | 0.035 |
| ISTITUTO DI BIOSTRUTTURE E BIOIMMAGINI IBB CNR | 1 | 0.035 |
| ISTITUTO DI CHIMICA BIOMOLECOLARE ICB CNR | 1 | 0.035 |
| ISTITUTO DI CIBERNETICA EDUARDO CAIANIELLO ICIB CNR | 1 | 0.035 |
| ISTITUTO SUPERIORE DI SANITA | 1 | 0.035 |
| ITALIAN PAIN GRP | 1 | 0.035 |
| IVIC CBB | 1 | 0.035 |
| JACKSON LABORATORY | 1 | 0.035 |
| JAGIELLONIAN UNIVERSITY | 1 | 0.035 |
| JAMES CANCER HOSPITAL SOLOVE RESEARCH INSTITUTE | 1 | 0.035 |
| JAMES COOK UNIVERSITY | 1 | 0.035 |
| JAMES WHITCOMB RILEY HOSPITAL CHILDREN | 1 | 0.035 |
| JANMANGAL HOMEOPATHY WELLNESS CTR | 1 | 0.035 |
| JANSSEN BIOTECH INC | 1 | 0.035 |
| JANSSEN CILAG | 1 | 0.035 |
| JANSSEN INC | 1 | 0.035 |
| JAPAN TOBACCO INC | 1 | 0.035 |
| JEJU NATIONAL UNIVERSITY | 1 | 0.035 |
| JEOLLANAMDO INST NAT RESOURCES RES JINR | 1 | 0.035 |
| JIANGHAN UNIVERSITY | 1 | 0.035 |
| JIANGSU KANION PHARMACEUT CO LTD | 1 | 0.035 |
| JIANGSU OCEAN UNIVERSITY | 1 | 0.035 |
| JIANGSU PROV INST CHINESE MED | 1 | 0.035 |
| JIANGSU PROV TRADIT CHINESE MED HOSP | 1 | 0.035 |
| JIANGXI UNIVERSITY OF FINANCE ECONOMICS | 1 | 0.035 |
| JIANGXI UNIVERSITY OF TRADITIONAL CHINESE MEDICINE | 1 | 0.035 |
| JIAXING UNIVERSITY | 1 | 0.035 |
| JICHI MEDICAL UNIVERSITY | 1 | 0.035 |
| JILIN MEDICAL UNIVERSITY | 1 | 0.035 |
| JINAN UNIVERSITY | 1 | 0.035 |
| JINING 2 PEOPLES HOSP | 1 | 0.035 |
| JINING FIRST PEOPLES HOSP | 1 | 0.035 |
| JINING MEDICAL UNIVERSITY | 1 | 0.035 |
| JONES EYE CLIN | 1 | 0.035 |
| JORVI HOSP | 1 | 0.035 |
| JOSA ANDRAS HOSP | 1 | 0.035 |
| KAMAGAYA GEN HOSP | 1 | 0.035 |
| KANAZAWA MEDICAL UNIVERSITY | 1 | 0.035 |
| KANAZAWA UNIVERSITY | 1 | 0.035 |
| KANGWON NATIONAL UNIVERSITY | 1 | 0.035 |
| KANSAS UNIV MED BIOSCI | 1 | 0.035 |
| KANTONSSPITAL BASELLAND | 1 | 0.035 |
| KANTONSSPITAL ST GALLEN | 1 | 0.035 |
| KAOHSIUNG MUNICIPAL CHINESE MED HOSP | 1 | 0.035 |
| KARAKORAM INTERNATIONAL UNIVERSITY | 1 | 0.035 |
| KASETSART UNIVERSITY | 1 | 0.035 |
| KAT HOSP | 1 | 0.035 |
| KAWASAKI MEDICAL SCHOOL | 1 | 0.035 |
| KESSLER INSTITUTE FOR REHABILITATION | 1 | 0.035 |
| KEY LAB DRUG TARGET RES PHARMACODYNAM EVALUAT H | 1 | 0.035 |
| KEY LAB NEUROGENET CHANNELOPATHIES GUANGDONG PR | 1 | 0.035 |
| KHON KAEN UNIVERSITY | 1 | 0.035 |
| KIIT UNIVERSITY | 1 | 0.035 |
| KINDERSPITAL | 1 | 0.035 |
| KINEMED INC | 1 | 0.035 |
| KING CHRISTIAN X HOSP RHEUMAT DIS | 1 | 0.035 |
| KING GEORGE HOSPITAL | 1 | 0.035 |
| KING GEORGE S MEDICAL UNIVERSITY | 1 | 0.035 |
| KING GEORGE V MEM HOSP | 1 | 0.035 |
| KING KHALID UNIVERSITY | 1 | 0.035 |
| KISSEI PHARMACEUT CO LTD | 1 | 0.035 |
| KITASATO INST | 1 | 0.035 |
| KITASATO JR COLL HLTH HYG SCI | 1 | 0.035 |
| KLINIKEN LANDKREISES | 1 | 0.035 |
| KLINIKUM GARM PARTENKIRCHEN | 1 | 0.035 |
| KLINIKUM HOF | 1 | 0.035 |
| KNEE PRESERVAT CARTILAGE RESTORAT CTR | 1 | 0.035 |
| KOBE GAKUIN UNIVERSITY | 1 | 0.035 |
| KOREA FOOD DRUG ADM | 1 | 0.035 |
| KOREA FOREST RESEARCH INSTITUTE KFRI | 1 | 0.035 |
| KOREA HLTH SUPPLEMENTS INST KHSI | 1 | 0.035 |
| KOREA RESEARCH INSTITUTE OF BIOSCIENCE BIOTECHNOLOGY KRIBB | 1 | 0.035 |
| KSU UNIV | 1 | 0.035 |
| KT G RES INST | 1 | 0.035 |
| KUALA LUMPUR SPORT MED CTR | 1 | 0.035 |
| KUOPIO UNIVERSITY HOSPITAL | 1 | 0.035 |
| KURDISTAN UNIVERSITY OF MEDICAL SCIENCES | 1 | 0.035 |
| KURUME UNIVERSITY | 1 | 0.035 |
| KUWAIT UNIVERSITY | 1 | 0.035 |
| KWS BIOTEST | 1 | 0.035 |
| KYOLAB LTDA | 1 | 0.035 |
| KYONGGI UNIVERSITY | 1 | 0.035 |
| KYORIN PHARMACEUTICAL CO LTD | 1 | 0.035 |
| KYOWA HAKKO KIRIN CO LTD | 1 | 0.035 |
| KYUSHU DENTAL UNIVERSITY | 1 | 0.035 |
| LAB RHUMATOL APPL | 1 | 0.035 |
| LAB UPSA BMS | 1 | 0.035 |
| LABEX ION CHANNEL SCI THERAPEUT | 1 | 0.035 |
| LABEX MEDALIS | 1 | 0.035 |
| LAGOS STATE UNIVERSITY | 1 | 0.035 |
| LAINZ HOSP | 1 | 0.035 |
| LAMAR UNIVERSITY | 1 | 0.035 |
| LANDESKRANKENHAUS GRIMMENSTEIN HOCHEGG | 1 | 0.035 |
| LANSSJUKHUSET RYHOV | 1 | 0.035 |
| LAUREL EYE CLIN | 1 | 0.035 |
| LAURENTIUS HOSP | 1 | 0.035 |
| LAURENTIUS HOSP ROERMOND | 1 | 0.035 |
| LAY LINE GENOM | 1 | 0.035 |
| LEBANESE INT UNIV | 1 | 0.035 |
| LEBANESE UNIVERSITY | 1 | 0.035 |
| LEEDS BECKETT UNIVERSITY | 1 | 0.035 |
| LEEDS COMMUNITY HEALTHCARE MUSCULOSKELETAL REHA | 1 | 0.035 |
| LEHMAN COLLEGE CUNY | 1 | 0.035 |
| LEHMANN EYE CTR | 1 | 0.035 |
| LEIDEN GENET UNIV | 1 | 0.035 |
| LEIGHTON HOSP | 1 | 0.035 |
| LEITER NO ZENTRUMS RHEUMATOL | 1 | 0.035 |
| LES CORP INC | 1 | 0.035 |
| LEVENSON EYE ASSOCIATES | 1 | 0.035 |
| LEXINGTON VA MEDICAL CENTER | 1 | 0.035 |
| LIAOCHENG FOURTH PEOPLES HOSP | 1 | 0.035 |
| LIAOCHENG PEOPLES HOSP | 1 | 0.035 |
| LIFESPAN HEALTH RHODE ISLAND | 1 | 0.035 |
| LIFETREE RES | 1 | 0.035 |
| LINYI PEOPLES HOSP | 1 | 0.035 |
| LIPHOOK EQUINE HOSP | 1 | 0.035 |
| LIT BIOPHARMA CO LTD | 1 | 0.035 |
| LITHUANIAN UNIVERSITY OF HEALTH SCIENCES | 1 | 0.035 |
| LOHOCLA RES CORPORAT | 1 | 0.035 |
| LONDON DEANERY | 1 | 0.035 |
| LONDON HEALTH SCIENCES CENTRE | 1 | 0.035 |
| LORESTAN UNIVERSITY OF MEDICAL SCIENCES | 1 | 0.035 |
| LOUIS A WEISS MEM HOSP | 1 | 0.035 |
| LOUISIANA STATE UNIVERSITY HEALTH SCIENCES CENTER AT SHREVEPORT | 1 | 0.035 |
| LOVISENBERG DIAKONAL HOSP | 1 | 0.035 |
| LUIGI SACCO HOSPITAL | 1 | 0.035 |
| LUNDBECK CTR FAST TRACK HIP KNEE ARTHROPLASTY | 1 | 0.035 |
| LUOHE MED COLL | 1 | 0.035 |
| MACKAY JUNIOR COLLEGE OF MEDICINE NURSING MANAGEMENT | 1 | 0.035 |
| MADDALENA CLIN | 1 | 0.035 |
| MAGEE WOMENS HOSP | 1 | 0.035 |
| MAGEE WOMENS RESEARCH INSTITUTE | 1 | 0.035 |
| MANSOURA UNIVERSITY | 1 | 0.035 |
| MARBURG UNIV | 1 | 0.035 |
| MARKUS HOSP | 1 | 0.035 |
| MARSHALL UNIVERSITY | 1 | 0.035 |
| MARTEL EYE MED GRP | 1 | 0.035 |
| MASARYK UNIVERSITY BRNO | 1 | 0.035 |
| MASSACHUSETTS DEPARTMENT OF PUBLIC HEALTH | 1 | 0.035 |
| MASSACHUSETTS EYE RES SURG INST | 1 | 0.035 |
| MASSACHUSETTS GEN HOSP CHILDREN | 1 | 0.035 |
| MASSEY UNIVERSITY | 1 | 0.035 |
| MATRIX LABS LTD | 1 | 0.035 |
| MAX GRUNDIG KLIN | 1 | 0.035 |
| MCLAIN MED ASSOCIATES | 1 | 0.035 |
| MED COLL OHIO | 1 | 0.035 |
| MED UNIV | 1 | 0.035 |
| MEDICAL CENTER OF ALKMAAR | 1 | 0.035 |
| MEDICAL SPECTRUM TWENTE | 1 | 0.035 |
| MEDICAL UNIVERSITY OF BIALYSTOK | 1 | 0.035 |
| MEDICAL UNIVERSITY OF GRAZ | 1 | 0.035 |
| MEDICAL UNIVERSITY SOFIA | 1 | 0.035 |
| MEDIVIR | 1 | 0.035 |
| MEDTRONIC | 1 | 0.035 |
| MEIJI UNIVERSITY | 1 | 0.035 |
| MEITETSU HOSP | 1 | 0.035 |
| MEM HERMANN GREATER HTS HOSP | 1 | 0.035 |
| MEMORIAL HEALTHCARE GROUP | 1 | 0.035 |
| MEMORIAL SLOAN KETTERING CANCER CENTER | 1 | 0.035 |
| MENNINGER DEPT PSYCHIAT BEHAV SCI | 1 | 0.035 |
| MERCK FROSST CANADA INC | 1 | 0.035 |
| MERCY HLTH RES RYAN HEADACHE CTR | 1 | 0.035 |
| MERCY SPECIALIST CTR | 1 | 0.035 |
| MERIAL LTD | 1 | 0.035 |
| METAPHORE PHARMACEUT | 1 | 0.035 |
| MEULAN LES MUREAUX HOSP CTR | 1 | 0.035 |
| MICHIGAN STATE UNIVERSITY COLLEGE OF HUMAN MEDICINE | 1 | 0.035 |
| MIDLAND CHIROPRACT CLIN | 1 | 0.035 |
| MIDLANDS PARTNERSHIP FDN TRUST | 1 | 0.035 |
| MIDWEST EYE INST | 1 | 0.035 |
| MIDWESTERN UNIVERSITY | 1 | 0.035 |
| MIDWESTERN UNIVERSITY CHICAGO COLLEGE OF OSTEOPATHIC MEDICINE | 1 | 0.035 |
| MIL HOSP PECS | 1 | 0.035 |
| MINIST PUBL HLTH | 1 | 0.035 |
| MINISTRY OF HEALTH TURKEY | 1 | 0.035 |
| MINNESOTA EYE CONSULTANTS | 1 | 0.035 |
| MOHAMMED VI UNIV HLTH SCI UM6SS | 1 | 0.035 |
| MOLOGEN AG | 1 | 0.035 |
| MONROE WOODBURY HIGH SCH | 1 | 0.035 |
| MONSANTO | 1 | 0.035 |
| MONSELICE HOSP | 1 | 0.035 |
| MORPHOSYS | 1 | 0.035 |
| MOUNT ELIZABETH MEDICAL CENTRE | 1 | 0.035 |
| MSD TAIWAN | 1 | 0.035 |
| MT ALVERNIA HOSP | 1 | 0.035 |
| MT ELIZABETH HOSP | 1 | 0.035 |
| MTA DE NEUROSCI RES GRP | 1 | 0.035 |
| MTA SZTE NEUROSCI RES GRP | 1 | 0.035 |
| MULTI RADIANCE MED | 1 | 0.035 |
| MULTIUSER HLTH CTR FACIL CMULTISEDIDE | 1 | 0.035 |
| MUNDIPHARMA PHARMACEUT SLC | 1 | 0.035 |
| MURDOCH CHILDREN S RESEARCH INSTITUTE | 1 | 0.035 |
| MUSASHINO UNIV | 1 | 0.035 |
| MUSKOGEE BONE JOINT SPORTS MED CLIN | 1 | 0.035 |
| MYRIAD GENET | 1 | 0.035 |
| N RAMA VARIER AYURVEDA FDN | 1 | 0.035 |
| NA4ING UNIV CHINESE MED | 1 | 0.035 |
| NAGASAKI UNIVERSITY | 1 | 0.035 |
| NAMIK KEMAL UNIVERSITY | 1 | 0.035 |
| NANJING MEISHAN HOSP | 1 | 0.035 |
| NANJING SHUIXIMEN HOSP | 1 | 0.035 |
| NANKAI UNIVERSITY | 1 | 0.035 |
| NARA INSTITUTE OF SCIENCE TECHNOLOGY | 1 | 0.035 |
| NATIONAL CANCER CENTER JAPAN | 1 | 0.035 |
| NATIONAL CENTER FOR NEUROLOGY PSYCHIATRY JAPAN | 1 | 0.035 |
| NATIONAL HEALTH RESEARCH INSTITUTES TAIWAN | 1 | 0.035 |
| NATIONAL INSTITUTE OF TECHNOLOGY AGARTALA | 1 | 0.035 |
| NATIONAL RESEARCH INSTITUTE OF CHINESE MEDICINE | 1 | 0.035 |
| NATIONAL TAIPEI UNIVERSITY | 1 | 0.035 |
| NATIONAL TAIWAN NORMAL UNIVERSITY | 1 | 0.035 |
| NATL CLIN RES CTR GERIATR DISORDERS | 1 | 0.035 |
| NATL COLL NAT MED | 1 | 0.035 |
| NATL HOSP ORG DISASTER MED CTR | 1 | 0.035 |
| NATL INST DRUG ABUSE IRP | 1 | 0.035 |
| NATL INST GENET ENGN BIOTECHNOL | 1 | 0.035 |
| NATL INST HLTH RES LEEDS BIOMED RES CTR | 1 | 0.035 |
| NATL INST PUBL HLTH | 1 | 0.035 |
| NATL INST RHEUMAT DIS | 1 | 0.035 |
| NATL REHABIL CTR PERSONS DISABIL | 1 | 0.035 |
| NATL REHABIL HOSP | 1 | 0.035 |
| NATL TAITUNG UNIV | 1 | 0.035 |
| NATL WOMENS HLTH | 1 | 0.035 |
| NATL YANG MING UNIV HOSP | 1 | 0.035 |
| NAVAL GEN HOSP | 1 | 0.035 |
| NEDSPINE | 1 | 0.035 |
| NEMA RES INC | 1 | 0.035 |
| NETAJI SUBHAS MAHAVIDYALAYA | 1 | 0.035 |
| NETHERLANDS ORGANIZATION APPLIED SCIENCE RESEARCH | 1 | 0.035 |
| NETHERY EYE ASSOCIATES | 1 | 0.035 |
| NEURALSTEM INC | 1 | 0.035 |
| NEUROADJUVANTS INC | 1 | 0.035 |
| NEUROCHLORE | 1 | 0.035 |
| NEUROCRINE BIOSCIENCES | 1 | 0.035 |
| NEUROGEN CORP | 1 | 0.035 |
| NEUROL INST C BESTA IRCCS FDN | 1 | 0.035 |
| NEUROMODULAT SPECIALISTS LLC | 1 | 0.035 |
| NEUROSCI CTR ZURICH | 1 | 0.035 |
| NEUROSCIENCE RESEARCH AUSTRALIA | 1 | 0.035 |
| NEUROSOLUT LTD | 1 | 0.035 |
| NEVADA SYSTEM OF HIGHER EDUCATION NSHE | 1 | 0.035 |
| NEW CROSS HOSPITAL | 1 | 0.035 |
| NEW ENGLAND BAPTIST HOSPITAL | 1 | 0.035 |
| NEW JEDDAH CLIN HOSP | 1 | 0.035 |
| NEW JERSEY INSTITUTE OF TECHNOLOGY | 1 | 0.035 |
| NEW PHARMACEUT SPA | 1 | 0.035 |
| NEW YORK MEDICAL COLLEGE | 1 | 0.035 |
| NEW YORK PRESBYTERIAN SPINE HOSP | 1 | 0.035 |
| NEW YORK STATE PSYCHIATRY INSTITUTE | 1 | 0.035 |
| NEWCASTLE FREEMAN HOSPITAL | 1 | 0.035 |
| NEWCASTLE UPON TYNE HOSPITALS NHS FOUNDATION TRUST | 1 | 0.035 |
| NHS FDN TRUST | 1 | 0.035 |
| NHWATHAD PHARMACEUT CO LTD | 1 | 0.035 |
| NIH NATIONAL HEART LUNG BLOOD INSTITUTE NHLBI | 1 | 0.035 |
| NIH NATIONAL INSTITUTE OF ALLERGY INFECTIOUS DISEASES NIAID | 1 | 0.035 |
| NIHR NOTTINGHAM BIOMED RES CTR | 1 | 0.035 |
| NIJMEGEN INST INFECT INFLAMMAT IMMUN RUNMC | 1 | 0.035 |
| NINGBO 2 HOSP | 1 | 0.035 |
| NINGBO UROL NEPHROL HOSP | 1 | 0.035 |
| NINGXIA ENGN TECHNOL RES CTR MODERNIZAT HUI MED | 1 | 0.035 |
| NINGXIA MEDICAL UNIVERSITY | 1 | 0.035 |
| NINGXIA PEOPLES HOSP | 1 | 0.035 |
| NIPPON CHEMIPHAR CO LTD | 1 | 0.035 |
| NIPPON DENTAL UNIVERSITY | 1 | 0.035 |
| NIPPON ZOKI PHARMACEUT CO LTD | 1 | 0.035 |
| NIS LABS | 1 | 0.035 |
| NOAKHALI SCI TECHNOL UNIV | 1 | 0.035 |
| NORDIC INST CHIROPRACT CLIN BIOMECH | 1 | 0.035 |
| NORDLAND HOSP | 1 | 0.035 |
| NORMAN BETHUNE COLL MED | 1 | 0.035 |
| NORTHEASTERN UNIVERSITY | 1 | 0.035 |
| NORTHERN GENERAL HOSPITAL | 1 | 0.035 |
| NORTHERN ONTARIO SCH MED | 1 | 0.035 |
| NORTHSTAR CONSULTING LLC | 1 | 0.035 |
| NORTHWEST MINZU UNIVERSITY | 1 | 0.035 |
| NORTHWEST UNIVERSITY XI AN | 1 | 0.035 |
| NORWEGIAN INSTITUTE OF PUBLIC HEALTH NIPH | 1 | 0.035 |
| NORWEGIAN UNIVERSITY OF LIFE SCIENCES | 1 | 0.035 |
| NORWICH UNIV HOSP | 1 | 0.035 |
| NOVA SOUTHEASTERN UNIVERSITY | 1 | 0.035 |
| NOVARTIS ANIM HLTH KK | 1 | 0.035 |
| NOVO NORDISK | 1 | 0.035 |
| NUFFIELD ORTHOPAEDIC CENTRE | 1 | 0.035 |
| NUTRIPLAN CO LTD | 1 | 0.035 |
| NW UNIV MINOR | 1 | 0.035 |
| OAKLAND UNIVERSITY | 1 | 0.035 |
| OASI RES INST IRCCS | 1 | 0.035 |
| OBAFEMI AWOLOWO UNIVERSITY | 1 | 0.035 |
| OCCUPAT HLTH CTR | 1 | 0.035 |
| OCTOBER 6 UNIVERSITY O6U | 1 | 0.035 |
| OCULAR THERAPEUTIX INC | 1 | 0.035 |
| ODESSA STATE MEDICAL UNIVERSITY | 1 | 0.035 |
| OFF DIRECTOR WENCHENG CTY PEOPLES HOSP | 1 | 0.035 |
| OHIO UNIVERSITY | 1 | 0.035 |
| OIL CROPS RESEARCH INSTITUTE | 1 | 0.035 |
| OKINAKA MEM INST MED RES | 1 | 0.035 |
| OKLAHOMA STATE UNIVERSITY STILLWATER | 1 | 0.035 |
| OKLAHOMA STATE UNIVERSITY SYSTEM | 1 | 0.035 |
| OLIVENTURES | 1 | 0.035 |
| OLIVEPHARMASOLUTIONS LTD | 1 | 0.035 |
| OMMELANDER ZIEKENHUIS GRONINGEN | 1 | 0.035 |
| ONEOME LLC | 1 | 0.035 |
| ONO PHARMACEUT CO | 1 | 0.035 |
| ONO PHARMACEUT CO LTD | 1 | 0.035 |
| ONTARIO ADDICT TREATMENT CTR | 1 | 0.035 |
| ONTARIO TECH UNIVERSITY | 1 | 0.035 |
| OPEN UNIVERSITY UK | 1 | 0.035 |
| OPHTHALM CONSULTANTS LONG ISL | 1 | 0.035 |
| OPHTHALMOL CONSULTANTS | 1 | 0.035 |
| OPTUM | 1 | 0.035 |
| ORAL HLTH CTR EXPERTISE WESTERN NORWAY | 1 | 0.035 |
| ORDU STATE HOSPITAL | 1 | 0.035 |
| ORDU UNIV ORDU | 1 | 0.035 |
| ORGANOGENESIS INC | 1 | 0.035 |
| ORION CORP | 1 | 0.035 |
| ORION PHARMACEUT | 1 | 0.035 |
| ORTHOPAED RHEUMATOL N SHORE | 1 | 0.035 |
| ORTHOPAED TRAUMA SPECIALISTS | 1 | 0.035 |
| ORTHOPED UNIV HOSP FRIEDRICHSHEIM GGMBH | 1 | 0.035 |
| OSAKA INSTITUTE OF TECHNOLOGY | 1 | 0.035 |
| OSAKA OHTANI UNIV | 1 | 0.035 |
| OSMANGAZI M OZKALEKENT YAPI KOOP | 1 | 0.035 |
| OSPED A MANZONI | 1 | 0.035 |
| OSPED MAGENTA | 1 | 0.035 |
| OSPED RIUNITI HOSP | 1 | 0.035 |
| OSPED S GIUSEPPE | 1 | 0.035 |
| OSPEDALE CIRCOLO FONDAZIONE MACCHI | 1 | 0.035 |
| OSPEDALE SANT ANTONIO PADOVA | 1 | 0.035 |
| OSTFOLD CTY HOSP | 1 | 0.035 |
| OSTFOLD HOSP | 1 | 0.035 |
| OVERLOOK PAIN CTR | 1 | 0.035 |
| OVIDIUS UNIVERSITY | 1 | 0.035 |
| OXFORD UNIVERSITY HOSPITALS NHS FOUNDATION TRUST | 1 | 0.035 |
| PAD DR DY PATIL INST PHARMACEUT EDUC RES | 1 | 0.035 |
| PAIN TREATMENT CTR | 1 | 0.035 |
| PALO ALTO VET INST RES | 1 | 0.035 |
| PANACEA BIOTEC LTD | 1 | 0.035 |
| PAOLO PROCACCI FDN | 1 | 0.035 |
| PARACELSUS PRIVATE MED | 1 | 0.035 |
| PARC SANITARI SANT JOAN DE DEU | 1 | 0.035 |
| PARKER COLL RES INST | 1 | 0.035 |
| PARKER UNIV | 1 | 0.035 |
| PARV ESTADUAL LONDRINA | 1 | 0.035 |
| PASTEUR INSTITUTE OF IRAN | 1 | 0.035 |
| PATIENT RES PARTNER | 1 | 0.035 |
| PAUL EHRLICH INSTITUTE | 1 | 0.035 |
| PAUL SCHERRER INST | 1 | 0.035 |
| PAZMANY PETER CATHOLIC UNIV | 1 | 0.035 |
| PEDRO ERNESTO UNIV HOSP | 1 | 0.035 |
| PENNINE MUSCULOSKELETAL PARTNERSHIP LTD | 1 | 0.035 |
| PEOPLES FRIENDSHIP UNIVERSITY OF RUSSIA | 1 | 0.035 |
| PEOPLES HOSP DEYANG CITY | 1 | 0.035 |
| PEOPLES HOSP RIZHAO | 1 | 0.035 |
| PERSEUS PROTEOM INC | 1 | 0.035 |
| PETER MUNK CARDIAC CENTRE | 1 | 0.035 |
| PETRU PONI INSTITUTE OF MACROMOLECULAR CHEMISTRY | 1 | 0.035 |
| PETZ ALADAR CTY TEACHING HOSP | 1 | 0.035 |
| PFIZER AFRICA MIDDLE EAST REG | 1 | 0.035 |
| PFIZER ASIA PACIFIC REG | 1 | 0.035 |
| PFIZER INC | 1 | 0.035 |
| PFRN | 1 | 0.035 |
| PHARMALEADS SA | 1 | 0.035 |
| PHARMANET I3 | 1 | 0.035 |
| PHARMIN USA LLC | 1 | 0.035 |
| PHARMINVIVO LTD | 1 | 0.035 |
| PHILADELPHIA VETERANS AFFAIRS MEDICAL CENTER | 1 | 0.035 |
| PHOSPHAGEN LTD AUSTRALIA | 1 | 0.035 |
| PIANOWSKI PIANOWSKI LTDA | 1 | 0.035 |
| PIERRE FABRE RES CTR | 1 | 0.035 |
| PIRAMAL HEALTHCARE LTD | 1 | 0.035 |
| PITT COUNT MEM HOSP | 1 | 0.035 |
| PLA 117 HOSP | 1 | 0.035 |
| PLA GEN HOSP 100853 | 1 | 0.035 |
| POLICLIN MONZA HOSP | 1 | 0.035 |
| POLYCLIN HOSP BROS ST JOHN GOD | 1 | 0.035 |
| POLYCLIN HOSPITALLER BROTHERS ST JOHN GOD | 1 | 0.035 |
| PONTIFICIA UNIVERSIDADE CATOLICA DE MINAS GERAIS | 1 | 0.035 |
| PORTLAND VA MEDICAL CENTER | 1 | 0.035 |
| POWERED RES | 1 | 0.035 |
| PRATO HOSPITAL | 1 | 0.035 |
| PRESIDIO OSPED MARTINI | 1 | 0.035 |
| PRICE VIS GRP | 1 | 0.035 |
| PRIME HLTH CLIN | 1 | 0.035 |
| PRINCE FELIPE RESEARCH CENTER | 1 | 0.035 |
| PRINCE OF ASTURIAS UNIVERSITY HOSPITAL | 1 | 0.035 |
| PRINCE OF WALES HOSPITAL | 1 | 0.035 |
| PRINCE WALES HOSP | 1 | 0.035 |
| PRINCE WALES MEDICAL RESEARCH INSTITUTE | 1 | 0.035 |
| PRINCESS NORA BINT ABDUL RAHMAN UNIVERSITY | 1 | 0.035 |
| PROFILOMIC | 1 | 0.035 |
| PROGRAM IN CELLULAR MOLECULAR MEDICINE PCMM | 1 | 0.035 |
| PSG COLL PHARM | 1 | 0.035 |
| PSYCHOGENICS INC | 1 | 0.035 |
| PU AI HOSP WUHAN CITY | 1 | 0.035 |
| PUNJAB UNIV | 1 | 0.035 |
| PUNJABI UNIVERSITY | 1 | 0.035 |
| PUSAN NATIONAL UNIVERSITY HOSPITAL | 1 | 0.035 |
| QATAR UNIVERSITY | 1 | 0.035 |
| QILU UNIVERSITY OF TECHNOLOGY | 1 | 0.035 |
| QUANTUM VIS CTR | 1 | 0.035 |
| QUANZHOU MED COLL | 1 | 0.035 |
| QUEEN ELIZABETH UNIVERSITY HOSPITAL QEUH | 1 | 0.035 |
| R D INDENA SPA | 1 | 0.035 |
| R I | 1 | 0.035 |
| RABIN MEDICAL CENTER | 1 | 0.035 |
| RADBOUD UNIVERSITY NIJMEGEN | 1 | 0.035 |
| RADCLIFFE INFIRMARY | 1 | 0.035 |
| RADY CHILDRENS HOSPITAL SAN DIEGO | 1 | 0.035 |
| RAPID LABS INC | 1 | 0.035 |
| RAYMOND FONG EYE CARE | 1 | 0.035 |
| RC PATEL INST PHARMACEUT EDUC RES | 1 | 0.035 |
| RECEPTOPHARM INC | 1 | 0.035 |
| RECKITT BENCKISER | 1 | 0.035 |
| RED INVEST ACTIVIDADES PREVENT PROMOC SALUD RED | 1 | 0.035 |
| REDCLIFFE HOSP | 1 | 0.035 |
| REDWOOD REG ONCOL GRP | 1 | 0.035 |
| REG EDUC RES HOSP | 1 | 0.035 |
| REG HOSP SILKEBORG | 1 | 0.035 |
| REGIS UNIVERSITY | 1 | 0.035 |
| RENEURON LTD | 1 | 0.035 |
| RES INNOVAT CO | 1 | 0.035 |
| RES INST OSAKA MED CTR MATERNAL CHILD HLTH | 1 | 0.035 |
| RESEARCHED NUTR | 1 | 0.035 |
| RHEINISCHE FRIEDRICH WILHELMS UNIV | 1 | 0.035 |
| RHEUMATISM FOUNDATION HOSPITAL | 1 | 0.035 |
| RHEUMATOL GEMEINSCHAFTSPRAXIS | 1 | 0.035 |
| RHEUMATOL PRAXIS | 1 | 0.035 |
| RHODE ISLAND HOSPITAL | 1 | 0.035 |
| RHONE POULENC RORER FINLAND LTD | 1 | 0.035 |
| RIC BIOSCI LLC | 1 | 0.035 |
| RICHARD L ROUDEBUSH VA MEDICAL CENTER | 1 | 0.035 |
| RIKEN | 1 | 0.035 |
| RITA ALLEN FDN | 1 | 0.035 |
| RIVER OAKS PLAST SURG CTR | 1 | 0.035 |
| RIYADH MILITARY HOSPITAL | 1 | 0.035 |
| ROCKVILLE CTR | 1 | 0.035 |
| ROKAF | 1 | 0.035 |
| ROLLINS SCHOOL PUBLIC HEALTH | 1 | 0.035 |
| ROMANIAN ACADEMY OF SCIENCES | 1 | 0.035 |
| ROSALIND FRANKLIN UNIVERSITY MEDICAL SCIENCE | 1 | 0.035 |
| ROSIE MATERN HOSP | 1 | 0.035 |
| ROSS UNIV | 1 | 0.035 |
| ROTTERDAM UNIV APPL SCI | 1 | 0.035 |
| ROTUNDA HOSP | 1 | 0.035 |
| ROYAL ALBERT EDWARD INFIRMARY | 1 | 0.035 |
| ROYAL CHILDREN S HOSPITAL MELBOURNE | 1 | 0.035 |
| ROYAL COLLEGE OF SURGEONS IRELAND | 1 | 0.035 |
| ROYAL FREE LONDON NHS FOUNDATION TRUST | 1 | 0.035 |
| ROYAL LONDON HOSPITAL | 1 | 0.035 |
| ROYAL NATIONAL HOSPITAL FOR RHEUMATIC DISEASES RNHRD | 1 | 0.035 |
| ROYAL NATIONAL ORTHOPAEDIC HOSPITAL NHS TRUST | 1 | 0.035 |
| ROYAL NEWCASTLE CTR | 1 | 0.035 |
| ROYAL PERTH HOSPITAL | 1 | 0.035 |
| ROYAL SURREY COUNTY HOSPITAL | 1 | 0.035 |
| ROYAL UNITED HOSP | 1 | 0.035 |
| RUSH UNIVERSITY | 1 | 0.035 |
| RUSSELLS HALL HOSPITAL | 1 | 0.035 |
| RUTH RAPPAPORT FAC MED | 1 | 0.035 |
| RWTH AACHEN UNIVERSITY HOSPITAL | 1 | 0.035 |
| S TYRONE HOSP | 1 | 0.035 |
| SAARLAND UNIVERSITY | 1 | 0.035 |
| SAGA UNIVERSITY | 1 | 0.035 |
| SAINT MARIANNA UNIVERSITY | 1 | 0.035 |
| SAITAMA MEDICAL UNIVERSITY | 1 | 0.035 |
| SAKARYA UNIVERSITY | 1 | 0.035 |
| SALEM VETERANS AFFAIRS MEDICAL CENTER | 1 | 0.035 |
| SALFORD ROYAL HOSPITAL | 1 | 0.035 |
| SALFORD ROYAL NHS FOUNDATION TRUST | 1 | 0.035 |
| SALUGEN INC | 1 | 0.035 |
| SALZBURG GEN HOSP | 1 | 0.035 |
| SAN ANTONIO MILITARY MEDICAL CENTER | 1 | 0.035 |
| SAN CARLO BORROMEO HOSPITAL | 1 | 0.035 |
| SAN DIEGO STATE UNIVERSITY | 1 | 0.035 |
| SAN DIEGO VET AFFAIRS HEALTHCARE SYST | 1 | 0.035 |
| SAN FILIPPO NERI HOSPITAL | 1 | 0.035 |
| SAN FRANCISCO VA MEDICAL CENTER | 1 | 0.035 |
| SAN GERARDO HOSPITAL | 1 | 0.035 |
| SAN GIACOMO HOSP | 1 | 0.035 |
| SANDWELL WEST BIRMINGHAM HOSP NHS TRUST | 1 | 0.035 |
| SANFORD BURNHAM PREBYS MEDICAL DISCOVERY INSTITUTE | 1 | 0.035 |
| SANJAY GANDHI POSTGRADUATE INSTITUTE OF MEDICAL SCIENCES | 1 | 0.035 |
| SANTA CASA BELO HORIZONTE | 1 | 0.035 |
| SANTA CHIARA HOSPITAL | 1 | 0.035 |
| SANTA MARIA ANNUNZIATA HOSP | 1 | 0.035 |
| SANTISSIMA TRINITA HOSP | 1 | 0.035 |
| SAO JOAO HOSPITAL | 1 | 0.035 |
| SAO LEOPOLDO MAND INST RESEARCHER CTR | 1 | 0.035 |
| SB DRUG DISCOVERY LTD | 1 | 0.035 |
| SCHERING PLOUGH RESEARCH INSTITUTE | 1 | 0.035 |
| SCHLOSSPK KLIN | 1 | 0.035 |
| SCHLUMBERGER | 1 | 0.035 |
| SCHNEIDER CHILDRENS MED CTR | 1 | 0.035 |
| SCHRODINGER INC | 1 | 0.035 |
| SCHULTHESS CLINIC | 1 | 0.035 |
| SCHWARZ BIOSCI GMBH | 1 | 0.035 |
| SCIOS | 1 | 0.035 |
| SCOTT CHRISTIE ASSOC PC | 1 | 0.035 |
| SCRIPPS MEM HOSP XI MED | 1 | 0.035 |
| SE LA VET HLTH CARE SYST | 1 | 0.035 |
| SE TEXAS REHABIL HOSP | 1 | 0.035 |
| SECOND HOSP HEBEI MED UNIV | 1 | 0.035 |
| SECOND PEOPLES HOSP NANTONG | 1 | 0.035 |
| SEETA EYE CTR | 1 | 0.035 |
| SEINAJOKI CENTRAL HOSPITAL | 1 | 0.035 |
| SEJONG UNIVERSITY | 1 | 0.035 |
| SEMEY STATE MEDICAL UNIVERSITY | 1 | 0.035 |
| SEOIL UNIV | 1 | 0.035 |
| SEOUL VETERANS HOSPITAL | 1 | 0.035 |
| SERGIO FRANCO LAB | 1 | 0.035 |
| SERONO RES INST | 1 | 0.035 |
| SERVIER | 1 | 0.035 |
| SGPGIMS | 1 | 0.035 |
| SHAANXI UNIVERSITY OF CHINESE MEDICINE | 1 | 0.035 |
| SHAHED UNIVERSITY | 1 | 0.035 |
| SHAHEED BENAZIR BHUTTO UNIV | 1 | 0.035 |
| SHAHID BEHESHTI UNIVERSITY | 1 | 0.035 |
| SHAHID SADOUGHI UNIV MED SCI | 1 | 0.035 |
| SHAHREKORD UNIVERSITY | 1 | 0.035 |
| SHANDONG TAISHAN CHRON DIS HOSP | 1 | 0.035 |
| SHANGHAI 411 HOSP | 1 | 0.035 |
| SHANGHAI GONGLI HOSP | 1 | 0.035 |
| SHANGHAI JIANGWAN HOSP | 1 | 0.035 |
| SHANGHAI MED COLL | 1 | 0.035 |
| SHANGHAI PUDONG HOSP | 1 | 0.035 |
| SHANGHAI RES CTR ACUPUNCTURE MERIDIAN | 1 | 0.035 |
| SHANGHAI TCM INTEGRATED HOSP | 1 | 0.035 |
| SHANGHAI YILE BIOTECHNOL CO LTD | 1 | 0.035 |
| SHANXI UNIVERSITY OF CHINESE MEDICINE | 1 | 0.035 |
| SHAOXING PEOPLES HOSP | 1 | 0.035 |
| SHAOXING SECOND HOSP | 1 | 0.035 |
| SHAOXING TRADIT CHINESE MED HOSP | 1 | 0.035 |
| SHAR TEACHING HOSP | 1 | 0.035 |
| SHASTA EYE MED GRP | 1 | 0.035 |
| SHERWOOD FOREST HOSP NHS FDN TRUST | 1 | 0.035 |
| SHIFA COLLEGE OF MEDICINE | 1 | 0.035 |
| SHIFA TAMEER E MILLAT UNIV | 1 | 0.035 |
| SHIHEZI UNIVERSITY | 1 | 0.035 |
| SHIN KONG WU HO SU MEMORIAL HOSPITAL | 1 | 0.035 |
| SHRINERS HOSP CHILDREN | 1 | 0.035 |
| SHRINERS HOSPITALS CHILDREN PHILADELPHIA | 1 | 0.035 |
| SHUANG HO HOSPITAL | 1 | 0.035 |
| SHUJITSU UNIV | 1 | 0.035 |
| SIBIA NEUROSCI INC | 1 | 0.035 |
| SICHUAN PROVINCIAL PEOPLE S HOSPITAL | 1 | 0.035 |
| SIGMA TAU PHARMACEUT CO | 1 | 0.035 |
| SILKEBORG CENTRAL HOSPITAL | 1 | 0.035 |
| SILVERSTEIN EYE CTR | 1 | 0.035 |
| SIME DARBY MED CTR | 1 | 0.035 |
| SIMPAR GRP STUDY MULTIDISCIPLINARY PAIN RES | 1 | 0.035 |
| SINGULEX INC | 1 | 0.035 |
| SIRION THERAPEUT | 1 | 0.035 |
| SITEMAN CANCER CENTER | 1 | 0.035 |
| SIXTH HOSP SHIJIAZHUANG | 1 | 0.035 |
| SJOGRENS SYNDROME FDN | 1 | 0.035 |
| SLAGELSE HOSP | 1 | 0.035 |
| SMBD JEWISH GEN HOSP | 1 | 0.035 |
| SO ORTHOPAED SPORTS MED | 1 | 0.035 |
| SOC WOMENS HLTH RES | 1 | 0.035 |
| SOCIAL INSURANCE INST | 1 | 0.035 |
| SOCRATEC R D GMBH | 1 | 0.035 |
| SONDALO HOSP | 1 | 0.035 |
| SOOCHOW UNIV | 1 | 0.035 |
| SOONCHUNHYANG UNIVERSITY | 1 | 0.035 |
| SORLANDET HOSP | 1 | 0.035 |
| SORLANDET HOSP ARENDAL | 1 | 0.035 |
| SOUTH CHINA AGRICULTURAL UNIVERSITY | 1 | 0.035 |
| SOUTH CHINA UNIVERSITY OF TECHNOLOGY | 1 | 0.035 |
| SOUTH DAKOTA STATE UNIVERSITY | 1 | 0.035 |
| SOUTHEAST LOUISIANA VET HLTH CARE SYST | 1 | 0.035 |
| SOUTHERN TAIWAN UNIVERSITY OF SCIENCE TECHNOLOGY | 1 | 0.035 |
| SOUTHERN THEATER COMMAND GENERAL HOSPITAL | 1 | 0.035 |
| SOUTHLAKE REGIONAL HEALTH CENTRE | 1 | 0.035 |
| SOUTHMEAD HOSPITAL | 1 | 0.035 |
| SOUTHWEST UNIVERSITY CHINA | 1 | 0.035 |
| SPANISH AGCY MED CLIN DEVICES AEMPS | 1 | 0.035 |
| SPANISH CTR PHARMACOEPIDEMIOL RES CEIFE | 1 | 0.035 |
| SPINE CTR GOTEBORG | 1 | 0.035 |
| SPINE NERVE CTR VIRGINIAS | 1 | 0.035 |
| SPITAL WETZIKON | 1 | 0.035 |
| SPORTS ORTHOPED REHABIL MED ASSOC | 1 | 0.035 |
| SPRINGER NATURE | 1 | 0.035 |
| ST ANNE S UNIVERSITY HOSPITAL BRNO FNUSA ICRC | 1 | 0.035 |
| ST CATHERINE SPECIALTY HOSP | 1 | 0.035 |
| ST CHARLES HOSP | 1 | 0.035 |
| ST ELIZABETH S MEDICAL CENTER | 1 | 0.035 |
| ST GEORGES UNIV HOSP NHS FDN TRUST | 1 | 0.035 |
| ST HELENS HOSP | 1 | 0.035 |
| ST JOSEFS SENDENHORST | 1 | 0.035 |
| ST PETERSBURG COLL | 1 | 0.035 |
| ST VINCENTS HOSPITAL SYDNEY | 1 | 0.035 |
| STAFFORDSHIRE RHEUMATOL CTR | 1 | 0.035 |
| STAT OFF REPUBL SERBIA | 1 | 0.035 |
| STATE ART CHIROPRACT CTR | 1 | 0.035 |
| STATE HOSP STOCKERAU | 1 | 0.035 |
| STATE KEY LAB MOL CELL BIOL | 1 | 0.035 |
| STATE UNIVERSITY OF NEW YORK SUNY ALBANY | 1 | 0.035 |
| STATE UNIVERSITY OF NEW YORK SUNY DOWNSTATE MEDICAL CENTER | 1 | 0.035 |
| STESS SINHGAD INST PHARM | 1 | 0.035 |
| STEVEN ALEXANDRA COHEN CHILDREN S MEDICAL CENTER OF NEW YORK | 1 | 0.035 |
| STOCKHOLM EYE CLIN | 1 | 0.035 |
| STOCKHOLM UNIVERSITY | 1 | 0.035 |
| STRACATHRO HOSP | 1 | 0.035 |
| STUDY MULTIDISCIPLINARY PAIN RES SIMPAR | 1 | 0.035 |
| SUEZ CANAL UNIVERSITY | 1 | 0.035 |
| SULEYMAN DEMIREL UNIVERSITY | 1 | 0.035 |
| SULTAN QABOOS UNIVERSITY | 1 | 0.035 |
| SUNY MARITIME COLLEGE | 1 | 0.035 |
| SUVEN LIFE SCI LTD | 1 | 0.035 |
| SUZHOU SCI TECHNOL TOWN HOSP | 1 | 0.035 |
| SVKMS INST PHARM | 1 | 0.035 |
| SYNAPATAMINE INC | 1 | 0.035 |
| SYNAPT PHARMACEUT CORP | 1 | 0.035 |
| SYNERGIA PHARMA INC | 1 | 0.035 |
| SYST LINKED RES UNIT | 1 | 0.035 |
| SZENT IMRE HOSP | 1 | 0.035 |
| TAIBAH UNIVERSITY | 1 | 0.035 |
| TAICHUNG ARMED FORCES GEN HOSP | 1 | 0.035 |
| TAIPEI CITY HOSPITAL | 1 | 0.035 |
| TAIPEI HOSP | 1 | 0.035 |
| TAIPEI MEDICAL UNIVERSITY HOSPITAL | 1 | 0.035 |
| TAISHO TOYAMA PHARMACEUT CO LTD | 1 | 0.035 |
| TAKEDA PHARMACEUT INT | 1 | 0.035 |
| TAKEDA PHARMACEUTICAL COMPANY LTD | 1 | 0.035 |
| TAMANA CENT HOSP | 1 | 0.035 |
| TARGETED MED PHARMA INC | 1 | 0.035 |
| TARLETON STATE UNIV | 1 | 0.035 |
| TATAA BIOCTR | 1 | 0.035 |
| TAYLOR MONROE GRAVEL HEAD FARM | 1 | 0.035 |
| TECHNICAL UNIVERSITY OF DARMSTADT | 1 | 0.035 |
| TECHNICAL UNIVERSITY OF MUNICH | 1 | 0.035 |
| TECHNISCHE UNIVERSITAT DRESDEN | 1 | 0.035 |
| TEIJIN PHARMA LTD | 1 | 0.035 |
| TEXAS HEART INSTITUTE | 1 | 0.035 |
| TEXAS STATE UNIVERSITY SYSTEM | 1 | 0.035 |
| THERACHEM RES MEDILAB INDIA PVT LTD | 1 | 0.035 |
| TIANJIN CENT HOSP | 1 | 0.035 |
| TIANJIN HOSP | 1 | 0.035 |
| TIANJIN MEDICAL UNIVERSITY | 1 | 0.035 |
| TIANJIN NANKAI HOSP | 1 | 0.035 |
| TIANJIN UNIVERSITY OF TRADITIONAL CHINESE MEDICINE | 1 | 0.035 |
| TOHO UNIV | 1 | 0.035 |
| TOSHIBA CORPORATION | 1 | 0.035 |
| TOWNSVILLE GEN HOSP | 1 | 0.035 |
| TRANSWORLD INST TECHNOL | 1 | 0.035 |
| TRIEMLI HOSPITAL | 1 | 0.035 |
| TRINITY COLLEGE | 1 | 0.035 |
| TRIPURA UNIVERSITY | 1 | 0.035 |
| TRP RES PLATFORM LEUVEN TRPLE | 1 | 0.035 |
| TSHWANE UNIVERSITY OF TECHNOLOGY | 1 | 0.035 |
| TULANE BRAIN INST | 1 | 0.035 |
| TURKISH ARMED FORCES REHABILITATION MAINTENANCE CENTER | 1 | 0.035 |
| TURKISH MED MED DEVICES AGCY | 1 | 0.035 |
| TURKU BRAIN MIND CTR | 1 | 0.035 |
| UCLA HLTH SYST | 1 | 0.035 |
| UCONN HLTH | 1 | 0.035 |
| UFS | 1 | 0.035 |
| UK HEALTHCARE SPINE TOTAL JOINT SERV | 1 | 0.035 |
| UKZ LINZ | 1 | 0.035 |
| ULM UNIVERSITY | 1 | 0.035 |
| ULSS 6 EUGANEA | 1 | 0.035 |
| UMM AL QURA UNIVERSITY | 1 | 0.035 |
| UNICANCER | 1 | 0.035 |
| UNIFORMED SERVICE UNIV | 1 | 0.035 |
| UNION HOSP | 1 | 0.035 |
| UNION MEMORIAL HOSPITAL | 1 | 0.035 |
| UNITED MED RES INST | 1 | 0.035 |
| UNITED STATES AIR FORCE | 1 | 0.035 |
| UNIV ALCALA IRYCIS | 1 | 0.035 |
| UNIV APPL SCI | 1 | 0.035 |
| UNIV ARARAQUARA UNIARA | 1 | 0.035 |
| UNIV AUTONOMA CHIHUAHUA | 1 | 0.035 |
| UNIV BELVITGE | 1 | 0.035 |
| UNIV BRASIL UNIBRASIL | 1 | 0.035 |
| UNIV CATOLICA ANDRES BELLO | 1 | 0.035 |
| UNIV EASTERN FINLAND KUOPIO | 1 | 0.035 |
| UNIV ESTADUAL JULIO DE MESQUITA FILHO | 1 | 0.035 |
| UNIV ESTADUAL LONDRINA | 1 | 0.035 |
| UNIV FARM | 1 | 0.035 |
| UNIV FED VALE JEQUITINHONHA MUCURI | 1 | 0.035 |
| UNIV FRENCH W INDIES | 1 | 0.035 |
| UNIV HOSP | 1 | 0.035 |
| UNIV HOSP GRAZ | 1 | 0.035 |
| UNIV HOSP SVETI DUH | 1 | 0.035 |
| UNIV LIGE | 1 | 0.035 |
| UNIV MARYLAND | 1 | 0.035 |
| UNIV METROPOLITANA | 1 | 0.035 |
| UNIV NOTTINGHAM MIDLANDS | 1 | 0.035 |
| UNIV PARIS 05 | 1 | 0.035 |
| UNIV PARIS EST | 1 | 0.035 |
| UNIV SAN DIEGO HAHN SCH NURSING HLTH SCI | 1 | 0.035 |
| UNIV SCH PHYS EDUC POZNAN | 1 | 0.035 |
| UNIV SCI TECHNOL | 1 | 0.035 |
| UNIV STRASBOURG USIAS | 1 | 0.035 |
| UNIV SWABI | 1 | 0.035 |
| UNIV SWAT | 1 | 0.035 |
| UNIV TEKNOL MARA UITM | 1 | 0.035 |
| UNIV TEXAS SCI CTR HOUSTON | 1 | 0.035 |
| UNIV VAL PARAIBA | 1 | 0.035 |
| UNIVERSIDAD AMERICAS PUEBLA UDLAP | 1 | 0.035 |
| UNIVERSIDAD ANDRES BELLO | 1 | 0.035 |
| UNIVERSIDAD AUTONOMA DE SAN LUIS POTOSI | 1 | 0.035 |
| UNIVERSIDAD AUTONOMA DE SINALOA | 1 | 0.035 |
| UNIVERSIDAD AUTONOMA DEL ESTADO DE HIDALGO | 1 | 0.035 |
| UNIVERSIDAD COSTA RICA | 1 | 0.035 |
| UNIVERSIDAD DE CONCEPCION | 1 | 0.035 |
| UNIVERSIDAD DE CORDOBA | 1 | 0.035 |
| UNIVERSIDAD DE EL SALVADOR | 1 | 0.035 |
| UNIVERSIDAD DE GUADALAJARA | 1 | 0.035 |
| UNIVERSIDAD DE LA LAGUNA | 1 | 0.035 |
| UNIVERSIDAD DE PANAMA | 1 | 0.035 |
| UNIVERSIDAD INDUSTRIAL DE SANTANDER | 1 | 0.035 |
| UNIVERSIDAD NACIONAL DE EDUCACION A DISTANCIA UNED | 1 | 0.035 |
| UNIVERSIDADE CEUMA | 1 | 0.035 |
| UNIVERSIDADE DE COIMBRA | 1 | 0.035 |
| UNIVERSIDADE DE PERNAMBUCO UPE | 1 | 0.035 |
| UNIVERSIDADE DE UBERABA UNIUBE | 1 | 0.035 |
| UNIVERSIDADE DO ESTADO DE SANTA CATARINA | 1 | 0.035 |
| UNIVERSIDADE DO SAGRADO CORACAO | 1 | 0.035 |
| UNIVERSIDADE DO VALE DO RIO DOS SINOS UNISINOS | 1 | 0.035 |
| UNIVERSIDADE ESTADUAL DE FEIRA DE SANTANA | 1 | 0.035 |
| UNIVERSIDADE ESTADUAL DE MARINGA | 1 | 0.035 |
| UNIVERSIDADE ESTADUAL DE MATO GROSSO DO SUL | 1 | 0.035 |
| UNIVERSIDADE ESTADUAL DO PIAUI UESPI | 1 | 0.035 |
| UNIVERSIDADE FEDERAL DA GRANDE DOURADOS | 1 | 0.035 |
| UNIVERSIDADE FEDERAL DE JUIZ DE FORA | 1 | 0.035 |
| UNIVERSIDADE FEDERAL DE MATO GROSSO DO SUL | 1 | 0.035 |
| UNIVERSIDADE FEDERAL DE PELOTAS | 1 | 0.035 |
| UNIVERSIDADE FEDERAL DO ESPIRITO SANTO | 1 | 0.035 |
| UNIVERSIDADE FEDERAL DO ESTADO DO RIO DE JANEIRO | 1 | 0.035 |
| UNIVERSIDADE FEDERAL DO MARANHAO | 1 | 0.035 |
| UNIVERSIDADE FEDERAL DO PAMPA | 1 | 0.035 |
| UNIVERSIDADE FEDERAL DO PARA | 1 | 0.035 |
| UNIVERSIDADE FEDERAL DOS VALES DO JEQUITINHONHA E MUCURI UFVJM | 1 | 0.035 |
| UNIVERSIDADE FORTALEZA | 1 | 0.035 |
| UNIVERSIDADE LUTERANA DO BRASIL | 1 | 0.035 |
| UNIVERSIDADE NOVA DE LISBOA | 1 | 0.035 |
| UNIVERSIDADE REGIONAL DE BLUMENAU FURB | 1 | 0.035 |
| UNIVERSITA DEGLI STUDI DI BARI ALDO MORO | 1 | 0.035 |
| UNIVERSITA DELLA SVIZZERA ITALIANA | 1 | 0.035 |
| UNIVERSITA KORE DI ENNA | 1 | 0.035 |
| UNIVERSITAS JEMBER | 1 | 0.035 |
| UNIVERSITAS PADJADJARAN | 1 | 0.035 |
| UNIVERSITAT D ALACANT | 1 | 0.035 |
| UNIVERSITAT DE GIRONA | 1 | 0.035 |
| UNIVERSITE CLAUDE BERNARD LYON 1 | 1 | 0.035 |
| UNIVERSITE D ANGERS | 1 | 0.035 |
| UNIVERSITE DE LORRAINE | 1 | 0.035 |
| UNIVERSITE DE ORLEANS | 1 | 0.035 |
| UNIVERSITE DE TOURS | 1 | 0.035 |
| UNIVERSITE JEAN MONNET | 1 | 0.035 |
| UNIVERSITI KEBANGSAAN MALAYSIA | 1 | 0.035 |
| UNIVERSITI PUTRA MALAYSIA | 1 | 0.035 |
| UNIVERSITI SAINS MALAYSIA | 1 | 0.035 |
| UNIVERSITI TEKNOLOGI MARA | 1 | 0.035 |
| UNIVERSITY CAMPUS BIO MEDICO ROME ITALY | 1 | 0.035 |
| UNIVERSITY CHILDREN S HOSPITAL ZURICH | 1 | 0.035 |
| UNIVERSITY COLLEGE HOSPITAL IBADAN | 1 | 0.035 |
| UNIVERSITY HOSPITAL BRUSSELS | 1 | 0.035 |
| UNIVERSITY HOSPITAL OF NORTH NORWAY | 1 | 0.035 |
| UNIVERSITY HOSPITAL OF NORTH STAFFORDSHIRE NHS TRUST | 1 | 0.035 |
| UNIVERSITY HOSPITAL OF PARMA | 1 | 0.035 |
| UNIVERSITY HOSPITAL SOUTHAMPTON NHS FOUNDATION TRUST | 1 | 0.035 |
| UNIVERSITY HOSPITAL VIENNA | 1 | 0.035 |
| UNIVERSITY NACIONAL CUYO MENDOZA | 1 | 0.035 |
| UNIVERSITY OF ANTWERP | 1 | 0.035 |
| UNIVERSITY OF AQUILA | 1 | 0.035 |
| UNIVERSITY OF BRIGHTON | 1 | 0.035 |
| UNIVERSITY OF BUCHAREST | 1 | 0.035 |
| UNIVERSITY OF CALABRIA | 1 | 0.035 |
| UNIVERSITY OF CALIFORNIA BERKELEY | 1 | 0.035 |
| UNIVERSITY OF CENTRAL LANCASHIRE | 1 | 0.035 |
| UNIVERSITY OF CENTRAL VENEZUELA | 1 | 0.035 |
| UNIVERSITY OF CHICAGO MEDICAL CENTER | 1 | 0.035 |
| UNIVERSITY OF COLORADO DENVER | 1 | 0.035 |
| UNIVERSITY OF CYPRUS | 1 | 0.035 |
| UNIVERSITY OF DELAWARE | 1 | 0.035 |
| UNIVERSITY OF EASTERN FINLAND | 1 | 0.035 |
| UNIVERSITY OF EXETER | 1 | 0.035 |
| UNIVERSITY OF FREIBURG | 1 | 0.035 |
| UNIVERSITY OF GRAZ | 1 | 0.035 |
| UNIVERSITY OF HAIFA | 1 | 0.035 |
| UNIVERSITY OF HANNOVER | 1 | 0.035 |
| UNIVERSITY OF HERTFORDSHIRE | 1 | 0.035 |
| UNIVERSITY OF IBADAN | 1 | 0.035 |
| UNIVERSITY OF ILLINOIS URBANA CHAMPAIGN | 1 | 0.035 |
| UNIVERSITY OF ILORIN | 1 | 0.035 |
| UNIVERSITY OF INDONESIA | 1 | 0.035 |
| UNIVERSITY OF INSUBRIA | 1 | 0.035 |
| UNIVERSITY OF JJ STROSSMAYER OSIJEK | 1 | 0.035 |
| UNIVERSITY OF JORDAN | 1 | 0.035 |
| UNIVERSITY OF KARACHI | 1 | 0.035 |
| UNIVERSITY OF KHARTOUM | 1 | 0.035 |
| UNIVERSITY OF KIEL | 1 | 0.035 |
| UNIVERSITY OF KRAGUJEVAC | 1 | 0.035 |
| UNIVERSITY OF KWAZULU NATAL | 1 | 0.035 |
| UNIVERSITY OF LAGOS | 1 | 0.035 |
| UNIVERSITY OF LJUBLJANA | 1 | 0.035 |
| UNIVERSITY OF LUBECK | 1 | 0.035 |
| UNIVERSITY OF MAINE | 1 | 0.035 |
| UNIVERSITY OF MAINE ORONO | 1 | 0.035 |
| UNIVERSITY OF MANITOBA | 1 | 0.035 |
| UNIVERSITY OF MASSACHUSETTS AMHERST | 1 | 0.035 |
| UNIVERSITY OF MEMPHIS | 1 | 0.035 |
| UNIVERSITY OF MISSISSIPPI | 1 | 0.035 |
| UNIVERSITY OF MISSISSIPPI MEDICAL CENTER | 1 | 0.035 |
| UNIVERSITY OF MISSOURI COLUMBIA | 1 | 0.035 |
| UNIVERSITY OF MISSOURI SAINT LOUIS | 1 | 0.035 |
| UNIVERSITY OF MIYAZAKI | 1 | 0.035 |
| UNIVERSITY OF MONTANA | 1 | 0.035 |
| UNIVERSITY OF MONTANA SYSTEM | 1 | 0.035 |
| UNIVERSITY OF NEBRASKA MEDICAL CENTER | 1 | 0.035 |
| UNIVERSITY OF NEBRASKA SYSTEM | 1 | 0.035 |
| UNIVERSITY OF NEVADA LAS VEGAS | 1 | 0.035 |
| UNIVERSITY OF NEW ENGLAND | 1 | 0.035 |
| UNIVERSITY OF NEW ENGLAND MAINE | 1 | 0.035 |
| UNIVERSITY OF NICOSIA | 1 | 0.035 |
| UNIVERSITY OF NIZWA | 1 | 0.035 |
| UNIVERSITY OF NORTH TEXAS DENTON | 1 | 0.035 |
| UNIVERSITY OF NORTH TEXAS SYSTEM | 1 | 0.035 |
| UNIVERSITY OF NOTRE DAME AUSTRALIA | 1 | 0.035 |
| UNIVERSITY OF NOVA GORICA | 1 | 0.035 |
| UNIVERSITY OF NOVI SAD | 1 | 0.035 |
| UNIVERSITY OF OKLAHOMA NORMAN | 1 | 0.035 |
| UNIVERSITY OF ORADEA | 1 | 0.035 |
| UNIVERSITY OF PERADENIYA | 1 | 0.035 |
| UNIVERSITY OF PISA | 1 | 0.035 |
| UNIVERSITY OF RIJEKA | 1 | 0.035 |
| UNIVERSITY OF ROME TOR VERGATA | 1 | 0.035 |
| UNIVERSITY OF SALFORD | 1 | 0.035 |
| UNIVERSITY OF SASSARI | 1 | 0.035 |
| UNIVERSITY OF SHIZUOKA | 1 | 0.035 |
| UNIVERSITY OF SOUTH DAKOTA | 1 | 0.035 |
| UNIVERSITY OF SOUTHERN MAINE | 1 | 0.035 |
| UNIVERSITY OF SULIMANYAH | 1 | 0.035 |
| UNIVERSITY OF TABUK | 1 | 0.035 |
| UNIVERSITY OF TENNESSEE KNOXVILLE | 1 | 0.035 |
| UNIVERSITY OF TENNESSEE SYSTEM | 1 | 0.035 |
| UNIVERSITY OF TERAMO | 1 | 0.035 |
| UNIVERSITY OF TEXAS AT SAN ANTONIO UTSA | 1 | 0.035 |
| UNIVERSITY OF THE PACIFIC | 1 | 0.035 |
| UNIVERSITY OF THE PHILIPPINES MANILA | 1 | 0.035 |
| UNIVERSITY OF THE PHILIPPINES SYSTEM | 1 | 0.035 |
| UNIVERSITY OF THE SUNSHINE COAST | 1 | 0.035 |
| UNIVERSITY OF THE WEST INDIES | 1 | 0.035 |
| UNIVERSITY OF TRIESTE | 1 | 0.035 |
| UNIVERSITY OF UDINE | 1 | 0.035 |
| UNIVERSITY OF WARMIA MAZURY | 1 | 0.035 |
| UNIVERSITY OF WATERLOO | 1 | 0.035 |
| UNIVERSITY OF WITWATERSRAND | 1 | 0.035 |
| UNIVERSITY OF YAMANASHI | 1 | 0.035 |
| UNIVERSITY OF YAOUNDE I | 1 | 0.035 |
| UOSD MED INTERNA AMBULATORIO ANDROL ENDOCRINOL | 1 | 0.035 |
| UPPSALA UNIVERSITY HOSPITAL | 1 | 0.035 |
| UPRESS EA 3892 | 1 | 0.035 |
| US AIR FORCE RESEARCH LABORATORY | 1 | 0.035 |
| USIAS | 1 | 0.035 |
| USMANU DANFODIYO UNIV SOKOTO | 1 | 0.035 |
| UT INSTITUTE OF AGRICULTURE | 1 | 0.035 |
| UTTARANCHAL DENT MED RES INST | 1 | 0.035 |
| UZSOKI HOSP | 1 | 0.035 |
| VA CTR EXCELLENCE SUICIDE PREVENT | 1 | 0.035 |
| VA MED CTR | 1 | 0.035 |
| VA NORTHERN CALIF HLTH CARE SYST | 1 | 0.035 |
| VACA RESOURCES | 1 | 0.035 |
| VEJLE HOSPITAL | 1 | 0.035 |
| VERACT INTRACTABLE PAIN CLIN | 1 | 0.035 |
| VERTEX PHARMACEUTICALS | 1 | 0.035 |
| VESTRE VIKEN HOSP TRUST | 1 | 0.035 |
| VET ADM SAN DIEGO HEALTHCARE SYST | 1 | 0.035 |
| VET AFFAIRS HEALTHCARE SYST | 1 | 0.035 |
| VET AFFAIRS HLTH SYST | 1 | 0.035 |
| VET AFFAIRS PUGET SOUND HEALTH CARE SYSTEM | 1 | 0.035 |
| VET AGRI HLTH SERV LTD | 1 | 0.035 |
| VET ASSOC PITTSBURGH HEALTHCARE SYST | 1 | 0.035 |
| VET EXOT CTR EXOTICVET | 1 | 0.035 |
| VET PRACTICE | 1 | 0.035 |
| VET STN KARLOVAC | 1 | 0.035 |
| VETAGRO SUP | 1 | 0.035 |
| VETBIOBANK SAS | 1 | 0.035 |
| VICTOR BABES UNIVERSITY OF MEDICINE PHARMACY TIMISOARA | 1 | 0.035 |
| VIENNA BIOCENTER VBC | 1 | 0.035 |
| VIILA IGEA | 1 | 0.035 |
| VILLA VERDE | 1 | 0.035 |
| VIRGINIA MASON MEDICAL CENTER | 1 | 0.035 |
| VIRGINIA POLYTECHNIC INSTITUTE STATE UNIVERSITY | 1 | 0.035 |
| VISCERAL INFLAMMAT PAIN CTR | 1 | 0.035 |
| VOLTA RES | 1 | 0.035 |
| VOLVAT MED CTR | 1 | 0.035 |
| VORONOI INC | 1 | 0.035 |
| WAKAYAMA MED UNIV | 1 | 0.035 |
| WALTER ELIZA HALL INSTITUTE | 1 | 0.035 |
| WALTER REED ARMY INSTITUTE OF RESEARCH WRAIR | 1 | 0.035 |
| WARSAW UNIVERSITY OF LIFE SCIENCES | 1 | 0.035 |
| WEIHAI CENT HOSP | 1 | 0.035 |
| WEILL CORNELL MEDICAL COLLEGE QATAR | 1 | 0.035 |
| WELLCOME RESEARCH LABORATORIES | 1 | 0.035 |
| WELLCOME TRUST SANGER INSTITUTE | 1 | 0.035 |
| WENCHENG CTY PEOPLES HOSP | 1 | 0.035 |
| WENZHOU CENT HOSP | 1 | 0.035 |
| WEST HERTFORDSHIRE HOSP NHS TRUST | 1 | 0.035 |
| WEST HOE SURG | 1 | 0.035 |
| WESTCHESTER HEAD NECK PAIN CTR | 1 | 0.035 |
| WESTCHESTER MEDICAL CENTER | 1 | 0.035 |
| WESTERN HLTH | 1 | 0.035 |
| WESTERN PSYCHIATRIC INSTITUTE CLINIC OF UPMC | 1 | 0.035 |
| WESTERN SYDNEY UNIVERSITY | 1 | 0.035 |
| WFIRM | 1 | 0.035 |
| WILHELMINA GASTHUIS | 1 | 0.035 |
| WINTHROP UNIVERSITY HOSPITAL | 1 | 0.035 |
| WIP | 1 | 0.035 |
| WITTEN HERDECKE UNIV | 1 | 0.035 |
| WOCKHARDT HOSP | 1 | 0.035 |
| WOLFSON CARD | 1 | 0.035 |
| WOLSTAN GOLDBERG EYE ASSOCIATES | 1 | 0.035 |
| WOMENS COLLEGE HOSPITAL | 1 | 0.035 |
| WORLD HEALTH ORGANIZATION | 1 | 0.035 |
| WUHAN 1 HOSP | 1 | 0.035 |
| WUHAN FIRST HOSP | 1 | 0.035 |
| WUHAN HANYANG HOSP | 1 | 0.035 |
| WUHAN INSTITUTE OF PHYSICS MATHEMATICS CAS | 1 | 0.035 |
| WUHAN INTEGRATED TCM WESTERN MED HOSP | 1 | 0.035 |
| WUHAN UNIVERSITY OF SCIENCE TECHNOLOGY | 1 | 0.035 |
| WUXI APPTEC | 1 | 0.035 |
| XENON PHARMACEUT INC | 1 | 0.035 |
| XI AN MEDICAL UNIVERSITY | 1 | 0.035 |
| XIAMEN UNIVERSITY | 1 | 0.035 |
| XIAN HOSP TRADIT CHINESE MED | 1 | 0.035 |
| XIAN JIAOTONG UNIV SCH MED | 1 | 0.035 |
| XINGTAI MED COLL | 1 | 0.035 |
| XINJIANG MEDICAL UNIVERSITY | 1 | 0.035 |
| XUZHOU CENT HOSP | 1 | 0.035 |
| YAMAGUCHI UNIVERSITY | 1 | 0.035 |
| YANAN UNIVERSITY | 1 | 0.035 |
| YANBIAN UNIVERSITY | 1 | 0.035 |
| YANGGU 2 PEOPLES HOSP | 1 | 0.035 |
| YANGTZE UNIVERSITY | 1 | 0.035 |
| YANSHAN UNIVERSITY | 1 | 0.035 |
| YEE ZEN GEN HOSP | 1 | 0.035 |
| YENI YUZYIL UNIVERSITY | 1 | 0.035 |
| YEOVIL DIST GEN HOSP | 1 | 0.035 |
| YEOVIL DIST HOSP | 1 | 0.035 |
| YEUNGNAM UNIVERSITY | 1 | 0.035 |
| YIDU CENT HOSP WEIFANG | 1 | 0.035 |
| YILDIRIM BEYAZIT UNIVERSITY | 1 | 0.035 |
| YINZHOU 2 HOSP | 1 | 0.035 |
| YONSEI UNIVERSITY HEALTH SYSTEM | 1 | 0.035 |
| YORK UNIVERSITY CANADA | 1 | 0.035 |
| YOSHITOMI PHARMACEUT IND LTD | 1 | 0.035 |
| YUAN ZE UNIV | 1 | 0.035 |
| YUNNAN UNIVERSITY OF CHINESE MEDICINE | 1 | 0.035 |
| ZABLUDOWICZ CTR AUTOIMMUNE DIS | 1 | 0.035 |
| ZHEJIANG A F UNIVERSITY | 1 | 0.035 |
| ZHEJIANG PROVINCIAL PEOPLE S HOSPITAL | 1 | 0.035 |
| ZHENJIANG FIRST PEOPLES HOSP | 1 | 0.035 |
| ZIEKENHUIS GRP TWENTE | 1 | 0.035 |
| ZIV MEDICAL CENTER | 1 | 0.035 |
| ZUNYI MEDICAL UNIVERSITY | 1 | 0.035 |
