# Supplementary figures and images for: Bibliometric Analysis of Research on the Comorbidity of Pain and Inflammation
Source: Pain Res Manag. 2021 Feb 17;2021:6655211. doi: 10.1155/2021/6655211 (PMC7904349; doi:10.1155/2021/6655211)

**Supplementary Figure 1: Overview of the paper selection process.**


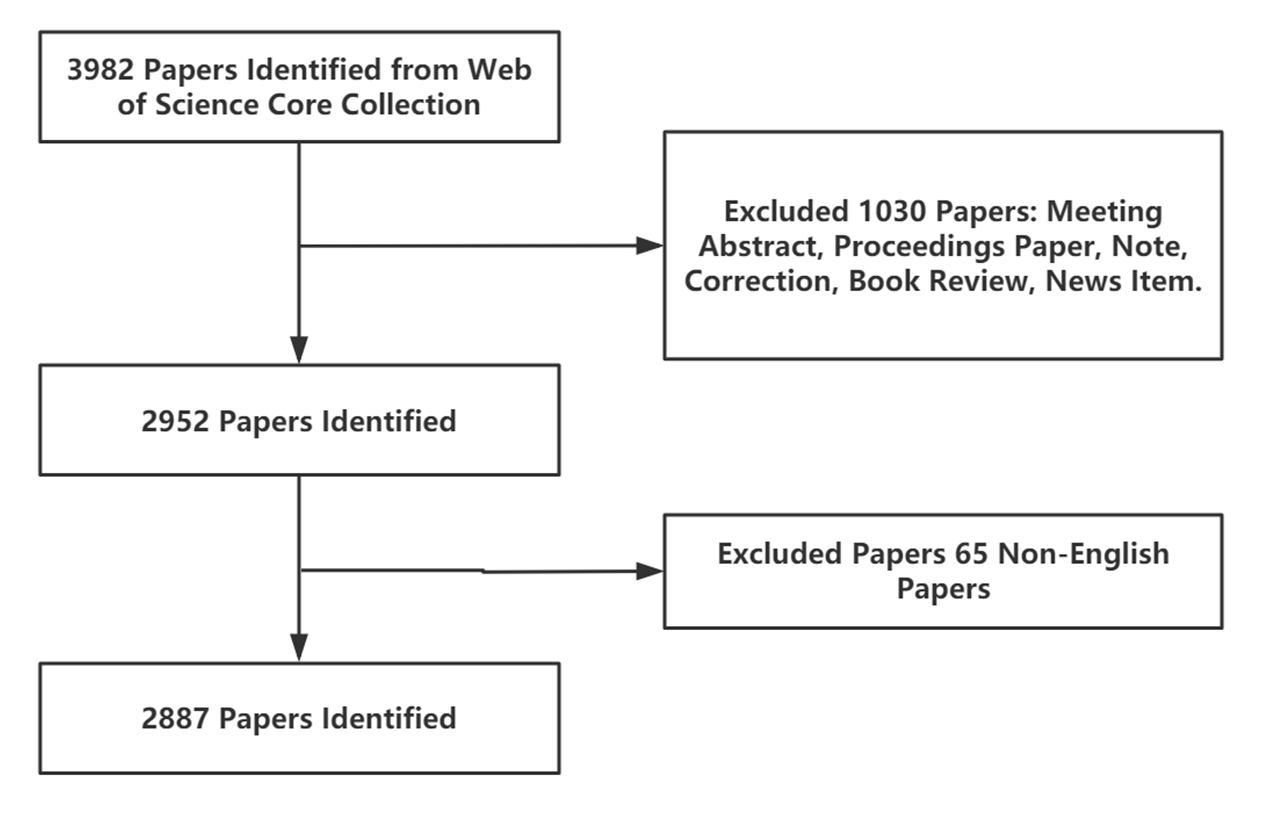

Supplement: Supplementary Materials — Supplementary Figure 1: overview of the paper selection process.Supplementary Figure 2: the number of papers, citations, citations per paper, open access papers, and H-index of the top 10 institutions. Supplementary Table 1: raw data on countries/territories involved in pain and inflammation publications. Supplementary Table 2: raw data on institutions involved in pain and inflammation publications. [file 6655211.f1.zip › 6655211.f1/Supplementary Figure 1.docx]
